# Intraspecific variation in plant‐associated herbivore communities is phylogenetically structured in Brassicaceae

**DOI:** 10.1111/ele.13852

**Published:** 2021-07-30

**Authors:** Daan Mertens, Klaas Bouwmeester, Erik H. Poelman

**Affiliations:** ^1^ Laboratory of Entomology Wageningen University and Research Wageningen The Netherlands; ^2^ Biosystematics Group Wageningen University and Research Wageningen The Netherlands

**Keywords:** cabbage and mustard family, ecological interaction networks, plant defence strategies, trait variation, uncertainty of herbivore attack

## Abstract

As a result of co‐evolution between plants and herbivores, related plants often interact with similar herbivore communities. Variation in plant–herbivore interactions is determined by variation in underlying functional traits and by ecological and stochastic processes. Hence, typically, only a subset of possible interactions is realised on individual plants. We show that insect herbivore communities assembling on individual plants are structured by plant phylogeny among 12 species in two phylogenetic lineages of Brassicaceae. This community sorting to plant phylogeny was retained when splitting the community according to herbivore feeding guilds. Relative abundance of herbivores as well as the size of the community structured community dissimilarity among plant species. Importantly, the amount of intraspecific variation in realised plant–herbivore interactions is also phylogenetically structured. We argue that variability in realised interactions that are not directly structured by plant traits is ecologically relevant and must be considered in the evolution of plant defences.

## Graphical abstract

As a result of co‐evolution between plants and herbivores, related plants often interact with similar herbivore communities. We show that insect herbivore communities assembling on individual plants and the amount of intra‐specific variation in realised plant–herbivore interactions are structured by plant phylogeny. We argue that this structure is ecologically relevant and must be considered in the evolution of plant defences.

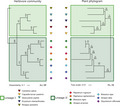

## INTRODUCTION

Over their lifetime, plants interact with numerous organisms of which many are herbivorous. In addition to a mixture of environmental effects, such as host plant community composition and abiotic conditions, the set of antagonistic species interacting with a plant species is determined by selection pressures and phylogenetic history (Hutchinson et al., 2017; Morales‐Castilla et al., 2015). Insect herbivores may exert selection that promotes novel defence strategies in plants and these events often coincide with radiation of herbivore species that have counteradaptations to these novel defences (Edger et al., 2015; Ehrlich & Raven, 1964). Even though the relationship between plant phylogenetic distance and overlap in herbivore communities is variable among plant clades, co‐evolutionary processes often result in non‐random structuring of plant‐associated antagonist communities (Bergamini et al., 2017; Cirtwill et al., 2020; Rapo et al., 2019).

While phylogenetic conservatism in herbivory is ubiquitous, few herbivore species are limited to a single host plant species in their trophic niche (Forister et al., 2012; Mooney et al., 2012). This may cause plant phylogenetic patterns to dissipate at higher taxonomic resolutions. However, in many plant species, there is a discrepancy between the full potential of antagonistic interactions at the plant species level and the subset of realised interactions for individual plants (Kuppler et al., 2016; Lewinsohn et al., 2005). The subset of antagonists actually colonising individual plants is determined by intraspecific variation in plant traits (Barbour et al., 2018; Barbour et al., 2015; Visakorpi et al., 2019), variation in functional traits among antagonist species or individuals (Zytynska & Preziosi, 2013), priority effects in community assembly in which a first coloniser affects the likelihood of attack by other antagonists (Lill & Marquis, 2003; Stam et al., 2018), environmental heterogeneity and habitat filtering (Agrawal & Fishbein, 2006; Johnson & Agrawal, 2005; Violle et al., 2012) as well as stochastic processes (Barber & Marquis, 2011; Shoemaker et al., 2020). Variation in the prevalence with which specific interactions occur on individual plants can reveal plant phylogenetic structuring of antagonist communities among more closely related plant species or even among populations of a plant species (Awmack & Leather, 2002; Johnson & Agrawal, 2005; Salazar et al., 2016b).

Evaluating variation in the full potential of interactions at the plant species level, the number of realised interactions at the level of individual plants and how these two measures relate can provide insight into the extent that plant–herbivore interactions entail closely coevolved systems (Wootton & Emmerson, 2005). For example, plant species that interact with a larger species pool of herbivores face a larger number of potential herbivore species at the level of individual plants, possibly resulting in more substantial variation in the interactions realized on individual plants. This intraspecific variation in realised plant–insect interactions may identify which plant species or populations are characterised by increased variation in the functional plant traits underlying interactions with antagonists (Ibanez et al., 2016; Ohgushi, 2016; Salazar et al., 2016a). Importantly, variation in the interactions individual plants experience that is not directly structured by plant traits may be relevant in the evolution of plant defence strategies. However, this variation has largely been ignored in plant defence theory (Karban, 2019; Mertens et al., 2021; Poelman & Kessler, 2016). Plants evolved inducible defences that are activated upon recognition of attack to save metabolic costs associated with production and maintenance of defence when herbivores are absent (Meldau et al., 2012). Plasticity in defence also allows plants to tailor defences to specific antagonists and to integrate responses to multiple attackers (Thaler et al., 2012; Van der Ent et al., 2018). Variation in interactions with herbivores that are not predominantly structured by plant functional traits may impact the evolution of such plastic defence strategies. It is to be expected that related plant species face more similar levels of variation in their interactions as closely related plant species tend to interact with herbivore communities that are similar in composition (Agosta, 2006; Futuyma & Agrawal, 2009; Futuyma & Mitter, 1996). However, it is unknown whether a phylogenetic structuring of the amount of variation in communities of antagonists on individual plants exists.

To determine the phylogenetic structuring of plant–herbivore communities and evaluate how the full potential of interactions at the plant species level relate to the number of realised interactions at the level of individual plants, we compare insect herbivore community characteristics among 12 Brassicaceae species, belonging to two major phylogenetic lineages (Lineage I and Lineage II) within this family. The two lineages derived from a common ancestor about 20 million years ago and differ in composition and diversity of defence chemistry to insect herbivore community (Abrahams et al., 2020; Murat et al., 2015) that underlies radiation of some of its specialist insect herbivores (Edger et al., 2015). We hypothesise that the composition of the herbivore community with which plants interact correlates with plant phylogeny and that this phylogenetic structure is retained in the amount of variation in interactions observed on individual plant levels. We explicitly test whether plant phylogeny predicts (i) the species richness, diversity and the proportion of realised interactions on individual plants out of the full potential herbivore community, (ii) the similarity in the average herbivore community composition and structure on individual plants within a species, (iii) the role of specific plant development traits in structuring the intraspecific variation in antagonistic interactions plants experience and discuss its consequences for the evolution of plant defence strategies.

## MATERIALS AND METHODS

### Study system and plant rearing

We monitored the herbivore community associated with 12 annual Brassicaceae species. We selected these 12 species for their overlapping niches and to represent the most speciose evolutionary Lineages I and II of the Brassicaceae native to the Netherlands (Beilstein et al., 2008) (Figure S1, Table S1). Seeds were sown on peat soil (Lentse Potgrond) and germinated under glasshouse conditions. One‐week‐old sprouts were transplanted into peat soil cubes. One week prior to the start of the field experiment, plants were allowed to acclimatise to field conditions under a roofed shelter. Four‐week‐old plants were transplanted to the experimental field (mid‐May; week 22 of 2016).

### Experimental site and design

The study site was located on the experimental fields of Wageningen University, The Netherlands (51°59′26.5″N, 5°39′50.5″E). The experimental fields are embedded in an agricultural and grassland landscape with a variety of brassicaceous herbs, ensuring the presence of a large species pool of insect herbivores part of a typical community on Brassicaceae (Tables S2 and S3). We installed a common garden experiment consisting of 120 plots organised in a rectangle of 10 columns by 12 rows. Plant species were randomly assigned to one of the 12 plots within a column, resulting in 10 replicate plots per plant species. Plots measured 3 × 3 m and contained nine individual plants in monoculture planted 1 m apart. Plots were separated from each other and the field edge by 4‐m‐wide grass lanes. To obtain edge uniformity, we planted a strip of *Brassica nigra* (six plants per square meter) around the experimental field. Kites and a meshed fence were placed to prevent damage by vertebrate herbivores. Plots were regularly weeded, and the grass lanes were mown every other week.

### Field observations of herbivores and plant growth

Herbivore communities were monitored on five central plants per plot (i.e. excluding the four corner plants). In cases where a central plant died before the second monitoring round, we monitored one of the corner plants. We recorded naturally occurring herbivores interacting with these plants by weekly counts early in the season and by biweekly counts later in the season. Classification of arthropods as herbivores was done based on pre‐existing knowledge of the study system and/or by observing herbivory in the field (Table S3). Community development on individual plants was surveyed until seed set. To allow the community to fully develop, insects were identified in situ to the highest taxonomic resolution possible (species, genus or family level), by means of anatomical characteristics and expert knowledge (Table S2).

In addition to arthropod observations, we recorded a set of phenotypic parameters for all visited plants during each monitoring round: plant height (measured from the ground to the top of the plant), diameter (measured as the distance between the two most distal leaves), length of the largest leaf, number of true leaves and number of flowering branches. The plant traits that were measured can readily be hypothesised to affect the herbivore community, as they determine the apparency of plants (e.g. plant height) or the availability of specialised niches (e.g. number of flowering branches). In addition, these measurements are non‐destructive and ensure a minimal disturbance of the assembly of the plant‐associated herbivore community. In the subsequent analyses, we used the maximum observed parameter values for each plant individual to represent its specific phenotype.

### Reconstruction of plant phylogeny

ITS sequences of the 12 brassicaceous species and the out‐group *Aethionema arabicum*, a sister species of the core Brassicaceae, were retrieved from the BrassiBase website (https://brassibase.cos.uni‐heidelberg.de). Multisequence alignments were obtained by MAFFT v7 using the iterative refinement method FFT‐NS‐i with a gap opening penalty of 1.0. Unreliable alignment regions were detected with GUIDANCE2 using 100 bootstraps at default thresholds. Residues and columns with a confidence score >0.650 were removed. Phylogenetic relationships were inferred by maximum likelihood (ML) and Bayesian methods. ML analyses were computed with W‐IQ‐TREE using the best‐fit model (SYM + G4) selected by ModelFinder and 1000 bootstrap replicates. Bayesian interference analysis was conducted with MrBayes 3.2.6 using the GTR substitution model under default priors. Chains were run for 100,000 generations and trees were sampled every 100 generations. The initial 1000 trees were discarded as burn‐in, and posterior probabilities (PP) were calculated from the remaining replicates. Phylogenetic trees were drawn and edited in iTOL 4.4 (Figure S1).

### Data analysis

To test for the plant phylogenetic structuring of herbivore communities on the level of plant species or plant individuals, we explored the incidence‐based as well as the abundance‐based community data set and its subsets focusing on the community of sap‐feeding herbivores or chewing herbivores. Previous studies have shown that feeding guilds differ in their interaction with Brassicaceae, are different in their level of host specialization and are governed by different processes at both regional and local scales (Lewinsohn et al., 2005; Soler et al., 2012). From these data sets, we derived matrices that present observations at the biological level of plant species, or which are transformed to adjust for potential biases that are inherent to the biology of the herbivore species in our community. An overview of the different matrices and the analyses in which they were used is presented in Table S4.

To frame diversity in the context of interactions among species, we calculated a set of interaction network metrics: network connectivity C (Delmas et al., 2019), nestedness (weighted NODF; Almeida‐Neto et al., 2008; Almeida‐Neto & Ulrich, 2011) and specialisation (H2' and *d_i_* '; Blüthgen et al., 2006, 2008) of the plant species–herbivore interaction networks (Table S4). We tested the network metrics by comparing them to two separate null models based on Patefield (Patefield, 1981) and Vaznull algorithms (Vazquez et al., 2007), respectively. We generated 999 random networks for each of the algorithms and used one‐sample *t*‐tests with our observed network descriptor as a reference to estimate significance (Blüthgen et al., 2008; Flores et al., 2011).

We then calculated species richness, the exponential and log‐based Shannon diversity indices and Simpson diversity for each plant species and on the level of each plant individual (Heip et al., 1998) (Table S4). The number of herbivore species that could interact with a plant species was calculated by summing all observed herbivore species per plant species. In addition, we compared the proportion of realised interactions by relating the richness of the species pool that could interact with a plant species with the richness of herbivore communities observed on individual plants of that species (Whittaker's β diversity). These community properties were tested for a phylogenetic structure by applying (generalised) linear (mixed) effect models ((G)L(M)M) with the respective diversity measure as response variable and either plant species or lineage as explanatory variables. Mixed effect models included plot identity, and when testing for differences among plant lineages, plant species as random intercepts. We evaluated sampling completeness (Chao & Jost, 2012), assessed sample‐based species rarefaction curves and compared diversity measures at interpolated and, where relevant, at extrapolated estimations of sample completeness (Figure S2; Table S5) (Gotelli & Colwell, 2001).

Herbivore communities were further characterised by the dissimilarity in herbivore species composition between plant individuals of the same species (expressed by multivariate Sørensen β diversity). The Sørensen dissimilarity has a direct correspondence with multivariate dispersion in community composition (Anderson et al., 2011; Anderson et al., 2006) and can be decomposed into a turnover component (i.e. dissimilarity due to species replacement) and a nestedness component (i.e. dissimilarity due to loss or gain in species richness) (Baselga, 2010, 2012).

We used multivariate ordinations (non‐metric multidimensional scaling) of the herbivore community on individual plants to explore the variation in composition and structure of herbivore communities associated with plants across the different plant species and lineages. We assessed community composition based on the Sørensen dissimilarity matrix of incidence observations (NMDS: three ordinal dimensions, stress = 0.18) and community structure by the Bray–Curtis dissimilarity matrix of log (*x* + 1) transformed abundance observations (NMDS: three ordinal dimensions, stress = 0.19) (Table S4). We then estimated the overall dissimilarity in herbivore communities explained by plant species and lineages by permutational analyses (PERMANOVA) (Anderson, 2001). To ensure the valid permutation of communities, we specified the structure of our experiment in the permutational design. Statistical significance was assessed via 999 permutations. Post‐hoc comparisons between plant species were made by running a separate PERMANOVA analysis for each comparison and limiting the proportion of Type I errors by false discovery rate control (Verhoeven et al., 2005). We used SIMPER analysis to identify which herbivore species contributed most to the differences detected among plant species, and if this contribution was significant (999 permutations; Table S4) (Clarke, 1993; Clarke & Warwick, 2001).

We then applied Mantel tests to quantify the correlation between the phylogenetic similarity of plant species and the similarity in herbivore communities on plants (Legendre & Legendre, 1998; Mantel, 1967). To visually compare the relation between the similarity of herbivore communities and relatedness of plant species, we ran an unconstrained principal component analysis (PCA) on the abundance‐based community data (Table S4) and calculated the multivariate coordinates of the centroids of each of the plant species. We then used these coordinates in cluster analysis (complete linkage, Euclidean distance) and verified the clusters by using a multiscale bootstrap procedure (1000 bootstraps) (Suzuki & Shimodaira, 2006). We repeated the cluster analysis on centroids obtained from a PCA on Hellinger‐transformed abundance data to account for differences in the size of communities on individual plants (Legendre & Gallagher, 2001).

We then related inter‐ and intraspecific variation in herbivore communities to the phenotypic traits measured for plant individuals. To allow the comparison of different traits, we scaled each of the traits to zero mean and unit variance. As similarity in plant phenotypes are likely to be higher for closely related species (Blomberg & Garland, 2002), we first tested the correlation between phenotypic traits and phylogenetic relatedness by applying a Mantel test. We then ran an unconstrained PCA ordination on our community data, followed by post‐hoc regression of the three most important PCA axes by the plant‐phenotypic traits (Table S4). In an alternative approach, we constrained the variation in the herbivore community with the measured plant phenotype by applying a stepwise redundancy analysis (RDA) procedure with 999 random permutations per step (Šmilauer & Lepš, 2014; Van den Wollenberg, 1977). To determine whether the relation between plant phenotype and the associated communities differed among plant species, we repeated the stepwise RDA procedure for each plant species separately.

All statistical analyses were performed using R (v3.2.4) (R Core Team, 2014) packages nlme (Pinheiro et al., 2012), lme4 (Bates et al., 2015), emmeans (Lenth et al., 2019), vegan (Oksanen et al., 2012), BiodiversityR (Kindt & Coe, 2005), bipartite (Dormann et al., 2009; Dormann et al., 2008) and pvclust (Suzuki & Shimodaira, 2006).

## RESULTS

### Plant phylogeny structures the number and proportion of realised interactions on individual plants

**FIGURE 1:**
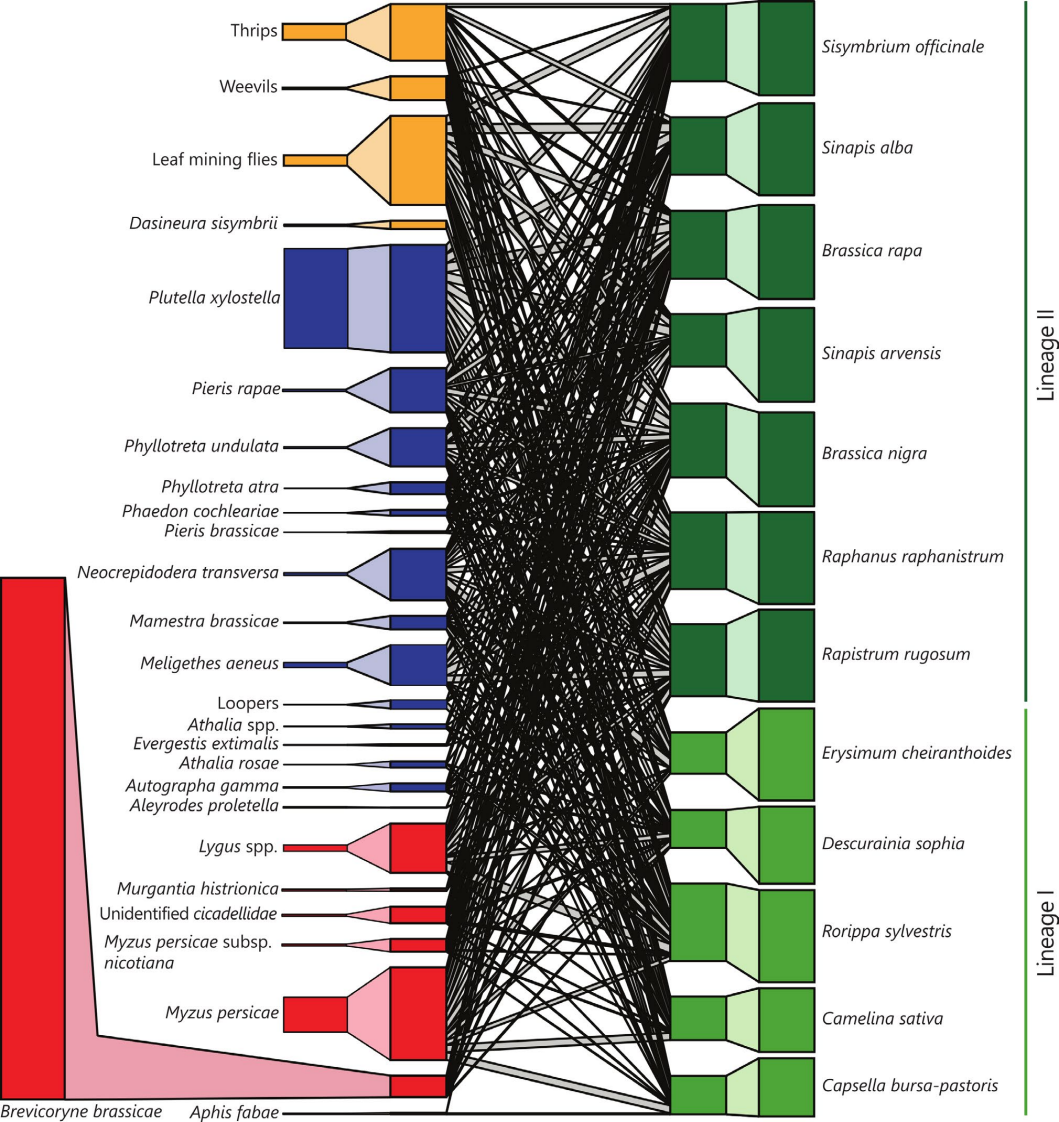
Interactions between Brassicaceae and their herbivore communities are characterised by low levels of specialisation. Bipartite network of the total plant–herbivore community observed in our common garden experiment. Boxes on the outside of the diagram represent relative abundances of herbivores and plants. Boxes on the inside of the diagram represent interaction frequencies adjusted for uneven sampling of plant individuals (i.e. incidence‐based network). Shaded areas between boxes on the outside of the diagram and on the inside of the diagram (within the same trophic level) depict the association between relative abundance and standardised prevalence in the field. Lines connecting herbivore species and plant species represent realised interactions, and the width of these lines represents the relative number of (incidence) interactions. Colours of boxes represent sap‐feeding herbivores (red), chewing herbivores (blue) and unclassified herbivores (yellow) on the trophic level of herbivores and Brassicaceae species belonging to Lineage I (light green), and Lineage II (dark green) at the trophic level of plant species

By repeatedly monitoring insect herbivore communities during the life span of individual plants of 12 annual Brassicaceae species, we identified that the network of insect herbivores interacting with the 12 plant species was highly connected (Figure 1). Overall, both the interaction network based on herbivore incidence, as well as its abundance‐based equivalent were characterised by high levels of connectance and nestedness and low levels of interaction specialisation (Tables S6–S9). The two phylogenetic lineages of Brassicaceae showed no distinct subgroups of interactions and the insect herbivore species pools were largely shared among all plant species (Figure 1). Plant species in our network showed significantly higher levels of diversity in their interactions with herbivore species compared with expectations inferred by two separate null models. Specialisation remained low and was dependent on the subset of the herbivore community under investigation. Plant species‐specific levels of specialisation in their interactions with herbivores (expressed by Blüthgens' *d_i_* ') were highest for the abundance‐based network analysis of the sap‐feeding herbivores (Table S9). The increased specialisation of plant interactions with this community subset is likely driven by the exponential increase in population size of aphids on plants they successfully colonise, mimicking patterns of increased interaction specialisation (Table S10).

Although plant species differed in the diversity of their full herbivore communities, these patterns were generally not structured among the two phylogenetic lineages of Brassicaceae (Tables S5 and S11). The most apparent plant phylogenetic structure was found for higher species richness of sap‐feeding herbivores on plant species of Lineage II compared with those belonging to Lineage I (LM: df = 1; *F* = 10.74; *p* = 0.0083). However, the full herbivore species pools associated with Brassicaceae species of Lineage II were not statistically different in size or diversity compared with herbivore communities associated with species of Lineage I. None of the abundance‐based diversity measures (i.e. Shannon and Simpson indices) calculated for the community subset of sap‐feeding herbivores nor the diversity measures calculated for the subset of chewing herbivores were significantly different among lineages (Table S11).

**FIGURE 2:**
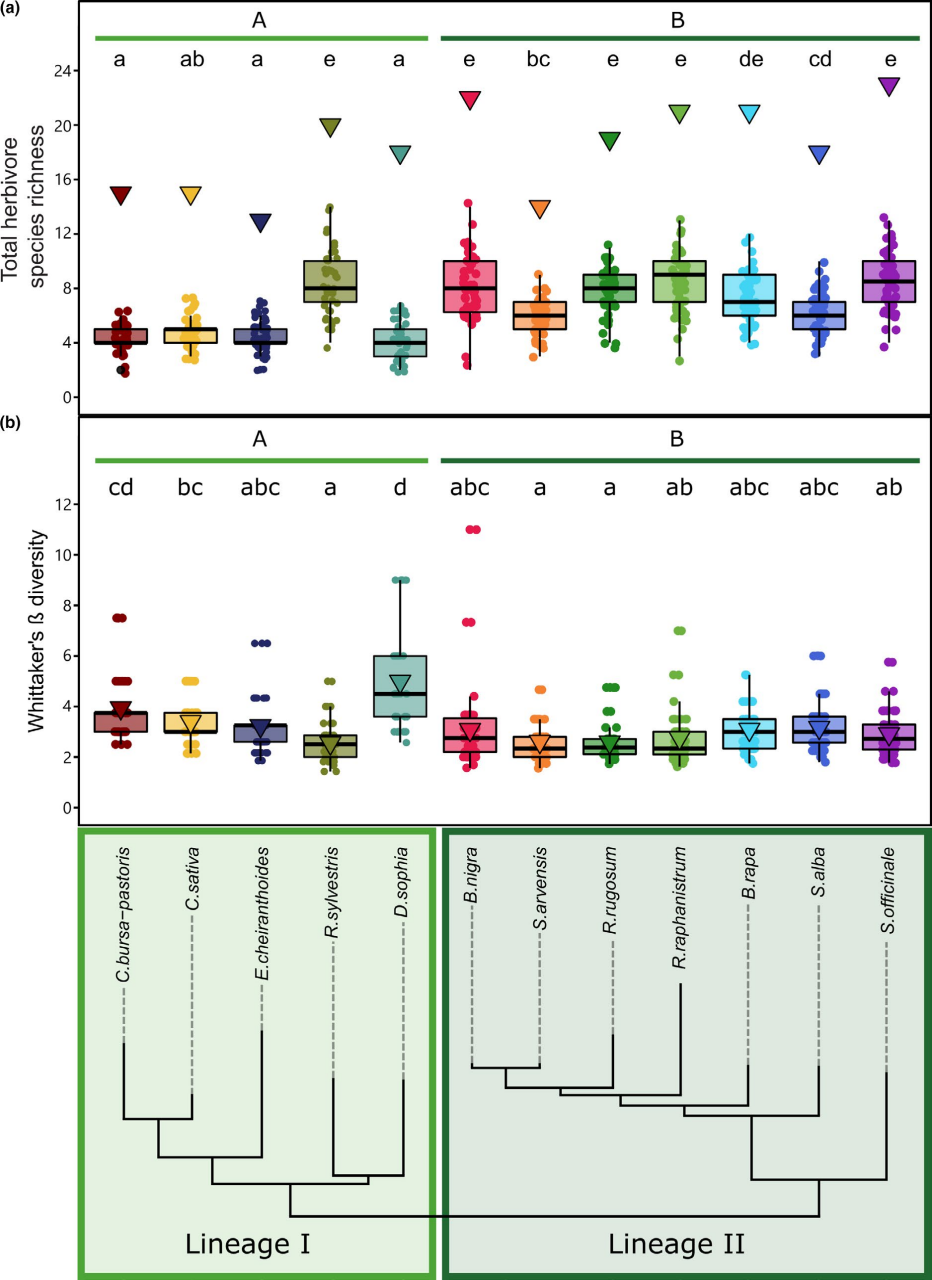
Herbivore diversity and uncertainty of attack on individual plants is dependent on plant species and structured by plant phylogeny. (A) Herbivore species richness on individual plants correlates with plant phylogenetic lineages and (B) the proportion of the herbivore species richness at the plant species level and the number of realised interactions on individual plants (Whittaker's β) as measures of variation in herbivore communities on individual plants observed across plant phylogenetic lineages (lower panel). Triangles depict the total number of herbivore species and the average β diversity associated with the total number of individuals per plant species, respectively. Dots represent the number of herbivore species or the β diversity observed on individual plants of the respective plant species. Box‐whiskers summarise the variation in observations at the level of plant individuals. Statistical analyses were performed by applying linear mixed models with species or phylogenetic lineage as explanatory factors and including plot and, when estimating the diversity for phylogenetic lineages, plant species as random factors in our models. To account for the heterogeneity of variance, we allowed the variance to be different for the different species or lineages in our model. Different letters indicate significantly different means (*p* < 0.05), adjusted for multiple testing by Tukey HSD. Significant differences among lineages (plant species grouped by the coloured horizontal bars) are indicated with capital letters. Statistical analyses were performed separately for the different panels

In contrast to the absence of a strong plant phylogenetic structure in the diversity of the full pool of insect herbivores associated with plant species, the realised interactions with herbivores on individual plants were structured by plant phylogenetic lineage. Individual plants of species in Lineage II were attacked by a more species‐rich herbivore community than plants of species belonging to Lineage I (Figure 2; Tables S5, S12 and S13). The higher species richness was not dependent on the herbivores' feeding guild, showing that individual plants in Lineage II interacted with a more species‐rich chewer community as well as a more species‐rich sap‐feeding herbivore community (Figure S3). A marginally lower proportion of potential interactions (expressed by Whittaker's β diversity) was realised on individual plants of Lineage II than Lineage I (LMM: df = 1; *ꭓ* ^2^ = 4.41; *p* = 0.0357) (Figure 2). As the proportion of realised interactions was comparable across the two plant lineages and richness of realised as well as potential interactions was higher for plants in Lineage II, plants of Lineage II are characterised by more substantial variation in the realised interactions they encounter. Across all 12 brassicaceous species, variation in the identity of attackers as illustrated by variation in herbivore community composition on individual plants was generally high and primarily caused by differences in the identities of herbivores rather than variation in the number of herbivore species attacking individual plants (Figure S4; Table S14). Plant species also varied significantly in the values of the Shannon and Simpson indices observed for individual plants, identifying differences in evenness of community structure across plant species (Figures S5 and S6, Tables S12 and S13). Except for a significantly higher log‐based Shannon index associated with chewing herbivore communities on plant species belonging to Lineage II, these indices were not significantly different between the two lineages.

Overall, these results indicate that individual plants of Lineage II were exposed to more substantial variation in interactions with attackers than plants of Lineage I and that this higher level of variation in interaction partners is mainly driven by the high absolute richness of potential species interactions and the high number of realised interactions per plant individual.

### Individual plants of different species differ in herbivore community composition and structure

**FIGURE 3:**
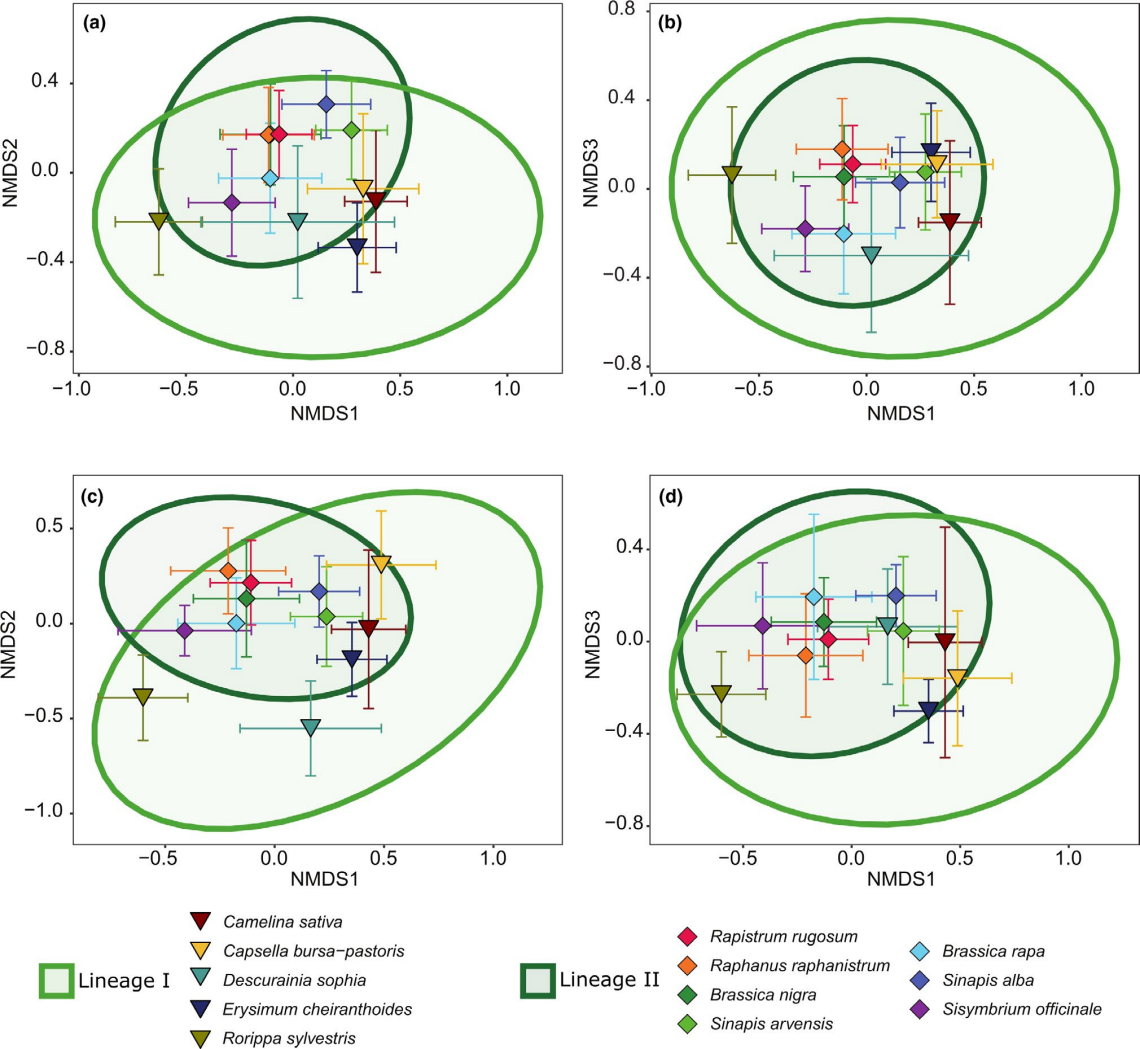
Composition and structure of herbivore communities differ among plant species and plant lineages. Ordination of observed herbivore community composition (expressed by the incidence of herbivores, panels A and B) and structure (expressed by log (*x* + 1) transformed cumulative herbivore abundance data, panels C and D), according to three NMDS ordination axes (stress = 0.18 and 0.19, respectively). Triangles and diamonds represent the centroid of the variation in communities associated with plants belonging to Lineages I and II, respectively, and are coloured according to plant species. Error bars around the plant centroids represent the 95% confidence interval around the estimation of the centroid. Ellipses are coloured according to plant lineage and depict the 95% interval of a multivariate *t* distribution around the centroids of each of the two plant lineages

**FIGURE 4:**
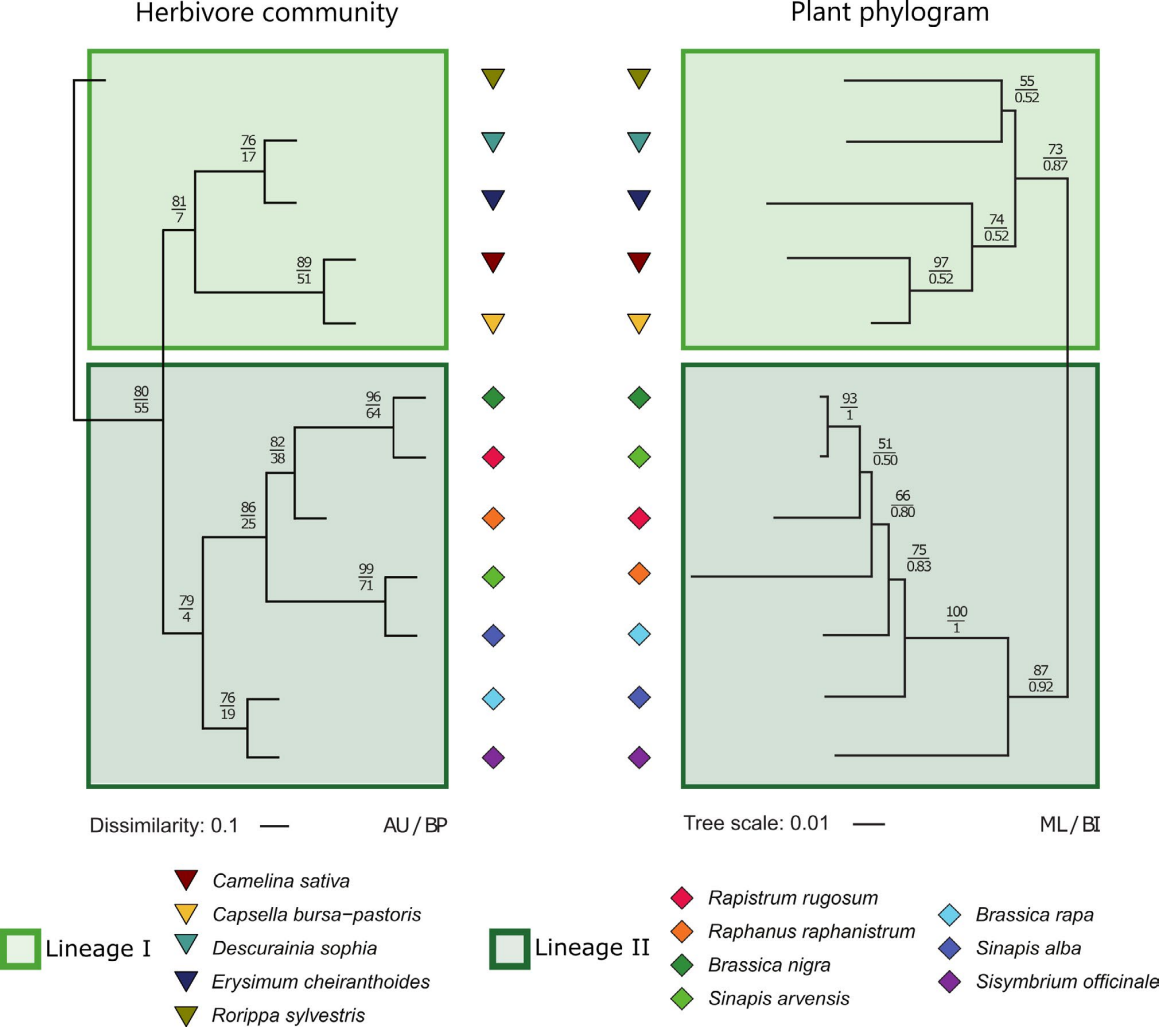
Average multivariate structure of herbivore communities matches with plant phylogeny. Comparison between cluster analysis of the centroids of the log (*x* + 1) transformed cumulative herbivore abundance data as calculated from PCA coordinates and the Maximum Likelihood phylogram of Brassicaceae species inferred from ITS sequences. Values of approximately unbiased (AU) and bootstrap probability (BP) are displayed in the cluster analysis, and Bootstrap support values (BS) >50% and Bayesian posterior probabilities (PP) >0.5 are displayed in the phylogram. Scale bars indicate the Euclidean dissimilarity among centroids and the proportion of sites along each branch, respectively

The average composition of herbivore communities on individual plants differed between plant species and between phylogenetic Lineages I and II. These differences were emphasised when taking the community structure into account (Figure 3A,B, Table 1). The plant phylogenetic structuring of herbivore communities on individual plants was further supported by a significant correlation between the dissimilarity in community composition and structure among individual plants and their phylogenetic dissimilarities at the species level (Figure 4, Table 2). This correlation was less evident when standardising communities to account for differences in the total abundance of herbivores on individual plants, indicating that variation in both the relative abundance of specific herbivores as well as the size of the community is important in structuring the dissimilarity among plant species (Figure S7). Pairwise comparisons of realised communities on individual plants were almost always significantly different between plant species (Tables S15 and S16). SIMPER analysis revealed that the contribution of any single herbivore species to the dissimilarity among plant species was on average <5% and was dependent on the plant species compared (Tables S17 and S18). Overall, these results indicate that plant species and phylogenetic lineages were characterised by the average herbivore community associated with individual plants (Figure 4).

**TABLE 1:** Differences between plant lineages or plant species in the composition or structure of the herbivore community associated with plants are estimated by PERMANOVA analysis

Community subset	Biological level	Composition			Structure		
		df	Pseudo‐*F*	*p*	df	Pseudo‐*F*	*p*
Full herbivore community	Lineage	1	63.04	**0.0070**	1	85.03	**0.0010**
	Plant species	11	20.32	**0.0010**	11	24.54	**0.0010**
Chewing herbivore community	Lineage	1	92.68	**0.0050**	1	88.57	**0.0010**
	Plant species	11	18.58	**0.0010**	11	21.96	**0.0010**
Sap‐feeding herbivore community	Lineage	1	26.73	**0.0030**	1	22.93	**0.0100**
	Plant species	11	21.40	**0.0010**	11	21.32	**0.0010**

Notes

Community composition was analysed by incidence observations, and community structure was assessed by analysing log(*x* + 1) transformed cumulative abundance observations. The analysis was performed separately for the full herbivore community, the chewing herbivore community and the sap‐feeding herbivore community associated with plants. Significant *p* values (*p* < 0.05) are indicated in bold and are assessed by 999 random permutations while taking the dependency of observations in our experiment into account.

**TABLE 2:** Correlation between the herbivore community composition or structure of plants and their phylogenetic similarity inferred from ITS sequences

Community subset	Composition		Structure	
	*r*_m_	*p*	*r*_m_	*p*
Full herbivore community	0.13	**0.0010**	0.16	**0.0010**
Chewing herbivore community	0.17	**0.0010**	0.14	**0.0010**
Sap‐feeding herbivore community	0.10	**0.0010**	0.12	**0.0010**

Notes

Correlations between the composition (expressed by incidence observations) or structure (expressed by log(*x* + 1) transformed cumulative abundance observations) of the herbivore community and plant phylogeny were quantified by calculating the overlap in Sørensen or Bray–Curtis similarity matrices (for community composition and structure, respectively), and the phylogenetic similarity of plants using a Mantel test. The analysis was performed separately for the full herbivore community, the chewing herbivore community and the sap‐feeding herbivore community associated with plants. Significant *p* values (*p* < 0.05) are indicated in bold and indicate that the correlation between matrices was significantly different from zero.

### Plant phenotype correlates with herbivore community composition and structure

Plant species differed significantly in phenotypic characteristics such as leaf size, number of leaves, plants size and lifetime (Table S19), as well as in their multivariate phenotypes (PERMANOVA; df = 11; Pseudo‐*F* = 40.59; *p* = 0.001). These differences correlated significantly but weakly with their phylogenetic dissimilarity (Mantel test: *r*_m_ = 0.1490; *p* = 0.001). Phylogenetic structure in the variation of specific plants development parameters such as the plant diameter and leaf size were apparent among the two lineages (Table S20) and the multivariate phenotype was consistently different between the two lineages (PERMANOVA; df = 1; Pseudo‐*F* = 74.53; *p* = 0.001). Pairwise comparisons indicated that the multivariate phenotype of individual plants was significantly different among nearly all plant species (Table S21). Both unconstrained (PCA) and constrained ordination (RDA) analyses indicated that plant lifetime, size of the largest leaf and plant diameter were often associated with the largest proportion of variation in herbivore communities (Tables S22 and S23). The total amount of variation explained by the plant phenotypic parameters depends on the community subset (Constrained community composition: full: 18.73%; chewing: 18.00%; sap‐feeding: 23.03% and Constrained community structure: full: 26.17%; chewing: 20.88%; sap‐feeding: 24.91%). The percentage of intraspecific variation in herbivore communities that we could associate with plant phenotypic parameters was generally low and dependent on the plant species (Tables S24 and S25). Phenotypic traits explained a larger amount of variation in herbivore communities when taking the abundance of herbivore species into account (Tables S24 and S25). These results indicate that the weighted abundance of herbivore species in the community with which plants interact, rather than the identity of the herbivores, correlates more strongly with plant phenotypic parameters.

## DISCUSSION

Insect communities on individual plants of 12 Brassicaceae were structured by phylogenetic lineages of the Brassicaceae. Individual plants of species in Lineage II harboured a larger number of herbivore species than individual plants of species in Lineage I and dissimilarity in communities were further structured by differences in herbivore abundance. Our analyses revealed that the plant phylogenetic structure of insect communities may be revealed more prominently when focusing on realised interactions from a plant individual perspective rather than describing the full set of interacting species on the level of the 12 plant species in our experiment. Moreover, plant phylogeny predicts patterns in the amount of variation of antagonistic interactions at the level of individual plants. While the proportion of realised interactions per individual plant out of the full potential herbivore pool associated with a plant species is comparable across the two plant lineages, it represents a higher absolute number of interactions for plants in Lineage II due to the larger herbivore species pool associated with Lineage II plants. Thus, plants in Lineage II face more substantial intraspecific variation in terms of realised antagonistic interactions compared with plants in Lineage I.

Among the full set of organisms with which a single plant species interacts, phylogenetic relationships have been found to be dependent on the functional group of interaction partners and the type of ecological interactions (Cirtwill et al., 2020; Fontaine et al., 2009). For example, even though obligate specialists are more readily found in mutualistic interactions such as pollination, these networks are predominantly composed of more generalised interactions in which pollinators visit flowers of a large number of plants across families (Thébault & Fontaine, 2010; Waser et al., 1996). In antagonistic insect–plant interactions, herbivores are often adapted to specific plant families, genera or species, resulting in stronger specialisation of interaction networks across plant phylogeny (Fontaine et al., 2009; Thébault & Fontaine, 2010). However, for at least some plant families, using the full set of interacting species does not reveal deeper phylogenetic signals between plant genera or species and insect communities (Cirtwill et al., 2020). Similarly, the interactions observed for the 12 Brassicaceae plant species revealed low levels specialisation for all herbivore species. This may be explained by the presence of a unique class of defence chemicals (glucosinolates) in all Brassicaceae (Caballero et al., 2003), which has resulted in the dominance of specialist herbivores in Brassicaceae‐associated insect communities (Frenzel & Brandl, 1998; Root, 1973). This causes a large overlap in the composition of the species pools associated with each plant species within the Brassicaceae family (Lind et al., 2015; Novotny et al., 2002; Ødegaard et al., 2005). Based on our observations, only four herbivore species were found to be specific to Lineage II (*Pieris brassicae*, *Aleyrodes proletella*, *Evergestis extimalis* and *Murgantia histrionica*). A single herbivore species, the gall midge *Dasineura sisymbrii*, was exclusively observed on the Lineage I species *Rorripa sylvestris*. These observations may result in apparent specialisation in species interactions (i.e. high levels of *d_i_* ', see Table S10) due to rarity (e.g. *A*. *proletella*, which was only observed three times in our experiment) or due to context dependency of interactions between plants and herbivore species (Table S3) (Chamberlain et al., 2014; Costa et al., 2016; Poisot et al., 2015; Rivera‐Hutinel et al., 2012).

Nevertheless, a plant phylogenetic structuring of herbivore communities could be revealed at the lineage level within the Brassicaceae by analysing realised interactions on individual plants. Plant individuals from closely related species interacted with more similar but often distinct herbivore communities. Herbivores specialising in plants within the same family are more likely to select plants within a family by similarity in functional traits, which do not necessarily reflect (strong) phylogenetic similarity (Endara et al., 2017; Ibanez et al., 2016). In our study, variation in plant growth traits correlated with plant phylogeny structured the incidence of specific herbivore species on plants of different species and predicted weighted abundance of herbivores in realised communities. However, it is likely that these growth traits covaried with unmeasured but more important (suites of) functional traits such as the plant's composition of glucosinolates, presence of additional chemical classes such as cardenolides or production of volatiles, all of which potentially select for some specialisation in herbivore interactions (Barker et al., 2018; Blazevic et al., 2020; Züst et al., 2020). Intraspecific variation in plant functional traits or defence syndromes may affect the probability of interactions with specific antagonists, effectively splitting the full community associated with a plant species into subsets of antagonists with which interactions are more probable (Mertens et al., 2021). These subsets of antagonists may emerge through correlated responses of herbivore species to variation in plant traits or through priority effects in which the presence of one herbivore affects the course of insect community assembly (Kuppler et al., 2016; Stam et al., 2018). In our study, plant intraspecific variation in communities predominantly resulted from a turnover in herbivore species identity, while the species richness of communities interacting with plant individuals was less variable. These results identify that, to reveal deeper phylogenetic signals in plant–insect associations, studies should focus on realised interactions rather than potential interactions.

The larger species pool of antagonists associated with plant species and the higher species richness of communities realised on individual plants resulted in more substantial variation in antagonist communities on plants in Lineage II compared with plants in Lineage I. The phylogenetic organisation of variation in herbivore attack may be an important factor in the evolution of plant traits. We may predict that the larger variation in attack for Lineage II plants results in frequency‐dependent selection on plant functional traits and helps to maintain large variation in chemotypes that each interact with different communities. Additionally or alternatively, larger uncertainty of attack may select for plant defence plasticity with either stronger induced resistance or tolerance to herbivory in this lineage (Mertens et al., 2021). A key challenge will be to disentangle variation in realised interactions that are structured by variation in plant functional traits from variation in interactions that exist through processes that are not structured by plant traits. Our study identifies that the amount of variation in interactions with herbivores and/or stochasticity in attack is phylogenetically structured in Brassicaceae. This highlights the importance of phylogenetic analysis of plant plasticity to deepen our understanding of how variation in insect attack selects on plant defence strategies. An important goal of such phylogenetic analyses will be to obtain insight into the evolution of (variation in) the inducibility of defences among individual plants of related species and populations. The prevalence of a phylogenetic signal in realised herbivore communities that emerge through, for example frequency‐dependent selection on functional traits and the resulting compartmentalisation of herbivore communities, should be compared with relative strengths of local adaptation to more uncertain causes of variation in herbivore attack (Stamp & Hadfield, 2020). A final key challenge is to identify how evolution in insects explains the plant phylogenetic sorting of insect communities (Edger et al., 2015; Ehrlich & Raven, 1964).

## COMPETING INTEREST

The authors declare no competing interests.

## AUTHORSHIP

EHP conceived the study and designed the experiment, DM collected the data and performed the statistical analyses, KB performed phylogenetic analyses. All authors contributed substantially to the writing of the first draft and revisions of the manuscript.

### PEER REVIEW

The peer review history for this article is available at https://publons.com/publon/10.1111/ele.13852.

## Supporting information

Supplementary Material

## Data Availability

The data and code reported in this paper have been deposited at the Dryad public repository (https://doi.org/10.5061/dryad.4j0zpc88c). Preprint hosted by BioRxiv doi: https://doi.org/10.1101/2020.12.06.413724.
