# Supplementary material for: Intraspecific variation in plant‐associated herbivore communities is phylogenetically structured in Brassicaceae
Source: Ecol Lett. 2021 Jul 30;24(11):2314–27. doi: 10.1111/ele.13852 (PMC9291228; doi:10.1111/ele.13852)
Supplement: Supplementary file 1 — Supplementary Material [file ELE-24-2314-s001.docx]

**Supplementary information for**

Intra-specific variation in plant associated herbivore communities is phylogenetically structured in Brassicaceae

Daan Mertens^1^*, Klaas Bouwmeester^2^ and Erik H. Poelman^1^

^1^Laboratory of Entomology, Wageningen University and Research, P.O. Box 16, 6700 AA, Wageningen, The Netherlands.

^2^ Biosystematics Group, Wageningen University and Research, P.O. Box 16, 6700 AA, Wageningen, The Netherlands.

* Correspondence: Daan Mertens

**Email:** Daan.Mertens@wur.nl

**ORCID ID:** DM. 0000-0003-4220-9075; KB. 0000-0002-8141-3880; EHP. 0000-0003-3285-613X

**This PDF file includes:**

Figures S1 to S7

Tables S1 to S25

*
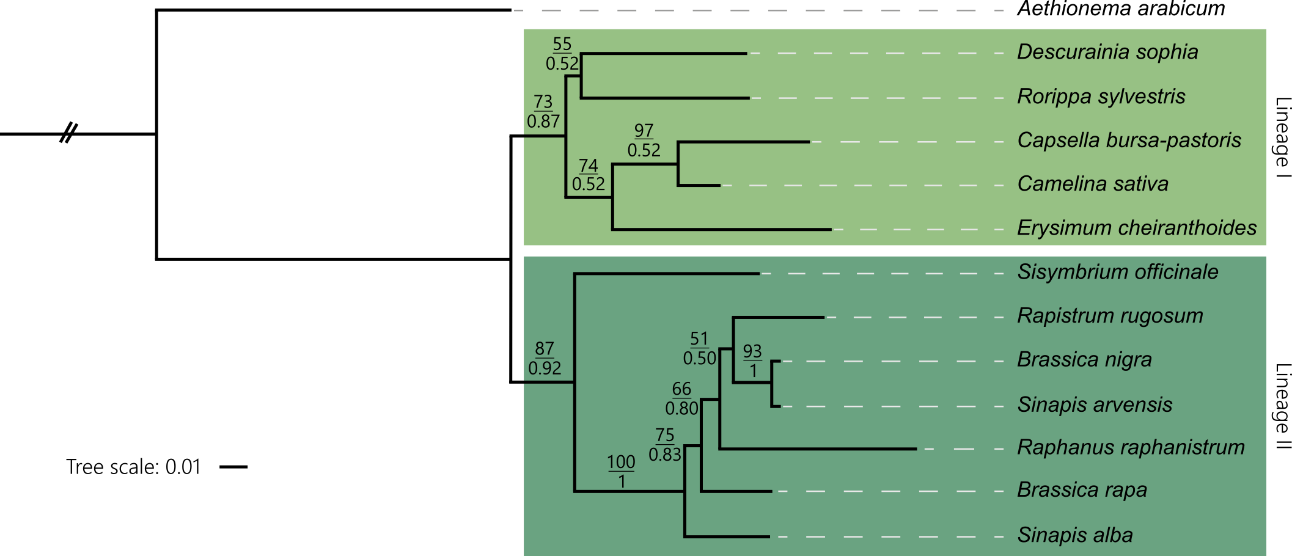
*

**Figure S1. Maximum likelihood phylogram of brassicaceous species inferred from ITS sequences.** The phylogenetic tree was rooted to the sister species. Bootstrap support values (BS) > 50% and Bayesian posterior probabilities (PP) > 0.5 are displayed at branches. Scale bar indicates the number of nucleotide substitutions per site.


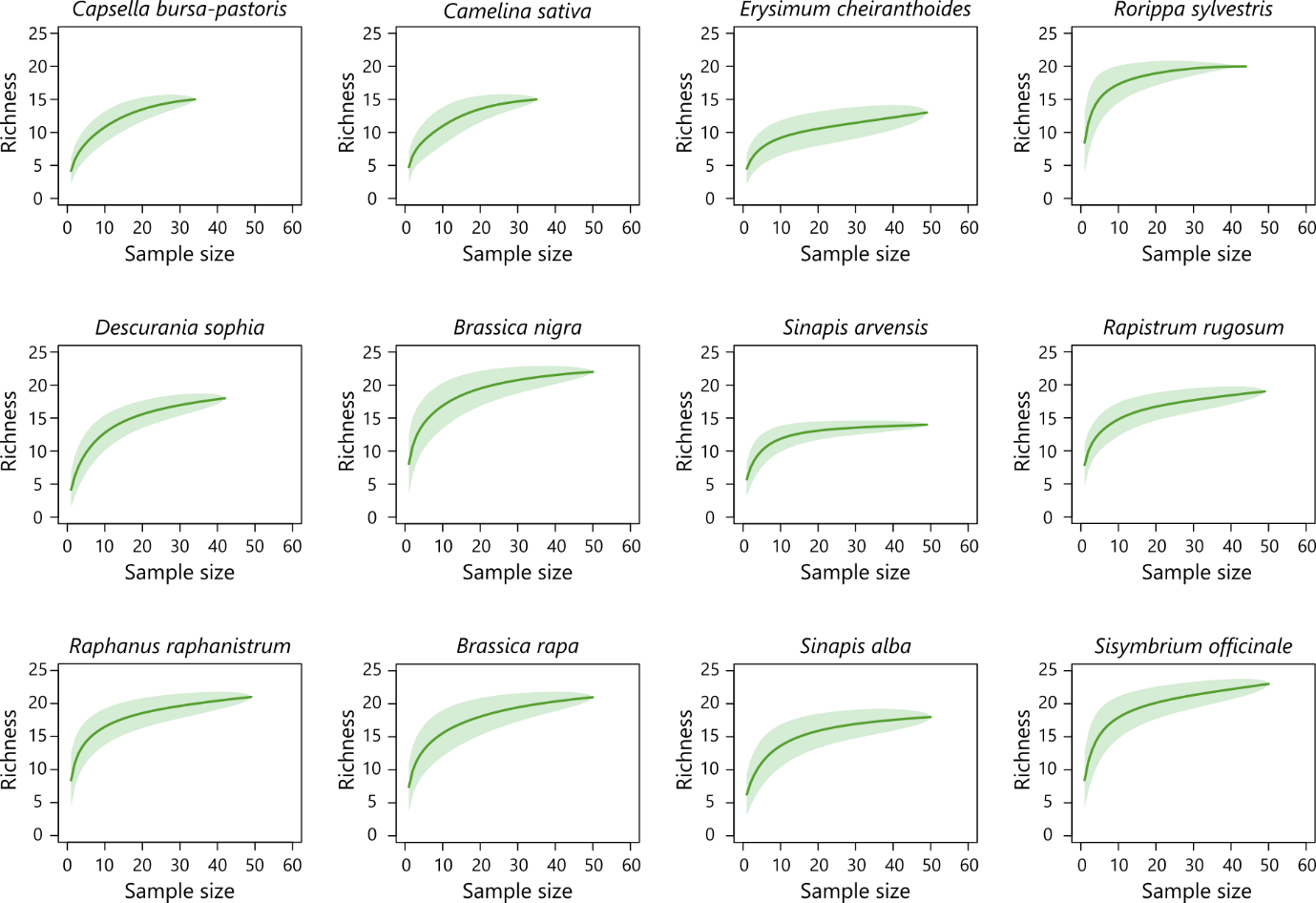


**Figure S2. Sample-based species rarefaction curves indicate high sample completeness (79.64% or higher).** Curves representing mean species richness (solid line) and variation of repeated re-sampling (shaded area). Curves reaching an asymptote are indicative of high levels of sampling completeness.


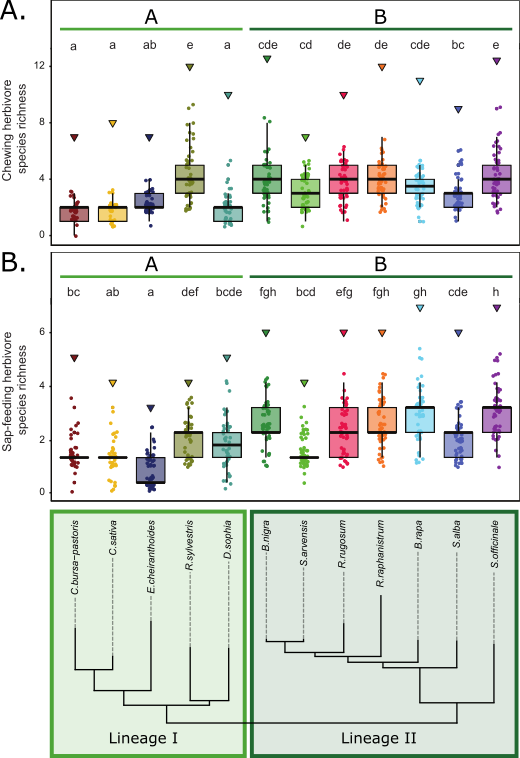


**Figure S3. Herbivore species richness is dependent on plant species and structured by plant phylogeny.** (A) Species richness of the chewing herbivore community and (B) species richness of the sap-feeding herbivore community observed across plant phylogenetic lineages (indicated in lower panel). Triangles depict the total number of herbivore species of the respective feeding guild associated with the plant species. Dots represent the number of herbivore species observed on plant individuals. Box – whisker plots summarize the variation in observed richness. Statistical analyses were performed by applying Linear Mixed Models (LMM) with species or phylogenetic lineage as explanatory factors and including plot and, when estimating the diversity for phylogenetic lineages, plant species as random factors in our models. To account for heterogeneity of variance, we allowed the variance to be different for the different species or lineages in our model. Different lowercase letters indicate significant different means (*P* < 0.05), adjusted for multiple testing by Tukey HSD. Significant differences across lineages (plant species grouped by the coloured horizontal bars) are indicated with capital letters. Statistical analyses were performed separately for the different panels.


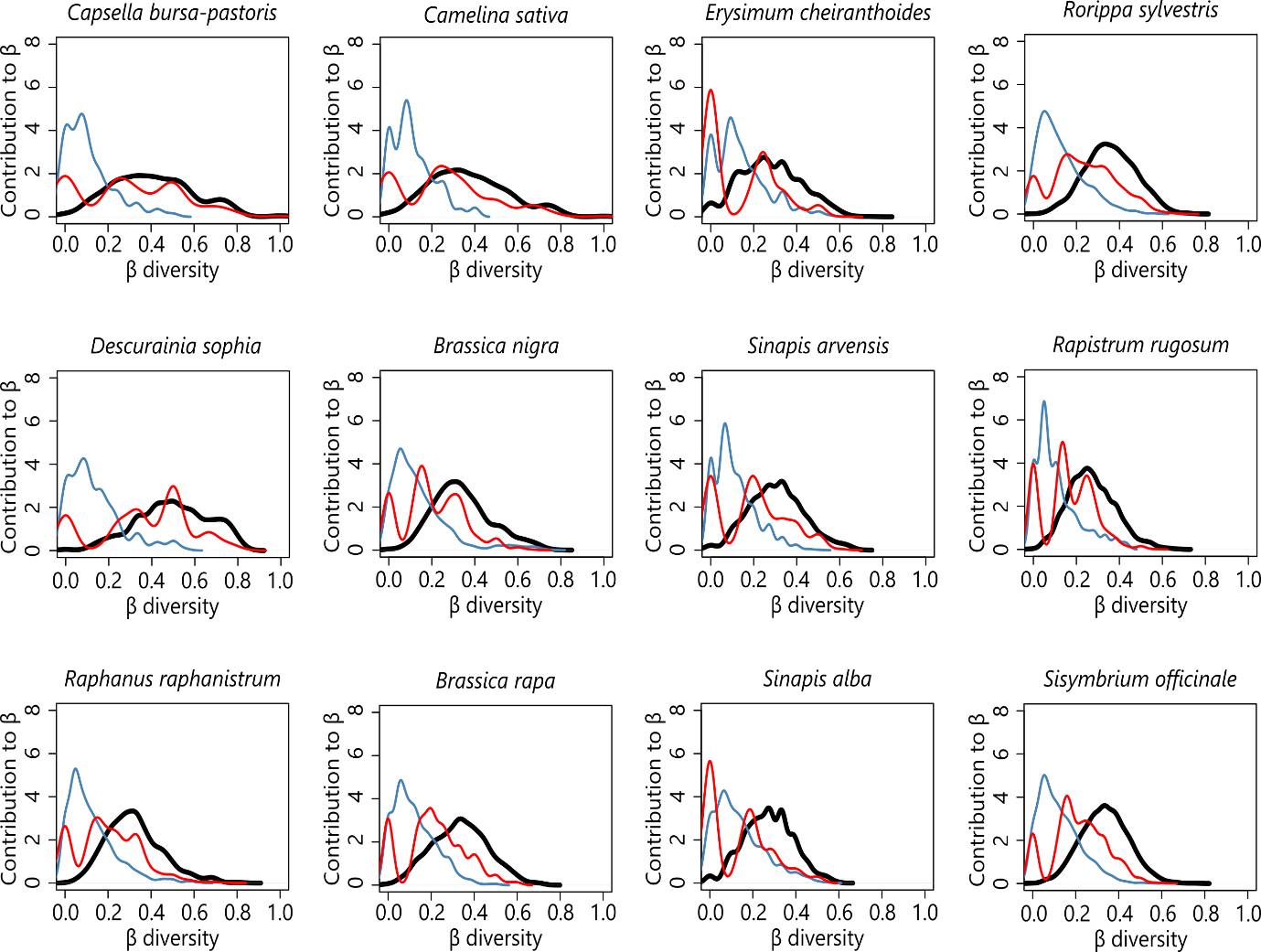


**Figure S4.** **Intraspecific variation in species composition is similar across plant species and predominantly caused by differences in herbivore species identity**. Intra-specific variation is represented by the Sørensen dissimilarity (i.e. multivariate β diversity; black line). β diversity can be partitioned in variation caused by differences in species identity (red line) and variation caused by differences in the number of species between plant individuals (blue line).


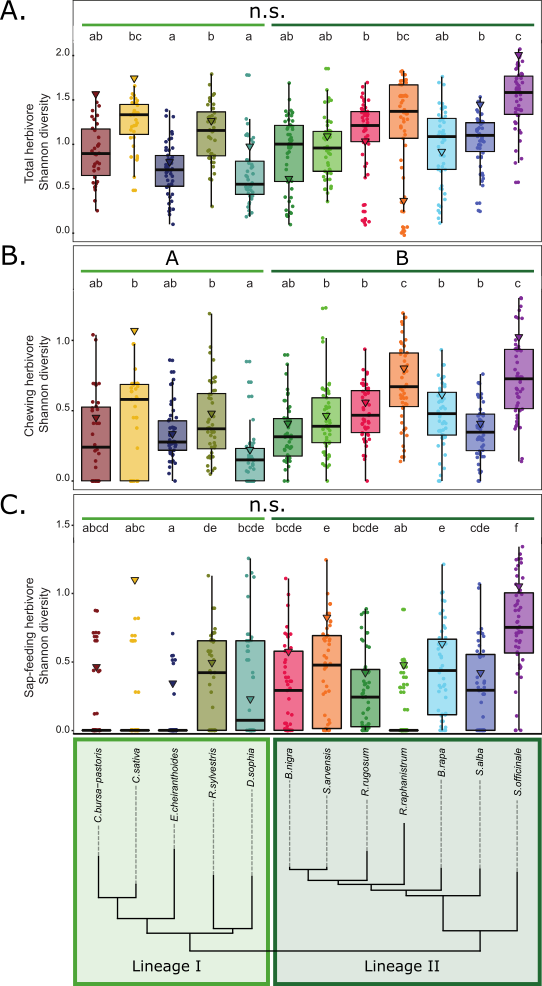


**Figure S5.** **Shannon diversity of herbivore communities is dependent on plant species and structured by plant phylogeny**. Log-based Shannon diversity of (A) the full herbivore community, (B) the chewing herbivore community, and (C) the sap-feeding herbivore community observed across plant phylogenetic lineages (indicated in lower panel). Triangles depict the Shannon diversity associated with the plant species. Dots represent the observed Shannon diversity on plant individuals. Box – whisker plots summarize the variation in Shannon diversity. Statistical analyses were performed by applying Linear Mixed Models (LMM) with species or phylogenetic lineage as explanatory factors and including plot and, when estimating the diversity for phylogenetic lineages, plant species as random factors in our models. To account for heterogeneity of variance, we allowed the variance to be different for the different species or lineages in our model. Different lowercase letters indicate significant different means (*P* < 0.05), adjusted for multiple testing by Tukey HSD. Significant differences across lineages (plant species grouped by the coloured horizontal bars) are indicated with capital letters. Statistical analyses were performed separately for the different panels.


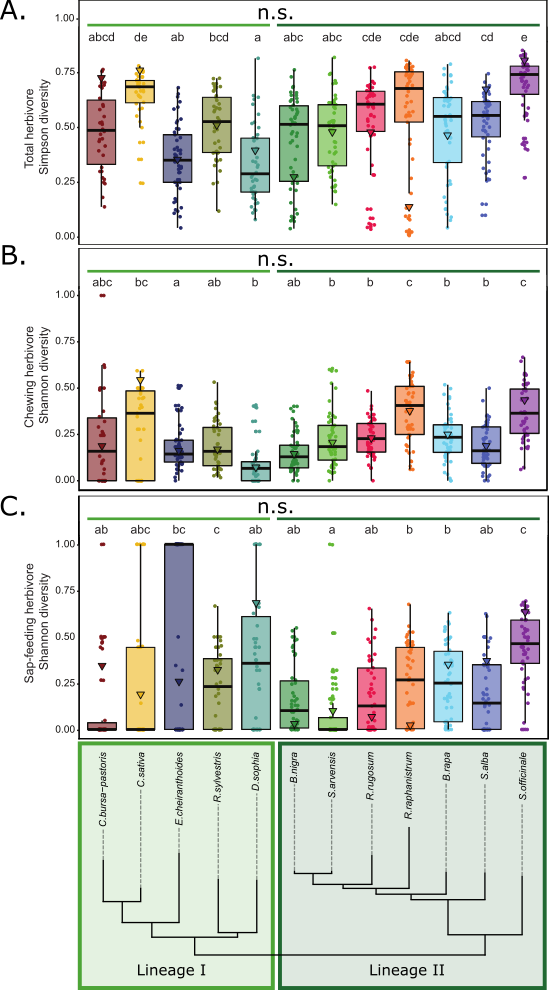


**Figure S6.** **Simpson diversity of herbivore communities is dependent on plant species and structured by plant phylogeny**. Simpson diversity of (A) the full herbivore community, (B) the chewing herbivore community, and (C) the sap-feeding herbivore community associated with plant species from two phylogenetic lineages in the Brassicaceae (lower panel). Triangles depict the Simpson diversity associated with the plant species. Dots represent the observed Simpson diversity on plant individuals. Box – whisker plots summarize the variation in Simpson diversity. Statistical analyses were performed by applying Linear Mixed Models (LMM) with species or phylogenetic lineage as explanatory factors and including plot and, when estimating the diversity for phylogenetic lineages, plant species as random factors in our models. To account for heterogeneity of variance, we allowed the variance to be different for the different species or lineages in our model. Different lowercase letters indicate significant different means (*P* < 0.05), adjusted for multiple testing by Tukey HSD. Significant differences across lineages (plant species grouped by the coloured horizontal bars) are indicated with capital letters. Statistical analyses were performed separately for the different panels.


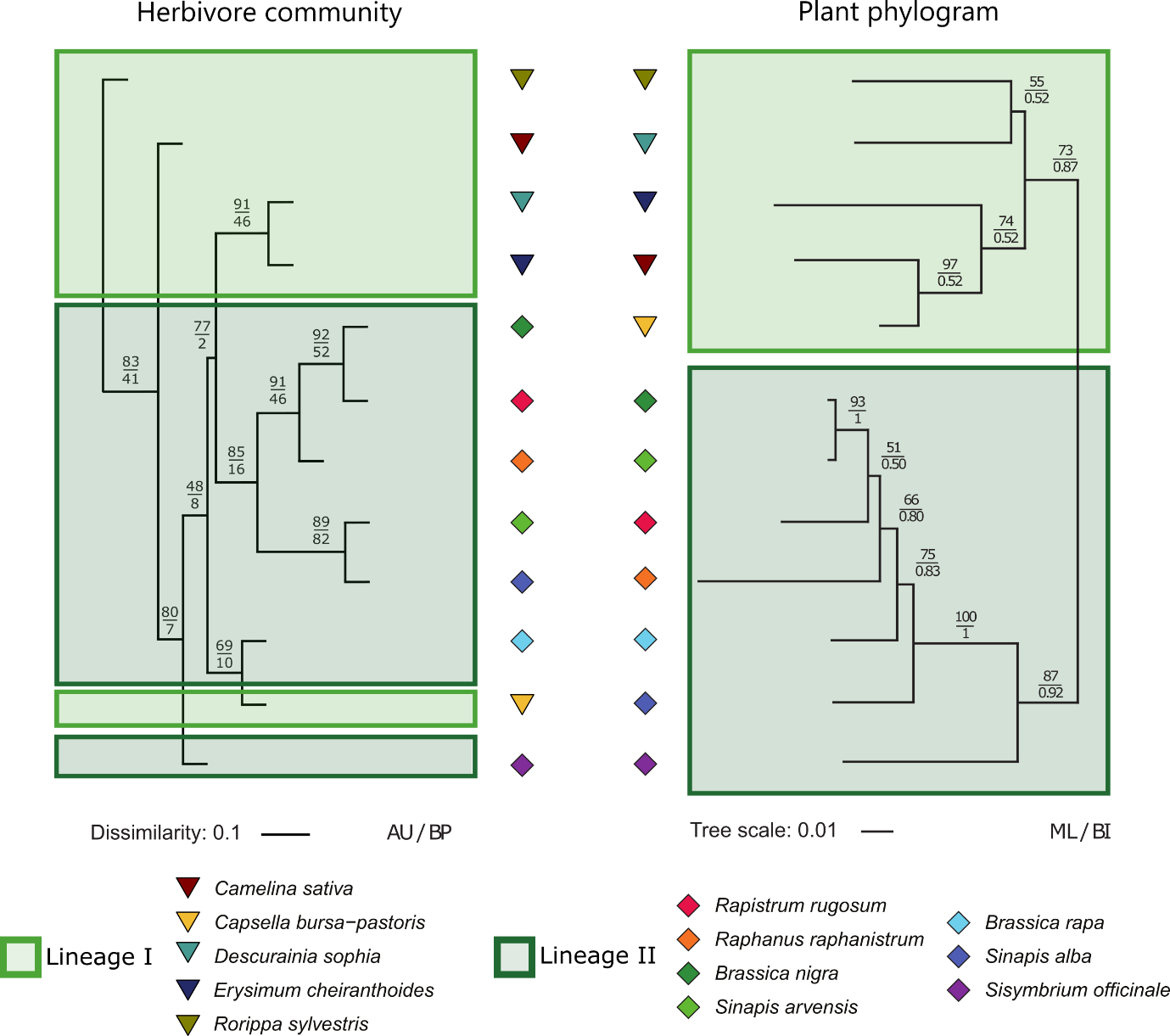


**Figure S7. Average multivariate structure of herbivore communities matches with plant phylogeny.** Comparison between cluster analysis of the centroids of the Hellinger transformed cumulative herbivore abundance data as calculated from PCA coordinates, and the Maximum Likelihood phylogram of Brassicaceae species inferred from ITS sequences. Values of Approximately Unbiased (AU) and Bootstrap Probability (BP) are displayed in the cluster analysis, and Bootstrap support values (BS) > 50% and Bayesian posterior probabilities (PP) > 0.5 are displayed in the phylogram. Scale bars indicate the Euclidean dissimilarity among centroids and the proportion of sites along each branch, respectively.

**Table S1.** Plant species used in the common garden experiment.

| **Species name** | **Common name** | **Lineage** | **N** | **Plots** |
| --- | --- | --- | --- | --- |
| *Capsella bursa-pastoris* | Sheperd's purse | I | 34 | 10 |
| *Camelina sativa* | Gold-of-pleasure | I | 35 | 10 |
| *Erysimum cheiranthoides* | Treacle-mustard | I | 49 | 10 |
| *Rorippa sylvestris* | Creeping yellowcress | I | 44 | 10 |
| *Descurainia sophia* | Flixweed | I | 42 | 10 |
| *Brassica nigra* | Black mustard | II | 50 | 10 |
| *Sinapis arvensis* | Charlock mustard | II | 49 | 10 |
| *Rapistrum rugosum* | Annual bastardcabbage | II | 49 | 10 |
| *Raphanus raphanistrum* | Wild radish | II | 49 | 10 |
| *Brassica rapa* | Field mustard | II | 50 | 10 |
| *Sinapis alba* | White mustard | II | 50 | 10 |
| *Sisymbrium officinale* | Hedge mustard | II | 50 | 10 |

**Table S2.** Herbivore insect species observed in our common garden experiment. Species annotated with an asterisk could not be identified to the species level by anatomical characteristics observed in the field and were included in our data as morphospecies.

| **Common name** |  | **Species name** | **Order** | **Family / descriptive group** | **Feeding type** |
| --- | --- | --- | --- | --- | --- |
| Red flea beetle |  | *Neocrepidodera transversa* | Coleoptera | Chrysomelidae | Chewer |
| Mustard beetle |  | *Phaedon cochleariae* | Coleoptera | Chrysomelidae | Chewer |
| Turnip flea beetle |  | *Phyllotreta atra* | Coleoptera | Chrysomelidae | Chewer |
| Small striped flea beetle |  | *Phyllotreta undulata* | Coleoptera | Chrysomelidae | Chewer |
| Pollen beetle |  | *Meligethes aeneus* | Coleoptera | Nitidulidae | Chewer |
| Harlequin bug |  | *Murgantia histrionica* | Hemiptera | Pentatomidae | Chewer |
| Turnip sawfly |  | *Athalia rosae* | Hymenoptera | Tenthredinidae | Chewer |
| Green sawfly | ***** | *Athalia* spp. | Hymenoptera | Tenthredinidae | Chewer |
| Loopers | ***** | - | Lepidoptera | Noctuidae and Geometridae | Chewer |
| Marbled yellow pearl |  | *Evergestis extimalis* | Lepidoptera | Crambidae | Chewer |
| Silver Y |  | *Autographa gamma* | Lepidoptera | Noctuidae | Chewer |
| Cabbage moth |  | *Mamestra brassicae* | Lepidoptera | Noctuidae | Chewer |
| Large cabbage white |  | *Pieris brassicae* | Lepidoptera | Pieridae | Chewer |
| Small cabbage white |  | *Pieris rapae* | Lepidoptera | Pieridae | Chewer |
| Diamondback moth |  | *Plutella xylostella* | Lepidoptera | Yponomeutidae | Chewer |
| Cicades | ***** | - | Hemiptera | Cicadellidae | Sap feeder |
| Cabbage whitefly |  | *Aleyrodes proletella* | Hemiptera | Aleyrodidae | Sap feeder |
| Black bean aphid |  | *Aphis fabae* | Hemiptera | Aphididae | Sap feeder |
| Cabbage aphid |  | *Brevicoryne brassicae* | Hemiptera | Aphididae | Sap feeder |
| Green peach aphid |  | *Myzus persicae* | Hemiptera | Aphididae | Sap feeder |
| Tobacco aphid |  | *Myzus persicae* subsp. *nicotiana* | Hemiptera | Aphididae | Sap feeder |
| Lygus bug | ***** | *Lygus* spp. | Heteroptera | Miridae | Sap feeder |
| Leaf mining flies | ***** | - | Diptera | Acromyza and Scaptomyza | Other |
| Weevils | ***** | - | Coleoptera | Curculionoidea  (mostly Ceutorhynchini) | Other |
| Gall midge | ***** | *Dasineura sisymbrii* | Diptera | Cecidomyiidae | Other |
| Thrips | ***** | - | Thysanoptera | - | Other |

**Table S3.** Summarised overview of herbivore insect species recorded over 7 years of observations on subsets of the plant species used in our experiment. This data was obtained at the same experimental site using an identical experimental methodology as the experiment presented in the main manuscript. For each plant species – year combination, we present the total number of plants in the field (N plants), the number of plants per plant species on which a specific herbivore species was observed (Prevalence), the median abundance of each herbivore species on plants it successfully colonised (Median), and the summed abundance of the herbivore on all plants of the respective plant species (Total).

| **Plant species** | **Year** | **N plants** | **Metric** | *Neocrepidodera transversa* | *Phaedon cochleariae* | *Phyllotreta atra* | *Phyllotreta undulata* | *Meligethes aeneus* | *Murgantia histrionica* | *Eurydema oleracea* | *Athalia rosae* | *Athalia* spp. | Loopers | *Evergestis extimalis* | *Evergestis forficalis* | *Autographa gamma* | *Mamestra brassicae* | *Pieris brassicae* | *Pieris rapae* | *Plutella xylostella* | *Aleyrodes proletella* | *Aphis fabae* | *Brevicoryne brassicae* | *Myzus persicae* | *Myzus persicae* subsp. *Nicotiana* | *Lipaphis erysimi* | Cicades | *Lygus* spp. | Leaf mining flies | Weevils | *Dasineura sisymbrii* | Thrips |
| --- | --- | --- | --- | --- | --- | --- | --- | --- | --- | --- | --- | --- | --- | --- | --- | --- | --- | --- | --- | --- | --- | --- | --- | --- | --- | --- | --- | --- | --- | --- | --- | --- |
| *Capsella  bursa-pastoris* | 2016 | 34 | Prevalence | 15 | 0 | 3 | 7 | 2 | 0 | 0 | 0 | 0 | 0 | 0 | 0 | 0 | 1 | 0 | 1 | 31 | 0 | 4 | 0 | 29 | 2 | 0 | 3 | 2 | 25 | 14 | 0 | 3 |
|  |  |  | Median | 1 | 0 | 1 | 1 | 2 | 0 | 0 | 0 | 0 | 0 | 0 | 0 | 0 | 1 | 0 | 1 | 6 | 0 | 14 | 0 | 8 | 1 | 0 | 1 | 2 | 3 | 1 | 0 | 1 |
|  |  |  | Total | 25 | 0 | 3 | 9 | 4 | 0 | 0 | 0 | 0 | 0 | 0 | 0 | 0 | 1 | 0 | 1 | 382 | 0 | 95 | 0 | 375 | 2 | 0 | 3 | 3 | 108 | 25 | 0 | 16 |
| *Camelina sativa* | 2016 | 35 | Prevalence | 20 | 1 | 3 | 3 | 3 | 0 | 0 | 0 | 0 | 0 | 0 | 0 | 0 | 1 | 0 | 3 | 29 | 0 | 0 | 0 | 28 | 3 | 0 | 2 | 3 | 33 | 15 | 0 | 19 |
|  |  |  | Median | 2 | 1 | 1 | 1 | 3 | 0 | 0 | 0 | 0 | 0 | 0 | 0 | 0 | 1 | 0 | 2 | 3 | 0 | 0 | 0 | 2 | 1 | 0 | 2 | 1 | 8 | 1 | 0 | 3 |
|  |  |  | Total | 55 | 1 | 3 | 3 | 8 | 0 | 0 | 0 | 0 | 0 | 0 | 0 | 0 | 1 | 0 | 6 | 122 | 0 | 0 | 0 | 88 | 3 | 0 | 3 | 4 | 265 | 21 | 0 | 55 |
| *Erysimum cheiranthoides* | 2016 | 49 | Prevalence | 48 | 1 | 0 | 9 | 1 | 0 | 0 | 0 | 0 | 0 | 0 | 0 | 0 | 1 | 0 | 6 | 49 | 0 | 0 | 0 | 21 | 1 | 0 | 0 | 6 | 43 | 10 | 0 | 25 |
|  |  |  | Median | 5 | 1 | 0 | 1 | 1 | 0 | 0 | 0 | 0 | 0 | 0 | 0 | 0 | 1 | 0 | 2 | 58 | 0 | 0 | 0 | 1 | 1 | 0 | 0 | 1 | 4 | 1 | 0 | 4 |
|  |  |  | Total | 263 | 1 | 0 | 13 | 1 | 0 | 0 | 0 | 0 | 0 | 0 | 0 | 0 | 1 | 0 | 13 | 3033 | 0 | 0 | 0 | 51 | 1 | 0 | 0 | 8 | 223 | 11 | 0 | 169 |
|  | 2017 | 23 | Prevalence | 5 | 0 | 1 | 4 | 3 | 0 | 0 | 3 | 0 | 1 | 0 | 0 | 1 | 1 | 0 | 0 | 7 | 0 | 0 | 1 | 19 | 0 | 0 | 4 | 1 | 12 | 5 | 0 | 16 |
|  |  |  | Median | 1 | 0 | 1 | 1 | 1 | 0 | 0 | 6 | 0 | 1 | 0 | 0 | 1 | 4 | 0 | 0 | 1 | 0 | 0 | 1 | 3 | 0 | 0 | 2 | 1 | 2 | 1 | 0 | 2 |
|  |  |  | Total | 8 | 0 | 1 | 5 | 4 | 0 | 0 | 20 | 0 | 1 | 0 | 0 | 1 | 4 | 0 | 0 | 10 | 0 | 0 | 1 | 80 | 0 | 0 | 6 | 1 | 31 | 8 | 0 | 60 |
|  | 2018 | 30 | Prevalence | 13 | 2 | 2 | 5 | 2 | 12 | 0 | 2 | 0 | 0 | 0 | 0 | 3 | 2 | 0 | 0 | 28 | 0 | 5 | 0 | 29 | 0 | 14 | 3 | 1 | 17 | 12 | 0 | 28 |
|  |  |  | Median | 1 | 1 | 1 | 1 | 2 | 1 | 0 | 2 | 0 | 0 | 0 | 0 | 1 | 1 | 0 | 0 | 4 | 0 | 2 | 0 | 6 | 0 | 2 | 1 | 1 | 2 | 1 | 0 | 6 |
|  |  |  | Total | 17 | 2 | 2 | 6 | 3 | 21 | 0 | 4 | 0 | 0 | 0 | 0 | 3 | 2 | 0 | 0 | 129 | 0 | 20 | 0 | 224 | 0 | 34 | 3 | 1 | 44 | 19 | 0 | 198 |
| *Rorippa sylvestris* | 2016 | 44 | Prevalence | 12 | 2 | 6 | 16 | 2 | 0 | 0 | 17 | 8 | 32 | 0 | 0 | 3 | 25 | 0 | 23 | 44 | 0 | 0 | 0 | 23 | 2 | 0 | 10 | 44 | 24 | 8 | 40 | 31 |
|  |  |  | Median | 1 | 1 | 2 | 1 | 2 | 0 | 0 | 1 | 1 | 2 | 0 | 0 | 1 | 2 | 0 | 2 | 57 | 0 | 0 | 0 | 2 | 1 | 0 | 1 | 8 | 2 | 1 | 8 | 2 |
|  |  |  | Total | 24 | 2 | 10 | 34 | 3 | 0 | 0 | 21 | 11 | 67 | 0 | 0 | 3 | 63 | 0 | 45 | 2873 | 0 | 0 | 0 | 70 | 2 | 0 | 13 | 355 | 60 | 8 | 390 | 108 |
|  | 2018 | 35 | Prevalence | 3 | 0 | 9 | 24 | 4 | 19 | 0 | 26 | 0 | 5 | 2 | 0 | 11 | 2 | 8 | 25 | 35 | 21 | 1 | 2 | 34 | 3 | 21 | 5 | 34 | 35 | 1 | 22 | 34 |
|  |  |  | Median | 1 | 0 | 1 | 2 | 3 | 2 | 0 | 2 | 0 | 1 | 2 | 0 | 1 | 1 | 80 | 4 | 10 | 2 | 1 | 1 | 13 | 1 | 2 | 1 | 4 | 6 | 1 | 4 | 7 |
|  |  |  | Total | 3 | 0 | 11 | 63 | 18 | 51 | 0 | 92 | 0 | 5 | 3 | 0 | 17 | 2 | 692 | 102 | 355 | 50 | 1 | 2 | 612 | 5 | 92 | 5 | 155 | 228 | 1 | 117 | 299 |
|  | 2017 | 31 | Prevalence | 1 | 18 | 6 | 22 | 5 | 0 | 0 | 15 | 0 | 0 | 0 | 0 | 2 | 2 | 0 | 0 | 16 | 0 | 0 | 0 | 20 | 2 | 0 | 0 | 16 | 27 | 7 | 17 | 13 |
|  |  |  | Median | 2 | 2 | 1 | 2 | 1 | 0 | 0 | 2 | 0 | 0 | 0 | 0 | 1 | 7 | 0 | 0 | 2 | 0 | 0 | 0 | 6 | 1 | 0 | 0 | 2 | 3 | 1 | 3 | 3 |
|  |  |  | Total | 2 | 64 | 8 | 43 | 8 | 0 | 0 | 37 | 0 | 0 | 0 | 0 | 2 | 14 | 0 | 0 | 35 | 0 | 0 | 0 | 183 | 2 | 0 | 0 | 38 | 94 | 9 | 81 | 50 |
| *Descurainia sophia* | 2016 | 42 | Prevalence | 11 | 0 | 2 | 1 | 1 | 0 | 0 | 1 | 4 | 0 | 0 | 0 | 6 | 10 | 0 | 4 | 42 | 0 | 0 | 6 | 26 | 2 | 0 | 7 | 23 | 19 | 3 | 0 | 6 |
|  |  |  | Median | 1 | 0 | 1 | 1 | 1 | 0 | 0 | 1 | 1 | 0 | 0 | 0 | 1 | 1 | 0 | 1 | 29 | 0 | 0 | 7 | 2 | 1 | 0 | 1 | 3 | 2 | 1 | 0 | 1 |
|  |  |  | Total | 17 | 0 | 2 | 1 | 1 | 0 | 0 | 1 | 4 | 0 | 0 | 0 | 6 | 17 | 0 | 4 | 1369 | 0 | 0 | 96 | 82 | 2 | 0 | 7 | 101 | 46 | 3 | 0 | 8 |

**Table S3. Continued.**

| **Plant species** | **Year** | **N plants** | **Metric** | *Neocrepidodera transversa* | *Phaedon cochleariae* | *Phyllotreta atra* | *Phyllotreta undulata* | *Meligethes aeneus* | *Murgantia histrionica* | *Eurydema oleracea* | *Athalia rosae* | *Athalia* spp. | Loopers | *Evergestis extimalis* | *Evergestis forficalis* | *Autographa gamma* | *Mamestra brassicae* | *Pieris brassicae* | *Pieris rapae* | *Plutella xylostella* | *Aleyrodes proletella* | *Aphis fabae* | *Brevicoryne brassicae* | *Myzus persicae* | *Myzus persicae* subsp. *Nicotiana* | *Lipaphis erysimi* | Cicades | *Lygus* spp. | Leaf mining flies | Weevils | *Dasineura sisymbrii* | Thrips |
| --- | --- | --- | --- | --- | --- | --- | --- | --- | --- | --- | --- | --- | --- | --- | --- | --- | --- | --- | --- | --- | --- | --- | --- | --- | --- | --- | --- | --- | --- | --- | --- | --- |
| *Brassica nigra* | 2011 | 225 | Prevalence | 1 | 4 | 199 | 219 | 203 | 0 | 210 | 44 | 0 | 0 | 5 | 0 | 2 | 122 | 4 | 218 | 181 | 8 | 0 | 115 | 224 | 0 | 0 | 31 | 0 | 8 | 32 | 0 | 220 |
|  |  |  | Median | 1 | 1 | 4 | 9 | 6 | 0 | 5 | 1 | 0 | 0 | 1 | 0 | 1 | 1 | 25 | 4 | 2 | 1 | 0 | 2 | 20 | 0 | 0 | 1 | 0 | 1 | 1 | 0 | 19 |
|  |  |  | Total | 1 | 4 | 971 | 2405 | 1302 | 0 | 1147 | 83 | 0 | 0 | 7 | 0 | 2 | 286 | 91 | 1137 | 443 | 9 | 0 | 547 | 5196 | 0 | 0 | 33 | 0 | 12 | 35 | 0 | 4959 |
|  | 2012 | 446 | Prevalence | 5 | 47 | 352 | 248 | 446 | 0 | 281 | 71 | 0 | 15 | 3 | 0 | 2 | 19 | 2 | 406 | 445 | 13 | 0 | 442 | 446 | 0 | 6 | 154 | 0 | 117 | 58 | 0 | 446 |
|  |  |  | Median | 1 | 2 | 2 | 2 | 150 | 0 | 2 | 1 | 0 | 1 | 2 | 0 | 1 | 1 | 14 | 4 | 9 | 1 | 0 | 248 | 32 | 0 | 2 | 1 | 0 | 2 | 1 | 0 | 31 |
|  |  |  | Total | 5 | 199 | 930 | 523 | 68411 | 0 | 588 | 95 | 0 | 18 | 5 | 0 | 2 | 56 | 28 | 1803 | 4460 | 129 | 0 | 988156 | 16498 | 0 | 39 | 223 | 0 | 277 | 66 | 0 | 14277 |
|  | 2013 | 354 | Prevalence | 16 | 173 | 32 | 93 | 281 | 31 | 266 | 21 | 0 | 57 | 4 | 97 | 43 | 18 | 13 | 167 | 353 | 5 | 194 | 354 | 249 | 122 | 221 | 125 | 0 | 346 | 112 | 0 | 354 |
|  |  |  | Median | 1 | 9 | 1 | 1 | 3 | 1 | 2 | 1 | 0 | 1 | 1 | 2 | 1 | 1 | 1 | 2 | 18 | 1 | 2 | 2266 | 3 | 1 | 2 | 1 | 0 | 9 | 1 | 0 | 14 |
|  |  |  | Total | 17 | 2739 | 35 | 112 | 1112 | 32 | 649 | 24 | 0 | 65 | 5 | 236 | 121 | 101 | 292 | 398 | 6459 | 27 | 1483 | 1438327 | 1455 | 280 | 755 | 168 | 0 | 3429 | 158 | 0 | 6220 |
|  | 2016 | 50 | Prevalence | 19 | 10 | 2 | 32 | 28 | 1 | 0 | 2 | 0 | 1 | 5 | 0 | 5 | 3 | 3 | 31 | 50 | 0 | 0 | 27 | 50 | 9 | 0 | 7 | 26 | 45 | 6 | 0 | 41 |
|  |  |  | Median | 1 | 2 | 1 | 2 | 2 | 1 | 0 | 1 | 0 | 1 | 1 | 0 | 1 | 2 | 2 | 2 | 71 | 0 | 0 | 255 | 8 | 1 | 0 | 1 | 3 | 5 | 1 | 0 | 16 |
|  |  |  | Total | 30 | 24 | 2 | 75 | 96 | 1 | 0 | 2 | 0 | 1 | 9 | 0 | 7 | 7 | 5 | 67 | 3939 | 0 | 0 | 34977 | 444 | 9 | 0 | 9 | 105 | 279 | 8 | 0 | 1108 |
|  | 2017 | 109 | Prevalence | 31 | 6 | 24 | 104 | 75 | 2 | 0 | 17 | 0 | 4 | 33 | 1 | 2 | 4 | 1 | 42 | 76 | 0 | 2 | 53 | 109 | 8 | 12 | 37 | 40 | 76 | 30 | 0 | 109 |
|  |  |  | Median | 1 | 1 | 1 | 4 | 2 | 1 | 0 | 1 | 0 | 1 | 1 | 1 | 2 | 1 | 35 | 4 | 2 | 0 | 2 | 17 | 16 | 1 | 4 | 1 | 1 | 3 | 1 | 0 | 10 |
|  |  |  | Total | 44 | 14 | 34 | 549 | 257 | 2 | 0 | 32 | 0 | 4 | 52 | 1 | 3 | 4 | 35 | 207 | 174 | 0 | 3 | 2612 | 1970 | 15 | 60 | 48 | 75 | 249 | 40 | 0 | 2386 |
|  | 2018 | 120 | Prevalence | 36 | 6 | 31 | 114 | 101 | 37 | 0 | 96 | 1 | 7 | 6 | 0 | 23 | 2 | 12 | 84 | 120 | 2 | 25 | 81 | 120 | 13 | 102 | 18 | 27 | 112 | 35 | 0 | 119 |
|  |  |  | Median | 1 | 2 | 1 | 4 | 4 | 3 | 0 | 3 | 1 | 1 | 1 | 0 | 1 | 2 | 34 | 4 | 20 | 1 | 1 | 45 | 44 | 1 | 4 | 1 | 1 | 4 | 1 | 0 | 12 |
|  |  |  | Total | 50 | 18 | 37 | 598 | 490 | 117 | 0 | 308 | 1 | 7 | 11 | 0 | 34 | 3 | 460 | 362 | 2456 | 2 | 43 | 15763 | 5820 | 30 | 1111 | 23 | 32 | 479 | 51 | 0 | 2370 |
| *Sinapis arvensis* | 2016 | 49 | Prevalence | 24 | 1 | 11 | 7 | 45 | 0 | 0 | 0 | 0 | 0 | 0 | 0 | 0 | 0 | 0 | 15 | 49 | 0 | 0 | 0 | 47 | 6 | 0 | 5 | 3 | 39 | 11 | 0 | 18 |
|  |  |  | Median | 2 | 1 | 1 | 1 | 4 | 0 | 0 | 0 | 0 | 0 | 0 | 0 | 0 | 0 | 0 | 2 | 58 | 0 | 0 | 0 | 8 | 1 | 0 | 1 | 1 | 5 | 1 | 0 | 4 |
|  |  |  | Total | 60 | 1 | 20 | 10 | 261 | 0 | 0 | 0 | 0 | 0 | 0 | 0 | 0 | 0 | 0 | 23 | 2932 | 0 | 0 | 0 | 432 | 14 | 0 | 7 | 3 | 267 | 13 | 0 | 79 |
|  | 2017 | 86 | Prevalence | 23 | 8 | 21 | 54 | 83 | 1 | 0 | 16 | 0 | 0 | 0 | 0 | 2 | 12 | 0 | 38 | 40 | 0 | 0 | 11 | 84 | 11 | 3 | 15 | 29 | 74 | 33 | 0 | 83 |
|  |  |  | Median | 1 | 1 | 1 | 2 | 7 | 2 | 0 | 1 | 0 | 0 | 0 | 0 | 4 | 1 | 0 | 6 | 1 | 0 | 0 | 10 | 13 | 1 | 3 | 1 | 1 | 5 | 1 | 0 | 7 |
|  |  |  | Total | 40 | 11 | 23 | 114 | 659 | 2 | 0 | 20 | 0 | 0 | 0 | 0 | 9 | 113 | 0 | 204 | 63 | 0 | 0 | 519 | 1472 | 23 | 11 | 19 | 46 | 383 | 53 | 0 | 680 |
|  | 2018 | 120 | Prevalence | 28 | 5 | 6 | 38 | 118 | 3 | 0 | 73 | 0 | 0 | 0 | 0 | 18 | 0 | 2 | 59 | 116 | 0 | 13 | 6 | 120 | 7 | 56 | 16 | 14 | 116 | 29 | 0 | 111 |
|  |  |  | Median | 1 | 3 | 1 | 1 | 16 | 1 | 0 | 2 | 0 | 0 | 0 | 0 | 1 | 0 | 1 | 4 | 6 | 0 | 1 | 15 | 34 | 4 | 2 | 1 | 1 | 5 | 1 | 0 | 6 |
|  |  |  | Total | 37 | 30 | 6 | 47 | 2099 | 4 | 0 | 157 | 0 | 0 | 0 | 0 | 27 | 0 | 2 | 226 | 770 | 0 | 18 | 147 | 4351 | 28 | 190 | 18 | 14 | 637 | 39 | 0 | 693 |

**Table S3. Continued.**

| **Plant species** | **Year** | **N plants** | **Metric** | *Neocrepidodera transversa* | *Phaedon cochleariae* | *Phyllotreta atra* | *Phyllotreta undulata* | *Meligethes aeneus* | *Murgantia histrionica* | *Eurydema oleracea* | *Athalia rosae* | *Athalia* spp. | Loopers | *Evergestis extimalis* | *Evergestis forficalis* | *Autographa gamma* | *Mamestra brassicae* | *Pieris brassicae* | *Pieris rapae* | *Plutella xylostella* | *Aleyrodes proletella* | *Aphis fabae* | *Brevicoryne brassicae* | *Myzus persicae* | *Myzus persicae* subsp. *Nicotiana* | *Lipaphis erysimi* | Cicades | *Lygus* spp. | Leaf mining flies | Weevils | *Dasineura sisymbrii* | Thrips |
| --- | --- | --- | --- | --- | --- | --- | --- | --- | --- | --- | --- | --- | --- | --- | --- | --- | --- | --- | --- | --- | --- | --- | --- | --- | --- | --- | --- | --- | --- | --- | --- | --- |
| *Rapistrum rugosum* | 2016 | 49 | Prevalence | 23 | 1 | 5 | 27 | 37 | 0 | 0 | 0 | 0 | 0 | 1 | 0 | 5 | 2 | 0 | 35 | 49 | 1 | 0 | 11 | 49 | 6 | 0 | 7 | 26 | 47 | 8 | 0 | 46 |
|  |  |  | Median | 1 | 2 | 1 | 1 | 3 | 0 | 0 | 0 | 0 | 0 | 1 | 0 | 1 | 1 | 0 | 2 | 48 | 1 | 0 | 400 | 7 | 1 | 0 | 1 | 3 | 6 | 1 | 0 | 23 |
|  |  |  | Total | 40 | 2 | 6 | 62 | 152 | 0 | 0 | 0 | 0 | 0 | 1 | 0 | 5 | 2 | 0 | 97 | 2561 | 1 | 0 | 11995 | 325 | 6 | 0 | 9 | 101 | 314 | 13 | 0 | 1318 |
|  | 2017 | 113 | Prevalence | 30 | 4 | 28 | 94 | 74 | 2 | 0 | 67 | 0 | 6 | 14 | 0 | 6 | 5 | 1 | 42 | 70 | 0 | 0 | 8 | 113 | 22 | 6 | 18 | 38 | 81 | 32 | 0 | 104 |
|  |  |  | Median | 1 | 1 | 1 | 3 | 2 | 6 | 0 | 2 | 0 | 1 | 1 | 0 | 1 | 1 | 53 | 4 | 2 | 0 | 0 | 6 | 25 | 2 | 1 | 1 | 1 | 3 | 1 | 0 | 6 |
|  |  |  | Total | 54 | 7 | 39 | 336 | 251 | 13 | 0 | 182 | 0 | 6 | 24 | 0 | 14 | 5 | 53 | 214 | 156 | 0 | 0 | 100 | 3701 | 52 | 9 | 21 | 63 | 294 | 51 | 0 | 1079 |
|  | 2018 | 114 | Prevalence | 28 | 1 | 18 | 83 | 97 | 47 | 1 | 83 | 0 | 5 | 2 | 1 | 31 | 2 | 20 | 83 | 113 | 1 | 18 | 29 | 114 | 13 | 93 | 14 | 20 | 111 | 56 | 0 | 109 |
|  |  |  | Median | 1 | 1 | 1 | 3 | 6 | 2 | 1 | 2 | 0 | 1 | 1 | 1 | 1 | 1 | 38 | 4 | 11 | 1 | 1 | 6 | 50 | 1 | 5 | 1 | 1 | 8 | 2 | 0 | 14 |
|  |  |  | Total | 34 | 1 | 23 | 321 | 728 | 159 | 1 | 238 | 0 | 5 | 2 | 1 | 47 | 2 | 1211 | 476 | 1382 | 1 | 33 | 533 | 6079 | 15 | 712 | 17 | 27 | 936 | 108 | 0 | 2487 |
| *Raphanus raphanistrum* | 2016 | 49 | Prevalence | 28 | 6 | 5 | 23 | 44 | 2 | 0 | 0 | 1 | 1 | 0 | 0 | 7 | 3 | 1 | 24 | 49 | 0 | 0 | 16 | 47 | 7 | 0 | 9 | 36 | 39 | 20 | 0 | 42 |
|  |  |  | Median | 2 | 1 | 2 | 2 | 8 | 4 | 0 | 0 | 1 | 1 | 0 | 0 | 1 | 1 | 1 | 2 | 46 | 0 | 0 | 350 | 9 | 1 | 0 | 1 | 8 | 4 | 1 | 0 | 18 |
|  |  |  | Total | 87 | 8 | 11 | 58 | 472 | 8 | 0 | 0 | 1 | 1 | 0 | 0 | 7 | 5 | 1 | 71 | 2471 | 0 | 0 | 71710 | 454 | 22 | 0 | 11 | 377 | 198 | 29 | 0 | 1069 |
|  | 2017 | 117 | Prevalence | 30 | 5 | 32 | 92 | 115 | 0 | 0 | 12 | 0 | 6 | 0 | 1 | 6 | 5 | 0 | 43 | 65 | 0 | 5 | 25 | 117 | 21 | 20 | 22 | 68 | 104 | 67 | 0 | 112 |
|  |  |  | Median | 1 | 1 | 1 | 3 | 8 | 0 | 0 | 1 | 0 | 1 | 0 | 1 | 1 | 1 | 0 | 4 | 2 | 0 | 1 | 20 | 13 | 1 | 7 | 1 | 3 | 5 | 1 | 0 | 11 |
|  |  |  | Total | 38 | 5 | 40 | 320 | 1051 | 0 | 0 | 19 | 0 | 7 | 0 | 1 | 6 | 5 | 0 | 198 | 114 | 0 | 16 | 971 | 2010 | 56 | 268 | 28 | 224 | 583 | 125 | 0 | 1856 |
|  | 2018 | 120 | Prevalence | 56 | 9 | 22 | 76 | 120 | 15 | 1 | 58 | 0 | 2 | 5 | 0 | 26 | 2 | 14 | 72 | 120 | 0 | 51 | 65 | 120 | 7 | 109 | 13 | 34 | 120 | 95 | 0 | 120 |
|  |  |  | Median | 1 | 2 | 1 | 2 | 28 | 1 | 19 | 2 | 0 | 1 | 1 | 0 | 1 | 1 | 44 | 4 | 12 | 0 | 4 | 15 | 42 | 4 | 10 | 1 | 1 | 9 | 2 | 0 | 10 |
|  |  |  | Total | 95 | 30 | 26 | 209 | 3678 | 21 | 19 | 148 | 0 | 2 | 5 | 0 | 68 | 2 | 810 | 261 | 1606 | 0 | 407 | 4557 | 5598 | 29 | 1489 | 15 | 46 | 1113 | 255 | 0 | 1639 |
| *Brassica rapa* | 2016 | 50 | Prevalence | 26 | 4 | 8 | 41 | 1 | 3 | 0 | 0 | 1 | 0 | 1 | 0 | 3 | 3 | 0 | 31 | 50 | 2 | 0 | 35 | 50 | 11 | 0 | 6 | 27 | 40 | 12 | 0 | 15 |
|  |  |  | Median | 1 | 1 | 2 | 2 | 2 | 2 | 0 | 0 | 1 | 0 | 1 | 0 | 1 | 1 | 0 | 1 | 36 | 1 | 0 | 80 | 14 | 2 | 0 | 1 | 3 | 6 | 1 | 0 | 2 |
|  |  |  | Total | 47 | 4 | 12 | 128 | 2 | 5 | 0 | 0 | 1 | 0 | 1 | 0 | 3 | 66 | 0 | 66 | 2079 | 2 | 0 | 21157 | 5871 | 61 | 0 | 7 | 160 | 248 | 12 | 0 | 74 |
| *Sinapis alba* | 2016 | 50 | Prevalence | 7 | 3 | 4 | 10 | 46 | 0 | 0 | 5 | 0 | 0 | 0 | 0 | 0 | 0 | 2 | 16 | 50 | 0 | 1 | 1 | 50 | 16 | 0 | 7 | 12 | 50 | 4 | 0 | 29 |
|  |  |  | Median | 1 | 1 | 1 | 1 | 6 | 0 | 0 | 2 | 0 | 0 | 0 | 0 | 0 | 0 | 3 | 2 | 60 | 0 | 16 | 2150 | 10 | 2 | 0 | 1 | 2 | 11 | 1 | 0 | 3 |
|  |  |  | Total | 11 | 11 | 4 | 10 | 380 | 0 | 0 | 8 | 0 | 0 | 0 | 0 | 0 | 0 | 6 | 26 | 3963 | 0 | 16 | 2150 | 550 | 43 | 0 | 18 | 23 | 552 | 4 | 0 | 215 |
| *Sisymbrium officinale* | 2016 | 50 | Prevalence | 26 | 1 | 12 | 25 | 0 | 8 | 0 | 6 | 7 | 5 | 1 | 0 | 14 | 18 | 3 | 45 | 50 | 0 | 1 | 19 | 48 | 1 | 0 | 21 | 46 | 45 | 4 | 0 | 16 |
|  |  |  | Median | 2 | 1 | 1 | 2 | 0 | 2 | 0 | 1 | 1 | 1 | 1 | 0 | 1 | 1 | 1 | 3 | 27 | 0 | 1 | 12 | 8 | 1 | 0 | 2 | 8 | 7 | 1 | 0 | 8 |
|  |  |  | Total | 52 | 1 | 13 | 64 | 0 | 17 | 0 | 9 | 11 | 8 | 1 | 0 | 25 | 142 | 4 | 176 | 1445 | 0 | 1 | 279 | 799 | 1 | 0 | 39 | 457 | 310 | 4 | 0 | 148 |

**Table S4.** Overview of the herbivore community matrices used in our analyses, presenting the biological level and interpretation of values in the matrix.

|  | **Biological level** | **Values represent** | **Standardisation** | **Transformation** | **Used in analysis** |
| --- | --- | --- | --- | --- | --- |
| **A.** | Plant individual | Cumulative abundance of herbivores | - | - | Univariate analysis of Shannon and Simpson indices |
| **B.** | Plant individual | Incidence of herbivores | - | - | Univariate analysis of species richness,  Uni- and Multivariate analysis β diversity,  Multivariate analysis of community composition,  SIMPER analysis |
| **C.** | Plant individual | Cumulative abundance of herbivores | - | log (x + 1) | Multivariate analysis of community structure,  Multivariate regression of plant traits. |
| **D.** | Plant species | Cumulative abundance of herbivores | - | - | Network analysis |
| **E.** | Plant species | Percentage of plants | Proportion | - | Network analysis |

The proportion standardisation (matrix E) was calculated by summing the number of plants belonging to a specific plant species on which the herbivore species occurred and dividing this number by the total number of plants belonging to this species in our experiment. We chose not to standardize the abundance-based associational matrix D as the relation between the summed abundance of herbivores and plant sample size is likely to be herbivore-species-specific due to differences in the biology of the herbivores.

**Table S5.** Diversity measures of herbivore communities. Interpolated diversity is estimated using sample-based rarefaction and presented by the estimated means and SD. Extrapolated species richness is calculated by Chao’s equation (mean ± SE) corrected for small sample sizes.

|  |  |  | *Capsella*  *bursa-pastoris* | | | *Camelina*  *sativa* | | | *Erysimum cheiranthoides* | | | *Rorippa*  *sylvestris* | | | *Descurainia*  *sophia* | | | | *Brassica*  *nigra* | | | |
| --- | --- | --- | --- | --- | --- | --- | --- | --- | --- | --- | --- | --- | --- | --- | --- | --- | --- | --- | --- | --- | --- | --- |
| **Full**  **herbivore community** | Richness | Interpolated | 15.00 | ± | 0.00 | 14.81 | ± | 0.40 | 11.53 | ± | 1.18 | 19.73 | ± | 0.50 | | 16.94 | ± | 0.82 | 20.85 | ± | 0.98 |  |
|  |  | Observed | 15.00 |  |  | 15.00 |  |  | 13.00 |  |  | 20.00 |  |  | | 18.00 |  |  | 22.00 |  |  |  |
|  |  | Extrapolated | 16.45 | ± | 2.23 | 15.97 | ± | 1.82 | 18.88 | ± | 7.05 | 20.00 | ± | 0.00 | | 25.80 | ± | 11.38 | 22.98 | ± | 1.84 |  |
|  |  | Completeness | 91.18 | % |  | 93.92 | % |  | 68.86 | % |  | 100.00 | % |  | | 69.75 | % |  | 95.74 | % |  |  |
|  | Shannon | Interpolated | 1.56 | ± | 0.02 | 1.74 | ± | 0.02 | 0.80 | ± | 0.04 | 1.26 | ± | 0.04 | | 0.96 | ± | 0.07 | 0.63 | ± | 0.12 |  |
|  |  | Observed | 1.56 |  |  | 1.75 |  |  | 0.80 |  |  | 1.26 |  |  | | 0.97 |  |  | 0.62 |  |  |  |
|  | Exponential Shannon entropy | Interpolated | 4.77 | ± | 0.11 | 5.75 | ± | 0.12 | 2.23 | ± | 0.10 | 3.55 | ± | 0.15 | | 2.62 | ± | 0.17 | 1.90 | ± | 0.26 |  |
|  |  | Observed | 4.78 |  |  | 5.76 |  |  | 2.24 |  |  | 3.54 |  |  | | 2.63 |  |  | 1.85 |  |  |  |
|  | Simpson | Interpolated | 0.72 | ± | 0.01 | 0.76 | ± | 0.01 | 0.35 | ± | 0.02 | 0.50 | ± | 0.02 | | 0.39 | ± | 0.03 | 0.28 | ± | 0.06 |  |
|  |  | Observed | 0.72 |  |  | 0.76 |  |  | 0.35 |  |  | 0.51 |  |  | | 0.39 |  |  | 0.27 |  |  |  |
| **Chewing**  **herbivore community** | Richness | Interpolated | 7.00 | ± | 0.00 | 7.82 | ± | 0.40 | 5.90 | ± | 0.83 | 11.81 | ± | 0.43 | | 9.01 | ± | 0.82 | 12.23 | ± | 0.72 |  |
|  |  | Observed | 7.00 |  |  | 8.00 |  |  | 7.00 |  |  | 12.00 |  |  | | 10.00 |  |  | 13.00 |  |  |  |
|  |  | Extrapolated | 11.35 | ± | 6.97 | 9.94 | ± | 3.64 | 9.94 | ± | 4.36 | 12.00 | ± | 0.00 | | 15.85 | ± | 6.97 | 13.25 | ± | 0.72 |  |
|  |  | Completeness | 61.65 | % |  | 80.47 | % |  | 70.43 | % |  | 100.00 | % |  | | 63.08 | % |  | 98.15 | % |  |  |
|  | Shannon | Interpolated | 0.43 | ± | 0.02 | 1.05 | ± | 0.04 | 0.33 | ± | 0.02 | 0.44 | ± | 0.03 | | 0.17 | ± | 0.02 | 0.39 | ± | 0.03 |  |
|  |  | Observed | 0.43 |  |  | 1.05 |  |  | 0.34 |  |  | 0.48 |  |  | | 0.20 |  |  | 0.41 |  |  |  |
|  | Exponential Shannon entropy | Interpolated | 1.53 | ± | 0.03 | 2.86 | ± | 0.11 | 1.40 | ± | 0.03 | 1.55 | ± | 0.05 | | 1.19 | ± | 0.02 | 1.48 | ± | 0.04 |  |
|  |  | Observed | 1.53 |  |  | 2.86 |  |  | 1.40 |  |  | 1.62 |  |  | | 1.23 |  |  | 1.51 |  |  |  |
|  | Simpson | Interpolated | 0.18 | ± | 0.01 | 0.54 | ± | 0.02 | 0.16 | ± | 0.01 | 0.16 | ± | 0.01 | | 0.06 | ± | 0.01 | 0.14 | ± | 0.01 |  |
|  |  | Observed | 0.18 |  |  | 0.54 |  |  | 0.16 |  |  | 0.17 |  |  | | 0.07 |  |  | 0.15 |  |  |  |
| **Sap-feeding**  **herbivore community** | Richness | Interpolated | 5.00 | ± | 0.00 | 3.99 | ± | 0.08 | 2.63 | ± | 0.48 | 3.92 | ± | 0.28 | | 4.95 | ± | 0.23 | 5.62 | ± | 0.48 |  |
|  |  | Observed | 5.00 |  |  | 4.00 |  |  | 3.00 |  |  | 4.00 |  |  | | 5.00 |  |  | 6.00 |  |  |  |
|  |  | Extrapolated | 5.00 | ± | 0.00 | 4.00 | ± | 0.00 | 3.00 | ± | 0.40 | 4.00 | ± | 0.00 | | 5.00 | ± | 0.00 | 6.00 | ± | 0.45 |  |
|  |  | Completeness | 100.00 | % |  | 100.00 | % |  | 100.00 | % |  | 100.00 | % |  | | 100.00 | % |  | 100.00 | % |  |  |
|  | Shannon | Interpolated | 0.13 | ± | 0.01 | 0.44 | ± | 0.05 | 0.46 | ± | 0.07 | 0.59 | ± | 0.05 | | 0.87 | ± | 0.03 | 0.64 | ± | 0.05 |  |
|  |  | Observed | 0.60 |  |  | 0.44 |  |  | 0.48 |  |  | 0.59 |  |  | | 1.22 |  |  | 0.09 |  |  |  |
|  | Exponential Shannon entropy | Interpolated | 1.13 | ± | 0.01 | 1.55 | ± | 0.07 | 1.59 | ± | 0.10 | 1.81 | ± | 0.09 | | 2.38 | ± | 0.08 | 1.90 | ± | 0.10 |  |
|  |  | Observed | 1.83 |  |  | 1.55 |  |  | 1.61 |  |  | 1.81 |  |  | | 3.38 |  |  | 1.10 |  |  |  |
|  | Simpson | Interpolated | 0.04 | ± | 0.00 | 0.19 | ± | 0.02 | 0.26 | ± | 0.05 | 0.32 | ± | 0.04 | | 0.54 | ± | 0.01 | 0.35 | ± | 0.04 |  |
|  |  | Observed | 0.35 |  |  | 0.19 |  |  | 0.26 |  |  | 0.32 |  |  | | 0.68 |  |  | 0.03 |  |  |  |

**Table S5. Continued.**

|  |  |  | *Sinapis*  *arvensis* | | | *Rapistrum*  *rugosum* | | | *Raphanus raphanistrum* | | | *Brassica*  *rapa* | | | *Sinapis*  *alba* | | | | *Sisymbrium*  *officinale* | | | |
| --- | --- | --- | --- | --- | --- | --- | --- | --- | --- | --- | --- | --- | --- | --- | --- | --- | --- | --- | --- | --- | --- | --- |
| **Full**  **herbivore community** | Richness | Interpolated | 13.58 | ± | 0.52 | 17.76 | ± | 0.88 | 19.72 | ± | 0.91 | 19.55 | ± | 1.04 | | 17.01 | ± | 1.06 | 21.41 | ± | 0.98 |  |
|  |  | Observed | 14.00 |  |  | 19.00 |  |  | 21.00 |  |  | 21.00 |  |  | | 18.00 |  |  | 23.00 |  |  |  |
|  |  | Extrapolated | 14.00 | ± | 0.47 | 23.41 | ± | 7.05 | 25.41 | ± | 7.05 | 25.41 | ± | 7.06 | | 19.96 | ± | 3.67 | 28.88 | ± | 7.13 |  |
|  |  | Completeness | 100.00 | % |  | 81.17 | % |  | 82.65 | % |  | 82.64 | % |  | | 90.18 | % |  | 79.64 | % |  |  |
|  | Shannon | Interpolated | 1.09 | ± | 0.05 | 1.06 | ± | 0.18 | 0.41 | ± | 0.19 | 0.91 | ± | 0.10 | | 1.31 | ± | 0.12 | 1.99 | ± | 0.05 |  |
|  |  | Observed | 1.09 |  |  | 1.04 |  |  | 0.37 |  |  | 0.92 |  |  | | 1.45 |  |  | 2.00 |  |  |  |
|  | Exponential Shannon entropy | Interpolated | 2.98 | ± | 0.14 | 2.93 | ± | 0.59 | 1.54 | ± | 0.44 | 2.50 | ± | 0.26 | | 3.72 | ± | 0.42 | 7.29 | ± | 0.37 |  |
|  |  | Observed | 2.98 |  |  | 2.82 |  |  | 1.45 |  |  | 2.51 |  |  | | 4.26 |  |  | 7.42 |  |  |  |
|  | Simpson | Interpolated | 0.47 | ± | 0.02 | 0.48 | ± | 0.09 | 0.15 | ± | 0.08 | 0.46 | ± | 0.07 | | 0.61 | ± | 0.08 | 0.80 | ± | 0.01 |  |
|  |  | Observed | 0.47 |  |  | 0.47 |  |  | 0.13 |  |  | 0.46 |  |  | | 0.67 |  |  | 0.80 |  |  |  |
| **Chewing**  **herbivore community** | Richness | Interpolated | 6.63 | ± | 0.49 | 9.13 | ± | 0.74 | 10.85 | ± | 0.82 | 9.74 | ± | 0.94 | | 8.79 | ± | 0.49 | 12.18 | ± | 0.73 |  |
|  |  | Observed | 7.00 |  |  | 10.00 |  |  | 12.00 |  |  | 11.00 |  |  | | 9.00 |  |  | 13.00 |  |  |  |
|  |  | Extrapolated | 7.00 | ± | 0.45 | 11.96 | ± | 3.67 | 14.94 | ± | 4.43 | 13.94 | ± | 4.42 | | 9.00 | ± | 0.00 | 13.98 | ± | 2.24 |  |
|  |  | Completeness | 100.00 | % |  | 83.62 | % |  | 80.33 | % |  | 78.91 | % |  | | 100.00 | % |  | 92.99 | % |  |  |
|  | Shannon | Interpolated | 0.46 | ± | 0.03 | 0.55 | ± | 0.02 | 0.78 | ± | 0.04 | 0.59 | ± | 0.08 | | 0.40 | ± | 0.02 | 0.92 | ± | 0.10 |  |
|  |  | Observed | 0.47 |  |  | 0.56 |  |  | 0.80 |  |  | 0.61 |  |  | | 0.41 |  |  | 1.03 |  |  |  |
|  | Exponential Shannon entropy | Interpolated | 1.59 | ± | 0.05 | 1.73 | ± | 0.04 | 2.19 | ± | 0.08 | 1.82 | ± | 0.15 | | 1.49 | ± | 0.03 | 2.52 | ± | 0.25 |  |
|  |  | Observed | 1.59 |  |  | 1.75 |  |  | 2.23 |  |  | 1.85 |  |  | | 1.51 |  |  | 2.79 |  |  |  |
|  | Simpson | Interpolated | 0.21 | ± | 0.02 | 0.23 | ± | 0.01 | 0.37 | ± | 0.02 | 0.25 | ± | 0.04 | | 0.19 | ± | 0.01 | 0.41 | ± | 0.06 |  |
|  |  | Observed | 0.21 |  |  | 0.23 |  |  | 0.38 |  |  | 0.25 |  |  | | 0.19 |  |  | 0.44 |  |  |  |
| **Sap-feeding**  **herbivore community** | Richness | Interpolated | 3.95 | ± | 0.22 | 5.63 | ± | 0.48 | 5.87 | ± | 0.33 | 6.81 | ± | 0.47 | | 5.24 | ± | 0.97 | 6.24 | ± | 0.68 |  |
|  |  | Observed | 4.00 |  |  | 6.00 |  |  | 6.00 |  |  | 7.00 |  |  | | 6.00 |  |  | 7.00 |  |  |  |
|  |  | Extrapolated | 4.00 | ± | 0.00 | 6.00 | ± | 0.45 | 6.00 | ± | 0.00 | 7.00 | ± | 0.00 | | 6.98 | ± | 2.18 | 7.98 | ± | 2.20 |  |
|  |  | Completeness | 100.00 | % |  | 100.00 | % |  | 100.00 | % |  | 100.00 | % |  | | 85.96 | % |  | 87.72 | % |  |  |
|  | Shannon | Interpolated | 0.25 | ± | 0.05 | 0.71 | ± | 0.05 | 0.88 | ± | 0.04 | 0.22 | ± | 0.10 | | 0.52 | ± | 0.07 | 0.83 | ± | 0.04 |  |
|  |  | Observed | 0.26 |  |  | 0.18 |  |  | 0.08 |  |  | 0.58 |  |  | | 0.69 |  |  | 1.16 |  |  |  |
|  | Exponential Shannon entropy | Interpolated | 1.29 | ± | 0.07 | 2.03 | ± | 0.10 | 2.42 | ± | 0.09 | 1.26 | ± | 0.14 | | 1.68 | ± | 0.12 | 2.30 | ± | 0.10 |  |
|  |  | Observed | 1.29 |  |  | 1.20 |  |  | 1.08 |  |  | 1.78 |  |  | | 1.99 |  |  | 3.18 |  |  |  |
|  | Simpson | Interpolated | 0.10 | ± | 0.03 | 0.40 | ± | 0.04 | 0.54 | ± | 0.01 | 0.09 | ± | 0.05 | | 0.24 | ± | 0.04 | 0.51 | ± | 0.03 |  |
|  |  | Observed | 0.10 |  |  | 0.07 |  |  | 0.02 |  |  | 0.35 |  |  | | 0.37 |  |  | 0.63 |  |  |  |

**Table S6.** Network measurements calculated for the observed incidence – based association matrix. These are tested against the network characteristics for 999 random networks controlling marginal sums of the random matrices (Patefield algorithm) and 999 random networks constraining the proportion of realized links (Vaznull algorithm) using one-sample t-tests. We do not present connectance for the random networks obtained with the Vaznull algorithm, as this network descriptor is constrained in the generation of random networks.

|  |  |  | **Patefield** | | | |  | **Vaznull** | | | |
| --- | --- | --- | --- | --- | --- | --- | --- | --- | --- | --- | --- |
| **Community subset** | **Network descriptor** | **Observed** | **Estimation** | **df** | **t** | ***P*** |  | **Estimation** | **df** | **t** | ***P*** |
| Full herbivore community | Connectance (C) | 0.70 | 0.94 | 999 | 968.66 | < 0.0010 |  | - | - | - | - |
|  | Nestedness (NODF) | 55.31 | 35.97 | 999 | - 147.70 | < 0.0010 |  | 67.66 | 999 | 166.89 | < 0.0010 |
|  | Specialization (H2) | 0.10 | 0.01 | 999 | - 4320.50 | < 0.0010 |  | 0.03 | 999 | - 898.20 | < 0.0010 |
| Chewing herbivore community | Connectance (C) | 0.71 | 0.96 | 999 | 729.85 | < 0.0010 |  | - | - | - | - |
|  | Nestedness (NODF) | 56.66 | 33.66 | 999 | - 119.74 | < 0.0010 |  | 62.52 | 999 | 46.71 | < 0.0010 |
|  | Specialization (H2) | 0.14 | 0.01 | 999 | - 3318.50 | < 0.0010 |  | 0.04 | 999 | - 834.41 | < 0.0010 |
| Sap-feeding herbivore community | Connectance (C) | 0.66 | 0.87 | 999 | 390.18 | < 0.0010 |  | - | - | - | - |
|  | Nestedness (NODF) | 60.54 | 47.14 | 999 | - 61.64 | < 0.0010 |  | 57.22 | 999 | - 15.16 | < 0.0010 |
|  | Specialization (H2) | 0.12 | 0.12 | 999 | - 1747.80 | < 0.0010 |  | 0.04 | 999 | - 338.97 | < 0.0010 |

**Table S7.** Network measurements calculated for the observed abundance – based association matrix. These are tested against the network characteristics for 999 random networks controlling marginal sums of the random matrices (Patefield algorithm) and 999 random networks constraining the proportion of realized links (Vaznull algorithm) using one-sample t-tests. We do not present connectance for the random networks obtained with the Vaznull algorithm, as this network descriptor is constrained in the generation of random networks.

|  |  |  | **Patefield** | | | |  | **Vaznull** | | | |
| --- | --- | --- | --- | --- | --- | --- | --- | --- | --- | --- | --- |
| **Community subset** | **Network descriptor** | **Observed** | **Estimation** | **df** | **t** | ***P*** |  | **Estimation** | **df** | **t** | ***P*** |
| Full herbivore community | Connectance (C) | 0.70 | 0.81 | 999 | 264.80 | < 0.0010 |  | - | - | - | - |
|  | Nestedness (NODF) | 52.90 | 72.18 | 999 | 224.74 | < 0.0010 |  | 79.68 | 999 | 409.57 | < 0.0010 |
|  | Specialization (H2) | 0.32 | 0.00 | 999 | -162440.00 | < 0.0010 |  | 0.00 | 999 | -105890.00 | < 0.0010 |
| Chewing herbivore community | Connectance (C) | 0.71 | 0.90 | 999 | 401.87 | < 0.0010 |  | - | - | - | - |
|  | Nestedness (NODF) | 58.79 | 61.02 | 999 | 16.42 | < 0.0010 |  | 70.78 | 999 | 105.09 | < 0.0010 |
|  | Specialization (H2) | 0.16 | 0.00 | 999 | -9244.60 | < 0.0010 |  | 0.01 | 999 | -4361.80 | < 0.0010 |
| Sap-feeding herbivore community | Connectance (C) | 0.66 | 0.66 | 999 | 4.83 | < 0.0010 |  | - | - | - | - |
|  | Nestedness (NODF) | 60.19 | 80.68 | 999 | 170.84 | < 0.0010 |  | 80.86 | 999 | 173.20 | < 0.0010 |
|  | Specialization (H2) | 0.41 | 0.01 | 999 | -104200.00 | < 0.0010 |  | 0.00 | 999 | -88018.00 | < 0.0010 |

**Table S8.** Specialization of plant species in their interactions with herbivores, observed for the incidence – based association matrix and its subsets based on the feeding guild of herbivores. Observed values are tested against 999 random networks controlling marginal sums of the random matrices (Patefield algorithm) and 999 random networks constraining the proportion of realized links (Vaznull algorithm) using one-sample t-tests.

|  |  | **Observed**  **(d_i_’)** | **Patefield** | | | |  | **Vaznull** | | | | | |
| --- | --- | --- | --- | --- | --- | --- | --- | --- | --- | --- | --- | --- | --- |
|  | **Plant species** |  | **Estimated** | **df** | **t** | ***P*** |  | **Estimated** | **df** | **t** | | ***P*** | |
| **Full herbivore community** | *Capsella bursa-pastoris* | 0.1228 | 0.0099 | 999 | -923.66 | < 0.0010 |  | 0.0331 | 999 | -135.30 | < 0.0010 | |  |
|  | *Camelina sativa* | 0.0900 | 0.0089 | 999 | -1033.72 | < 0.0010 |  | 0.0305 | 999 | -157.44 | < 0.0010 | |  |
|  | *Erysimum cheiranthoides* | 0.1247 | 0.0092 | 999 | -1000.22 | < 0.0010 |  | 0.0317 | 999 | -141.32 | < 0.0010 | |  |
|  | *Rorippa sylvestris* | 0.2214 | 0.0060 | 999 | -1563.97 | < 0.0010 |  | 0.0188 | 999 | -318.26 | < 0.0010 | |  |
|  | *Descurainia sophia* | 0.0910 | 0.0096 | 999 | -913.94 | < 0.0010 |  | 0.0335 | 999 | -132.01 | < 0.0010 | |  |
|  | *Brassica nigra* | 0.0476 | 0.0063 | 999 | -1475.51 | < 0.0010 |  | 0.0193 | 999 | -314.75 | < 0.0010 | |  |
|  | *Sinapis arvensis* | 0.0940 | 0.0079 | 999 | -1196.68 | < 0.0010 |  | 0.0258 | 999 | -201.86 | < 0.0010 | |  |
|  | *Rapistrum rugosum* | 0.0404 | 0.0063 | 999 | -1437.54 | < 0.0010 |  | 0.0198 | 999 | -295.21 | < 0.0010 | |  |
|  | *Raphanus raphanistrum* | 0.0349 | 0.0061 | 999 | -1595.93 | < 0.0010 |  | 0.0186 | 999 | -307.04 | < 0.0010 | |  |
|  | *Brassica rapa* | 0.0779 | 0.0065 | 999 | -1358.37 | < 0.0010 |  | 0.0209 | 999 | -254.77 | < 0.0010 | |  |
|  | *Sinapis alba* | 0.0862 | 0.0073 | 999 | -1293.74 | < 0.0010 |  | 0.0237 | 999 | -235.40 | < 0.0010 | |  |
|  | *Sisymbrium officinale* | 0.0930 | 0.0060 | 999 | -1495.63 | < 0.0010 |  | 0.0187 | 999 | -334.55 | < 0.0010 | |  |

**Table S8. Continued.**

|  |  | **Observed**  **(d_i_’)** | **Patefield** | | | |  | **Vaznull** | | | | | |
| --- | --- | --- | --- | --- | --- | --- | --- | --- | --- | --- | --- | --- | --- |
|  | **Plant species** |  | **Estimated** | **df** | **t** | ***P*** |  | **Estimated** | **df** | **t** | | ***P*** | |
| **Chewing herbivore community** | *Capsella bursa-pastoris* | 0.1024 | 0.0119 | 999 | -550.47 | < 0.0010 |  | 0.0384 | 999 | -81.49 | < 0.0010 | |  |
|  | *Camelina sativa* | 0.0897 | 0.0116 | 999 | -561.98 | < 0.0010 |  | 0.0374 | 999 | -89.16 | < 0.0010 | |  |
|  | *Erysimum cheiranthoides* | 0.1597 | 0.0096 | 999 | -668.20 | < 0.0010 |  | 0.0313 | 999 | -122.51 | < 0.0010 | |  |
|  | *Rorippa sylvestris* | 0.2266 | 0.0065 | 999 | -1065.34 | < 0.0010 |  | 0.0201 | 999 | -245.34 | < 0.0010 | |  |
|  | *Descurainia sophia* | 0.1408 | 0.0109 | 999 | -580.80 | < 0.0010 |  | 0.0346 | 999 | -102.31 | < 0.0010 | |  |
|  | *Brassica nigra* | 0.0602 | 0.0068 | 999 | -1004.90 | < 0.0010 |  | 0.0219 | 999 | -232.91 | < 0.0010 | |  |
|  | *Sinapis arvensis* | 0.1092 | 0.0080 | 999 | -827.63 | < 0.0010 |  | 0.0260 | 999 | -167.63 | < 0.0010 | |  |
|  | *Rapistrum rugosum* | 0.0561 | 0.0070 | 999 | -950.19 | < 0.0010 |  | 0.0221 | 999 | -221.35 | < 0.0010 | |  |
|  | *Raphanus raphanistrum* | 0.0460 | 0.0070 | 999 | -986.78 | < 0.0010 |  | 0.0220 | 999 | -208.08 | < 0.0010 | |  |
|  | *Brassica rapa* | 0.0913 | 0.0073 | 999 | -869.28 | < 0.0010 |  | 0.0241 | 999 | -185.03 | < 0.0010 | |  |
|  | *Sinapis alba* | 0.1159 | 0.0086 | 999 | -782.72 | < 0.0010 |  | 0.0270 | 999 | -157.04 | < 0.0010 | |  |
|  | *Sisymbrium officinale* | 0.1046 | 0.0065 | 999 | -1032.23 | < 0.0010 |  | 0.0202 | 999 | -244.25 | < 0.0010 | |  |
| **Sap-feeding herbivore community** | *Capsella bursa-pastoris* | 0.1656 | 0.0089 | 999 | -580.19 | < 0.0010 |  | 0.0271 | 999 | -98.66 | < 0.0010 | |  |
|  | *Camelina sativa* | 0.0964 | 0.0095 | 999 | -544.28 | < 0.0010 |  | 0.0306 | 999 | -83.57 | < 0.0010 | |  |
|  | *Erysimum cheiranthoides* | 0.0801 | 0.0138 | 999 | -321.73 | < 0.0010 |  | 0.0522 | 999 | -36.36 | < 0.0010 | |  |
|  | *Rorippa sylvestris* | 0.1351 | 0.0069 | 999 | -734.71 | < 0.0010 |  | 0.0196 | 999 | -136.57 | < 0.0010 | |  |
|  | *Descurainia sophia* | 0.0224 | 0.0078 | 999 | -697.06 | < 0.0010 |  | 0.0209 | 999 | -131.00 | < 0.0010 | |  |
|  | *Brassica nigra* | 0.0315 | 0.0061 | 999 | -898.48 | < 0.0010 |  | 0.0142 | 999 | -207.08 | < 0.0010 | |  |
|  | *Sinapis arvensis* | 0.1186 | 0.0088 | 999 | -578.40 | < 0.0010 |  | 0.0272 | 999 | -93.91 | < 0.0010 | |  |
|  | *Rapistrum rugosum* | 0.0117 | 0.0065 | 999 | -828.27 | < 0.0010 |  | 0.0169 | 999 | -161.81 | < 0.0010 | |  |
|  | *Raphanus raphanistrum* | 0.0122 | 0.0062 | 999 | -917.52 | < 0.0010 |  | 0.0146 | 999 | -201.03 | < 0.0010 | |  |
|  | *Brassica rapa* | 0.0617 | 0.0057 | 999 | -1012.41 | < 0.0010 |  | 0.0138 | 999 | -204.98 | < 0.0010 | |  |
|  | *Sinapis alba* | 0.0793 | 0.0072 | 999 | -771.08 | < 0.0010 |  | 0.0200 | 999 | -138.31 | < 0.0010 | |  |
|  | *Sisymbrium officinale* | 0.0676 | 0.0053 | 999 | -1043.59 | < 0.0010 |  | 0.0124 | 999 | -242.55 | < 0.0010 | |  |

**Table S9.** Specialization of plant species in their interactions with herbivores, observed for the abundance – based association matrix and its subsets based on the feeding guild of herbivores. Observed values are tested against 999 random networks controlling marginal sums of the random matrices (Patefield algorithm) and 999 random networks constraining the proportion of realized links (Vaznull algorithm) using one-sample t-tests.

|  |  | **Observed**  **(d_i_’)** | **Patefield** | | | |  | | | **Vaznull** | | | |  |
| --- | --- | --- | --- | --- | --- | --- | --- | --- | --- | --- | --- | --- | --- | --- |
|  | **Plant species** |  | **Estimated** | **df** | **t** | ***P*** | |  | **Estimated** | | **df** | **t** | ***P*** | |
| **Full herbivore community** | *Capsella bursa-pastoris* | 0.3520 | 0.0017 | 999 | -22322.46 | < 0.0010 | |  | 0.0026 | | 999 | -14255.38 | < 0.0010 | |
|  | *Camelina sativa* | 0.3746 | 0.0022 | 999 | -15367.22 | < 0.0010 | |  | 0.0033 | | 999 | -9996.53 | < 0.0010 | |
|  | *Erysimum cheiranthoides* | 0.4298 | 0.0008 | 999 | -51806.85 | < 0.0010 | |  | 0.0012 | | 999 | -33617.86 | < 0.0010 | |
|  | *Rorippa sylvestris* | 0.4661 | 0.0007 | 999 | -56565.57 | < 0.0010 | |  | 0.0012 | | 999 | -37586.25 | < 0.0010 | |
|  | *Descurainia Sophia* | 0.2851 | 0.0012 | 999 | -32931.51 | < 0.0010 | |  | 0.0019 | | 999 | -20093.87 | < 0.0010 | |
|  | *Brassica nigra* | 0.0355 | 0.0002 | 999 | -265735.40 | < 0.0010 | |  | 0.0002 | | 999 | -179088.80 | < 0.0010 | |
|  | *Sinapis arvensis* | 0.3911 | 0.0007 | 999 | -53881.72 | < 0.0010 | |  | 0.0012 | | 999 | -38749.63 | < 0.0010 | |
|  | *Rapistrum rugosum* | 0.0253 | 0.0003 | 999 | -149488.10 | < 0.0010 | |  | 0.0004 | | 999 | -84863.41 | < 0.0010 | |
|  | *Raphanus raphanistrum* | 0.1474 | 0.0001 | 999 | -381671.20 | < 0.0010 | |  | 0.0001 | | 999 | -268503.80 | < 0.0010 | |
|  | *Brassica rapa* | 0.0946 | 0.0002 | 999 | -227591.50 | < 0.0010 | |  | 0.0003 | | 999 | -140363.10 | < 0.0010 | |
|  | *Sinapis alba* | 0.1857 | 0.0005 | 999 | -87100.85 | < 0.0010 | |  | 0.0007 | | 999 | -57759.97 | < 0.0010 | |
|  | *Sisymbrium officinale* | 0.3223 | 0.0007 | 999 | -54450.51 | < 0.0010 | |  | 0.0012 | | 999 | -35322.04 | < 0.0010 | |

**Table S9. Continued.**

|  |  | **Observed**  **(d_i_’)** | **Patefield** | | | |  | **Vaznull** | | | | | |
| --- | --- | --- | --- | --- | --- | --- | --- | --- | --- | --- | --- | --- | --- |
|  | **Plant species** |  | **Estimated** | **df** | **t** | ***P*** |  | **Estimated** | **df** | **t** | | ***P*** | |
| **Chewing herbivore community** | *Capsella bursa-pastoris* | 0.0155 | 0.0030 | 999 | -2591.18 | < 0.0010 |  | 0.0062 | 999 | -876.04 | < 0.0010 | |  |
|  | *Camelina sativa* | 0.0986 | 0.0040 | 999 | -1465.51 | < 0.0010 |  | 0.0099 | 999 | -596.21 | < 0.0010 | |  |
|  | *Erysimum cheiranthoides* | 0.0536 | 0.0008 | 999 | -9417.77 | < 0.0010 |  | 0.0018 | 999 | -4300.51 | < 0.0010 | |  |
|  | *Rorippa sylvestris* | 0.0389 | 0.0008 | 999 | -9801.04 | < 0.0010 |  | 0.0018 | 999 | -4398.98 | < 0.0010 | |  |
|  | *Descurainia Sophia* | 0.0258 | 0.0014 | 999 | -5502.03 | < 0.0010 |  | 0.0028 | 999 | -2481.50 | < 0.0010 | |  |
|  | *Brassica nigra* | 0.0154 | 0.0007 | 999 | -11319.60 | < 0.0010 |  | 0.0015 | 999 | -5011.11 | < 0.0010 | |  |
|  | *Sinapis arvensis* | 0.0194 | 0.0008 | 999 | -9869.76 | < 0.0010 |  | 0.0017 | 999 | -4501.94 | < 0.0010 | |  |
|  | *Rapistrum rugosum* | 0.0089 | 0.0009 | 999 | -9013.26 | < 0.0010 |  | 0.0019 | 999 | -4282.88 | < 0.0010 | |  |
|  | *Raphanus raphanistrum* | 0.0398 | 0.0008 | 999 | -9506.56 | < 0.0010 |  | 0.0018 | 999 | -4185.73 | < 0.0010 | |  |
|  | *Brassica rapa* | 0.0340 | 0.0010 | 999 | -7595.93 | < 0.0010 |  | 0.0021 | 999 | -3805.13 | < 0.0010 | |  |
|  | *Sinapis alba* | 0.0319 | 0.0007 | 999 | -11566.61 | < 0.0010 |  | 0.0015 | 999 | -4833.32 | < 0.0010 | |  |
|  | *Sisymbrium officinale* | 0.0873 | 0.0012 | 999 | -7034.73 | < 0.0010 |  | 0.0024 | 999 | -3272.57 | < 0.0010 | |  |
| **Sap-feeding herbivore community** | *Capsella bursa-pastoris* | 0.5406 | 0.0006 | 999 | -22846.48 | < 0.0010 |  | 0.0007 | 999 | -20157.14 | < 0.0010 | |  |
|  | *Camelina sativa* | 0.3623 | 0.0021 | 999 | -6388.06 | < 0.0010 |  | 0.0024 | 999 | -5434.29 | < 0.0010 | |  |
|  | *Erysimum cheiranthoides* | 0.3315 | 0.0028 | 999 | -4272.79 | < 0.0010 |  | 0.0032 | 999 | -3754.22 | < 0.0010 | |  |
|  | *Rorippa sylvestris* | 0.6363 | 0.0006 | 999 | -23074.97 | < 0.0010 |  | 0.0007 | 999 | -19104.06 | < 0.0010 | |  |
|  | *Descurainia Sophia* | 0.2194 | 0.0009 | 999 | -14854.77 | < 0.0010 |  | 0.0010 | 999 | -12546.51 | < 0.0010 | |  |
|  | *Brassica nigra* | 0.0253 | 0.0001 | 999 | -416664.60 | < 0.0010 |  | 0.0001 | 999 | -430018.40 | < 0.0010 | |  |
|  | *Sinapis arvensis* | 0.4685 | 0.0006 | 999 | -21944.96 | < 0.0010 |  | 0.0007 | 999 | -19859.06 | < 0.0010 | |  |
|  | *Rapistrum rugosum* | 0.0062 | 0.0001 | 999 | -210066.20 | < 0.0010 |  | 0.0001 | 999 | -191436.60 | < 0.0010 | |  |
|  | *Raphanus raphanistrum* | 0.0624 | 0.0000 | 999 | -621119.20 | < 0.0010 |  | 0.0000 | 999 | -576754.00 | < 0.0010 | |  |
|  | *Brassica rapa* | 0.0754 | 0.0001 | 999 | -340580.20 | < 0.0010 |  | 0.0001 | 999 | -360071.50 | < 0.0010 | |  |
|  | *Sinapis alba* | 0.0369 | 0.0003 | 999 | -73064.19 | < 0.0010 |  | 0.0003 | 999 | -65153.73 | < 0.0010 | |  |
|  | *Sisymbrium officinale* | 0.3978 | 0.0004 | 999 | -49929.74 | < 0.0010 |  | 0.0005 | 999 | -47491.67 | < 0.0010 | |  |

**Table S10.** Specialization of herbivores in their interactions with plants, observed for the incidence-based and abundance–based association matrix. Values approximating 1 indicate high levels of specialization.

| **Species name** | **Feeding type** | **Incidence-based network (d_i_’)** | **Abundance-based network (d_i_’)** |
| --- | --- | --- | --- |
| *Athalia rosae* | Chewer | 0.2335 | 0.2813 |
| *Athalia* spp*.* | Chewer | 0.2042 | 0.2853 |
| *Autographa gamma* | Chewer | 0.1210 | 0.1721 |
| *Evergestis extimalis* | Chewer | 0.1916 | 0.0919 |
| Loopers | Chewer | 0.3678 | 0.4213 |
| *Mamestra brassicae* | Chewer | 0.1686 | 0.3036 |
| *Meligethes aeneus* | Chewer | 0.2067 | 0.1644 |
| *Murgantia histrionica* | Chewer | 0.2014 | 0.1833 |
| *Neocrepidodera transversa* | Chewer | 0.0575 | 0.2375 |
| *Phaedon cochleariae* | Chewer | 0.0792 | 0.0517 |
| *Phyllotreta atra* | Chewer | 0.0435 | 0.1282 |
| *Phyllotreta undulata* | Chewer | 0.0466 | 0.0641 |
| *Pieris brassicae* | Chewer | 0.1630 | 0.1436 |
| *Pieris rapae* | Chewer | 0.0438 | 0.1317 |
| *Plutella xylostella* | Chewer | 0.0166 | 0.3224 |
| *Aleyrodes proletella* | Sap feeder | 0.0717 | 0.1518 |
| *Aphis fabae* | Sap feeder | 0.3288 | 0.5969 |
| *Brevicoryne brassicae* | Sap feeder | 0.1777 | 0.3903 |
| Cicades | Sap feeder | 0.0393 | 0.1531 |
| *Lygus* spp. | Sap feeder | 0.0646 | 0.2048 |
| *Myzus persicae* | Sap feeder | 0.0185 | 0.2936 |
| *Myzus persicae* subsp. *nicotiana* | Sap feeder | 0.0597 | 0.0954 |
| *Dasineura sisymbrii* | Other | 0.4990 | 0.6183 |
| Leaf mining flies | Other | 0.0219 | 0.2092 |
| Thrips | Other | 0.0375 | 0.0848 |
| Weevils | Other | 0.1828 | 0.0999 |

**Table S11.** Results of linear models (LM) testing for differences among plant Lineages in diversity indices calculated for herbivore communities associated with plant species. Significant *P* values
(*P* < 0.05) are indicated in bold.

| **Community subset** | **Diversity index** | **df** | ***F*** | ***P*** |
| --- | --- | --- | --- | --- |
| Full herbivore community | Richness | 1 | 4.18 | 0.0682 |
|  | Shannon | 1 | 0.50 | 0.4946 |
|  | Exponential Shannon entropy | 1 | 0.19 | 0.6718 |
|  | Simpson | 1 | 0.39 | 0.5487 |
| Chewing herbivore community | Richness | 1 | 2.22 | 0.1674 |
|  | Shannon | 1 | 0.50 | 0.4958 |
|  | Exponential Shannon entropy | 1 | 0.25 | 0.6275 |
|  | Simpson | 1 | 0.23 | 0.6458 |
| Sap-feeding herbivore community | Richness | 1 | 10.74 | **0.0083** |
|  | Shannon | 1 | 1.18 | 0.3021 |
|  | Exponential Shannon entropy | 1 | 0.72 | 0.4161 |
|  | Simpson | 1 | 1.13 | 0.3112 |

**Table S12.** Results of Linear mixed models testing whether plant species differed in the diversity of plant-associated communities. We corrected for the hierarchical structure of our design by including plot as random intercepts in the model and accounted for heterogeneity of variance by allowing variation to be different for the different plant species. Significant results (*P* < 0.05) are indicated in bold.

| **Community subset** | **Diversity index** | **df** | ***ꭓ*^2^** | ***P*** |
| --- | --- | --- | --- | --- |
| Full herbivore community | Richness | 11 | 416.95 | **< 0.0001** |
|  | Shannon | 11 | 127.00 | **< 0.0001** |
|  | Exponential Shannon entropy | 11 | 135.91 | **< 0.0001** |
|  | Simpson | 11 | 106.35 | **< 0.0001** |
| Chewing herbivore community | Richness | 11 | 310.94 | **< 0.0001** |
|  | Shannon | 11 | 146.67 | **< 0.0001** |
|  | Exponential Shannon entropy | 11 | 134.76 | **< 0.0001** |
|  | Simpson | 11 | 134.81 | **< 0.0001** |
| Sap-feeding herbivore community | Richness | 11 | 312.88 | **< 0.0001** |
|  | Shannon | 11 | 201.24 | **< 0.0001** |
|  | Exponential Shannon entropy | 11 | 194.40 | **< 0.0001** |
|  | Simpson | 11 | 101.41 | **< 0.0001** |

**Table 13.** Results of Linear mixed models testing whether phylogenetic lineages differed in the diversity of plant-associated communities. We corrected the hierarchical structure of our design by including plot and plant species as random intercepts and accounted for heterogeneity of variance by allowing variation to be different for the two lineages. Significant results (*P* < 0.05) are indicated in bold.

|  |  |  |  |  |  | **Lineage I** | | |  | **Lineage II** | | |
| --- | --- | --- | --- | --- | --- | --- | --- | --- | --- | --- | --- | --- |
| **Community subset** | **Diversity index** | **df** | ***ꭓ2*** | ***P*** |  | **Estimate** |  | **SE** |  | **Estimate** |  | **SE** |
| Full herbivore community | Richness | 1 | 7.33 | **0.0066** |  | 5.2032 | ± | 0.6311 |  | 7.4496 | ± | 0.5360 |
|  | Shannon | 1 | 1.67 | 0.1967 |  | 0.9361 | ± | 0.1021 |  | 1.1099 | ± | 0.0868 |
|  | Exponential Shannon entropy | 1 | 1.82 | 0.1776 |  | 2.7531 | ± | 0.3220 |  | 3.3200 | ± | 0.2750 |
|  | Simpson | 1 | 1.01 | 0.3149 |  | 0.4640 | ± | 0.0463 |  | 0.5250 | ± | 0.0393 |
| Chewing herbivore community | Richness | 1 | 6.54 | **0.0105** |  | 2.4000 | ± | 0.3540 |  | 3.5886 | ± | 0.3000 |
|  | Shannon | 1 | 4.41 | **0.0358** |  | 0.3290 | ± | 0.0626 |  | 0.5007 | ± | 0.0522 |
|  | Exponential Shannon entropy | 1 | 3.38 | 0.0662 |  | 1.4512 | ± | 0.1118 |  | 1.7202 | ± | 0.0941 |
|  | Simpson | 1 | 1.63 | 0.2024 |  | 0.1900 | ± | 0.0372 |  | 0.2517 | ± | 0.0307 |
| Sap-feeding herbivore community | Richness | 1 | 9.68 | **0.0019** |  | 1.2300 | ± | 0.2368 |  | 2.1971 | ± | 0.2005 |
|  | Shannon | 1 | 2.65 | 0.1035 |  | 0.1950 | ± | 0.0804 |  | 0.3659 | ± | 0.0675 |
|  | Exponential Shannon entropy | 1 | 2.14 | 0.1431 |  | 1.2800 | ± | 0.1330 |  | 1.5414 | ± | 0.1120 |
|  | Simpson | 1 | 0.98 | 0.3215 |  | 0.3010 | ± | 0.0623 |  | 0.2220 | ± | 0.0491 |

**Table S14.** Sørensen β diversity among plants decomposed in variation due to species replacement (i.e. turnover in species) and variation due to differences in the number of herbivore species (i.e. nestedness) interacting with individual plants. We further calculated the average pairwise dissimilarity and its standard deviation between two plant individuals of the same species.

|  |  |  |  |  |  | **Average β** | | |  | **Average turnover** | | |  | **Average nestedness** | | |
| --- | --- | --- | --- | --- | --- | --- | --- | --- | --- | --- | --- | --- | --- | --- | --- | --- |
|  | **Plant species** | **Tota**l **β** | **Turnover** | **Nestedness** |  | **Estimate** | | **SD** |  | **Estimate** | | **SD** |  | **Estimate** | | **SD** |
| **Full herbivore community** | *Capsella bursa-pastoris* | 0.8672 | 0.8074 | 0.0598 |  | 0.3891 | ± | 0.1846 |  | 0.2742 | ± | 0.2292 |  | 0.1149 | ± | 0.1145 |
|  | *Camelina sativa* | 0.8647 | 0.7936 | 0.0711 |  | 0.3585 | ± | 0.1738 |  | 0.2473 | ± | 0.2046 |  | 0.1112 | ± | 0.0969 |
|  | *Erysimum cheiranthoides* | 0.8765 | 0.7694 | 0.1071 |  | 0.2782 | ± | 0.1430 |  | 0.1419 | ± | 0.1571 |  | 0.1363 | ± | 0.1187 |
|  | *Rorippa sylvestris* | 0.8897 | 0.8196 | 0.0701 |  | 0.3587 | ± | 0.1175 |  | 0.2313 | ± | 0.1461 |  | 0.1274 | ± | 0.1051 |
|  | *Descurainia Sophia* | 0.9168 | 0.8709 | 0.0459 |  | 0.4945 | ± | 0.1672 |  | 0.3598 | ± | 0.2148 |  | 0.1347 | ± | 0.1235 |
|  | *Brassica nigra* | 0.8960 | 0.8176 | 0.0784 |  | 0.3421 | ± | 0.1355 |  | 0.2022 | ± | 0.1397 |  | 0.1399 | ± | 0.1364 |
|  | *Sinapis arvensis* | 0.8854 | 0.8184 | 0.0670 |  | 0.3055 | ± | 0.1272 |  | 0.1960 | ± | 0.1554 |  | 0.1095 | ± | 0.0937 |
|  | *Rapistrum rugosum* | 0.8705 | 0.7903 | 0.0802 |  | 0.2723 | ± | 0.1088 |  | 0.1633 | ± | 0.1146 |  | 0.1090 | ± | 0.1014 |
|  | *Raphanus raphanistrum* | 0.8879 | 0.8146 | 0.0733 |  | 0.3219 | ± | 0.1295 |  | 0.1992 | ± | 0.1436 |  | 0.1227 | ± | 0.1102 |
|  | *Brassica rapa* | 0.8956 | 0.8273 | 0.0683 |  | 0.3355 | ± | 0.1304 |  | 0.2146 | ± | 0.1387 |  | 0.1209 | ± | 0.0958 |
|  | *Sinapis alba* | 0.8785 | 0.7797 | 0.0988 |  | 0.2732 | ± | 0.1135 |  | 0.1390 | ± | 0.1397 |  | 0.1342 | ± | 0.1135 |
|  | *Sisymbrium officinale* | 0.8964 | 0.8285 | 0.0679 |  | 0.3355 | ± | 0.1089 |  | 0.2136 | ± | 0.1239 |  | 0.1220 | ± | 0.0953 |

**Table S14. Continued.**

|  |  |  |  |  |  | **Average β** | | |  | **Average turnover** | | |  | **Average nestedness** | | |
| --- | --- | --- | --- | --- | --- | --- | --- | --- | --- | --- | --- | --- | --- | --- | --- | --- |
|  | **Plant species** | **Tota**l **β** | **Turnover** | **Nestedness** |  | **Estimate** | | **SD** |  | **Estimate** | | **SD** |  | **Estimate** | | **SD** |
| **Chewing herbivore community** | *Capsella bursa-pastoris* | 0.8696 | 0.7414 | 0.1282 |  | 0.3962 | ± | 0.2960 |  | 0.1881 | ± | 0.3038 |  | 0.1824 | ± | 0.1789 |
|  | *Camelina sativa* | 0.8878 | 0.8147 | 0.0731 |  | 0.4256 | ± | 0.3015 |  | 0.2721 | ± | 0.3720 |  | 0.1535 | ± | 0.1735 |
|  | *Erysimum cheiranthoides* | 0.7895 | 0.3933 | 0.3962 |  | 0.1327 | ± | 0.1351 |  | 0.0198 | ± | 0.0787 |  | 0.1130 | ± | 0.1243 |
|  | *Rorippa sylvestris* | 0.9062 | 0.8061 | 0.1001 |  | 0.4326 | ± | 0.1674 |  | 0.2349 | ± | 0.2230 |  | 0.1977 | ± | 0.1666 |
|  | *Descurainia Sophia* | 0.9018 | 0.8144 | 0.0874 |  | 0.3692 | ± | 0.1890 |  | 0.1744 | ± | 0.2384 |  | 0.1948 | ± | 0.1825 |
|  | *Brassica nigra* | 0.9131 | 0.8308 | 0.0824 |  | 0.4047 | ± | 0.1795 |  | 0.2183 | ± | 0.2190 |  | 0.1864 | ± | 0.1736 |
|  | *Sinapis arvensis* | 0.8761 | 0.6928 | 0.1833 |  | 0.2795 | ± | 0.1605 |  | 0.0949 | ± | 0.1672 |  | 0.1846 | ± | 0.1520 |
|  | *Rapistrum rugosum* | 0.8873 | 0.7670 | 0.1203 |  | 0.3251 | ± | 0.1651 |  | 0.1515 | ± | 0.1856 |  | 0.1735 | ± | 0.1547 |
|  | *Raphanus raphanistrum* | 0.8947 | 0.8015 | 0.0932 |  | 0.3378 | ± | 0.1552 |  | 0.1850 | ± | 0.1860 |  | 0.1528 | ± | 0.1304 |
|  | *Brassica rapa* | 0.8890 | 0.7736 | 0.1154 |  | 0.3106 | ± | 0.1859 |  | 0.1366 | ± | 0.1976 |  | 0.1740 | ± | 0.1622 |
|  | *Sinapis alba* | 0.8856 | 0.7220 | 0.1636 |  | 0.2792 | ± | 0.1719 |  | 0.0981 | ± | 0.1698 |  | 0.1811 | ± | 0.1594 |
|  | *Sisymbrium officinale* | 0.9099 | 0.8225 | 0.0874 |  | 0.3883 | ± | 0.1610 |  | 0.2113 | ± | 0.1993 |  | 0.1770 | ± | 0.1490 |
| **Sap-feeding herbivore community** | *Capsella bursa-pastoris* | 0.8631 | 0.7402 | 0.1229 |  | 0.3812 | ± | 0.4054 |  | 0.1855 | ± | 0.3786 |  | 0.1159 | ± | 0.1693 |
|  | *Camelina sativa* | 0.8672 | 0.5294 | 0.3378 |  | 0.4382 | ± | 0.4347 |  | 0.0764 | ± | 0.254 |  | 0.1210 | ± | 0.1731 |
|  | *Erysimum cheiranthoides* | 0.9470 | 0.6951 | 0.2519 |  | 0.7776 | ± | 0.3908 |  | 0.2011 | ± | 0.3981 |  | 0.0930 | ± | 0.1498 |
|  | *Rorippa sylvestris* | 0.8581 | 0.5161 | 0.3420 |  | 0.2564 | ± | 0.1908 |  | 0.0411 | ± | 0.1358 |  | 0.2154 | ± | 0.1822 |
|  | *Descurainia Sophia* | 0.9200 | 0.7698 | 0.1502 |  | 0.6088 | ± | 0.3589 |  | 0.2514 | ± | 0.3843 |  | 0.1964 | ± | 0.1963 |
|  | *Brassica nigra* | 0.8960 | 0.7569 | 0.1391 |  | 0.3401 | ± | 0.1928 |  | 0.1290 | ± | 0.2097 |  | 0.2110 | ± | 0.1849 |
|  | *Sinapis arvensis* | 0.8617 | 0.5986 | 0.2631 |  | 0.2219 | ± | 0.2863 |  | 0.0567 | ± | 0.2104 |  | 0.1321 | ± | 0.1701 |
|  | *Rapistrum rugosum* | 0.8958 | 0.7125 | 0.1832 |  | 0.3232 | ± | 0.1983 |  | 0.0894 | ± | 0.1876 |  | 0.2339 | ± | 0.1938 |
|  | *Raphanus raphanistrum* | 0.8922 | 0.7286 | 0.1636 |  | 0.3289 | ± | 0.2056 |  | 0.1153 | ± | 0.2190 |  | 0.2136 | ± | 0.1827 |
|  | *Brassica rapa* | 0.8910 | 0.7480 | 0.1430 |  | 0.3277 | ± | 0.2021 |  | 0.1270 | ± | 0.1986 |  | 0.2007 | ± | 0.1896 |
|  | *Sinapis alba* | 0.8962 | 0.7545 | 0.1417 |  | 0.3024 | ± | 0.1927 |  | 0.0988 | ± | 0.1995 |  | 0.2036 | ± | 0.1806 |
|  | *Sisymbrium officinale* | 0.8747 | 0.7002 | 0.1744 |  | 0.2733 | ± | 0.1762 |  | 0.0952 | ± | 0.1771 |  | 0.1781 | ± | 0.1615 |

**Table S15.** Pairwise permutational comparisons of the composition of herbivore communities (incidence-based data) associated with plant species. Analyses were performed by PERMANOVA using 999 permutations and accounting for dependency of observations in the permutational design. Values above the diagonals represent pseudo-*F* values, values below the diagonals represent *P* values adjusted with False Discovery Rate (FDR) correction. Significant *P* values (*P* < 0.05) are indicated in bold.

|  |  | *C. bursa-pastoris* | *C. sativa* | *E. cheiranthoides* | *R. sylvestris* | *D. sophia* | *B. nigra* | *S. arvensis* | *R. rugosum* | *R. raphanistrum* | *B. rapa* | *S. alba* | *S. officinale* |
| --- | --- | --- | --- | --- | --- | --- | --- | --- | --- | --- | --- | --- | --- |
| **Full herbivore community** | *Capsella bursa-pastoris* |  | 3.91 | 12.37 | 42.11 | 5.65 | 20.67 | 14.35 | 23.92 | 24.45 | 15.08 | 18.98 | 25.013 |
|  | *Camelina sativa* | **0.0031** |  | 8.47 | 46.72 | 10.17 | 17.03 | 14.85 | 17.31 | 14.93 | 21.48 | 17.91 | 28.41 |
|  | *Erysimum cheiranthoides* | **0.0022** | **0.0012** |  | 66.96 | 12.40 | 40.06 | 31.63 | 41.89 | 45.64 | 42.66 | 49.93 | 50.98 |
|  | *Rorippa sylvestris* | **0.0012** | **0.0012** | **0.0012** |  | 26.08 | 46.42 | 79.76 | 54.88 | 49.27 | 51.50 | 74.75 | 31.46 |
|  | *Descurainia sophia* | **0.0031** | **0.0012** | **0.0012** | **0.0012** |  | 17.19 | 18.07 | 17.80 | 17.82 | 13.51 | 20.30 | 14.08 |
|  | *Brassica nigra* | **0.0012** | **0.0012** | **0.0012** | **0.0012** | **0.0012** |  | 18.15 | 3.32 | 5.66 | 9.77 | 16.28 | 18.76 |
|  | *Sinapis arvensis* | **0.0012** | **0.0012** | **0.0012** | **0.0012** | **0.0012** | **0.0012** |  | 15.84 | 17.13 | 37.82 | 8.09 | 49.63 |
|  | *Rapistrum rugosum* | **0.0012** | **0.0012** | **0.0012** | **0.0012** | **0.0012** | 0.1590 | **0.0022** |  | 5.73 | 23.81 | 14.28 | 24.46 |
|  | *Raphanus raphanistrum* | **0.0022** | **0.0012** | **0.0012** | **0.0012** | **0.0012** | **0.0072** | **0.0012** | **0.0345** |  | 23.09 | 17.52 | 25.73 |
|  | *Brassica rapa* | **0.0012** | **0.0022** | **0.0012** | **0.0012** | **0.0012** | **0.0022** | **0.0012** | **0.0012** | **0.0012** |  | 37.49 | 14.51 |
|  | *Sinapis alba* | **0.0012** | **0.0012** | **0.0012** | **0.0012** | **0.0012** | **0.0012** | **0.0022** | **0.0012** | **0.0012** | **0.0022** |  | 46.42 |
|  | *Sisymbrium officinale* | **0.0012** | **0.0012** | **0.0012** | **0.0012** | **0.0012** | **0.0012** | **0.0012** | **0.0012** | **0.0012** | **0.0022** | **0.0012** |  |

**Table S15. Continued.**

|  |  | *C. bursa-pastoris* | *C. sativa* | *E. cheiranthoides* | *R. sylvestris* | *D. sophia* | *B. nigra* | *S. arvensis* | *R. rugosum* | *R. raphanistrum* | *B. rapa* | *S. alba* | *S. officinale* |
| --- | --- | --- | --- | --- | --- | --- | --- | --- | --- | --- | --- | --- | --- |
| **Chewing herbivore community** | *Capsella bursa-pastoris* |  | 0.45 | 0.57 | 22.58 | 3.84 | 14.19 | 16.16 | 19.96 | 19.13 | 11.56 | 30.38 | 20.85 |
|  | *Camelina sativa* | 0.6390 |  | - | 22.35 | 6.26 | 14.31 | 14.54 | 17.36 | 16.18 | 10.78 | 28.98 | 18.54 |
|  | *Erysimum cheiranthoides* | 0.4890 | **-** |  | 71.56 | 21.06 | 42.97 | 54.72 | 61.82 | 55.60 | 35.10 | 133.96 | 58.28 |
|  | *Rorippa sylvestris* | **0.0012** | **0.0012** | **0.0012** |  | 25.36 | 31.11 | 56.08 | 46.35 | 52.67 | 36.61 | 73.13 | 25.56 |
|  | *Descurainia sophia* | **0.0195** | **0.0080** | **0.0012** | **0.0012** |  | 27.61 | 28.03 | 36.48 | 37.05 | 27.83 | 39.28 | 28.08 |
|  | *Brassica nigra* | **0.0012** | **0.0012** | **0.0012** | **0.0012** | **0.0012** |  | 18.09 | 3.14 | 6.07 | 6.69 | 27.23 | 17.53 |
|  | *Sinapis arvensis* | **0.0012** | **0.0012** | **0.0012** | **0.0012** | **0.0012** | **0.0012** |  | 9.71 | 6.05 | 42.69 | 6.34 | 47.59 |
|  | *Rapistrum rugosum* | **0.0012** | **0.0012** | **0.0012** | **0.0012** | **0.0012** | 0.1824 | **0.0046** |  | 1.51 | 18.62 | 20.16 | 25.27 |
|  | *Raphanus raphanistrum* | **0.0012** | **0.0012** | **0.0012** | **0.0012** | **0.0012** | **0.0256** | **0.0165** | 0.5484 |  | 26.35 | 14.48 | 38.33 |
|  | *Brassica rapa* | **0.0024** | **0.0012** | **0.0012** | **0.0012** | **0.0012** | **0.0035** | **0.0012** | **0.0012** | **0.0012** |  | 74.72 | 12.97 |
|  | *Sinapis alba* | **0.0012** | **0.0012** | **0.0012** | **0.0012** | **0.0012** | **0.0012** | **0.0165** | **0.0012** | **0.0012** | **0.0012** |  | 78.31 |
|  | *Sisymbrium officinale* | **0.0012** | **0.0012** | **0.0012** | **0.0012** | **0.0012** | **0.0012** | **0.0012** | **0.0012** | **0.0012** | **0.0012** | **0.0012** |  |

**Table S15. Continued.**

|  |  | *C. bursa-pastoris* | *C. sativa* | *E. cheiranthoides* | *R. sylvestris* | *D. sophia* | *B. nigra* | *S. arvensis* | *R. rugosum* | *R. raphanistrum* | *B. rapa* | *S. alba* | *S. officinale* |
| --- | --- | --- | --- | --- | --- | --- | --- | --- | --- | --- | --- | --- | --- |
| **Sap-feeding herbivore community** | *Capsella bursa-pastoris* |  | 1.79 | 4.64 | 43.14 | 7.97 | 6.79 | 1.32 | 3.43 | 10.79 | 13.45 | 2.71 | 17.28 |
|  | *Camelina sativa* | 0.2017 |  | 14.21 | 131.84 | 20.74 | 17.63 | 0.38 | 9.86 | 28.99 | 34.75 | 4.00 | 69.60 |
|  | *Erysimum cheiranthoides* | 0.2408 | 0.1540 |  | 29.93 | 1.89 | 5.13 | 10.38 | 0.47 | 2.87 | 11.29 | 7.34 | 9.29 |
|  | *Rorippa sylvestris* | **0.0049** | **0.0030** | **0.0136** |  | 13.37 | 57.12 | 110.29 | 58.05 | 33.90 | 60.22 | 89.79 | 23.89 |
|  | *Descurainia sophia* | **0.0226** | **0.0030** | 0.4176 | **0.0083** |  | 6.29 | 20.58 | 4.00 | 1.52 | 9.55 | 16.39 | 1.02 |
|  | *Brassica nigra* | **0.0100** | **0.0030** | 0.2408 | **0.0030** | **0.0424** |  | 15.35 | 2.47 | 1.88 | 1.71 | 11.86 | 14.43 |
|  | *Sinapis arvensis* | 0.2220 | 0.6834 | 0.0660 | **0.0030** | **0.0030** | **0.0030** |  | 8.74 | 25.19 | 30.28 | 2.14 | 51.04 |
|  | *Rapistrum rugosum* | 0.1333 | 0.0537 | 0.8140 | **0.0030** | 0.1831 | 0.3664 | **0.0226** |  | 5.13 | 11.90 | 7.84 | 15.10 |
|  | *Raphanus raphanistrum* | **0.0030** | **0.0049** | 0.4348 | **0.0030** | **0.0035** | 0.4348 | **0.0030** | 0.1293 |  | 3.70 | 18.43 | 5.82 |
|  | *Brassica rapa* | **0.0066** | **0.0030** | **0.0386** | **0.0030** | **0.0083** | 0.4176 | **0.0030** | **0.0165** | 0.1293 |  | 24.13 | 17.52 |
|  | *Sinapis alba* | **0.0363** | **0.0305** | 0.0947 | **0.0030** | **0.0049** | **0.0049** | 0.1250 | **0.0165** | **0.0030** | **0.0030** |  | 42.29 |
|  | *Sisymbrium officinale* | **0.0049** | **0.0030** | 0.1599 | **0.0030** | 0.5342 | **0.0066** | **0.0030** | **0.0066** | 0.0585 | **0.0030** | **0.0030** |  |

**Table S16.** Pairwise permutational comparisons of the structure of herbivore communities (expressed as log (x + 1) transformed abundance data) associated with plant species. Analyses were performed by PERMANOVA using 999 permutations and accounting for dependency of observations. Values above the diagonals represent pseudo-*F* values, values below the diagonals represent *P* values adjusted with False Discovery Rate (FDR) correction. Significant *P* values (*P* < 0.05) are indicated in bold.

|  |  | *C. bursa-pastoris* | *C. sativa* | *E. cheiranthoides* | *R. sylvestris* | *D. sophia* | *B. nigra* | *S. arvensis* | *R. rugosum* | *R. raphanistrum* | *B. rapa* | *S. alba* | *S. officinale* |
| --- | --- | --- | --- | --- | --- | --- | --- | --- | --- | --- | --- | --- | --- |
| **Full herbivore community** | *Capsella bursa-pastoris* |  | 10.94 | 19.73 | 43.37 | 11.31 | 24.27 | 13.63 | 24.42 | 21.77 | 17.24 | 22.22 | 21.13 |
|  | *Camelina sativa* | **0.0011** |  | 36.23 | 81.35 | 36.56 | 44.18 | 33.81 | 39.97 | 41.56 | 42.64 | 36.08 | 39.69 |
|  | *Erysimum cheiranthoides* | **0.0011** | **0.0011** |  | 61.57 | 17.00 | 43.87 | 22.96 | 41.27 | 40.88 | 46.51 | 45.06 | 44.27 |
|  | *Rorippa sylvestris* | **0.0011** | **0.0011** | **0.0011** |  | 28.58 | 56.25 | 64.45 | 60.01 | 45.60 | 58.44 | 81.43 | 38.37 |
|  | *Descurainia sophia* | **0.0021** | **0.0011** | **0.0011** | **0.0011** |  | 34.05 | 23.47 | 34.57 | 29.72 | 27.86 | 33.38 | 22.00 |
|  | *Brassica nigra* | **0.0011** | **0.0011** | **0.0011** | **0.0011** | **0.0011** |  | 19.98 | 3.08 | 5.20 | 7.92 | 19.83 | 18.33 |
|  | *Sinapis arvensis* | **0.0011** | **0.0011** | **0.0011** | **0.0011** | **0.0011** | **0.0011** |  | 14.40 | 12.88 | 27.36 | 6.06 | 32.38 |
|  | *Rapistrum rugosum* | **0.0011** | **0.0011** | **0.0011** | **0.0011** | **0.0011** | 0.1130 | **0.0011** |  | 4.12 | 18.66 | 14.75 | 20.47 |
|  | *Raphanus raphanistrum* | **0.0011** | **0.0011** | **0.0011** | **0.0011** | **0.0011** | **0.0021** | **0.0011** | **0.0345** |  | 16.60 | 15.97 | 19.22 |
|  | *Brassica rapa* | **0.0011** | **0.0011** | **0.0011** | **0.0011** | **0.0011** | **0.0011** | **0.0011** | **0.0011** | **0.0011** |  | 32.19 | 12.80 |
|  | *Sinapis alba* | **0.0021** | **0.0011** | **0.0011** | **0.0011** | **0.0011** | **0.0011** | **0.0011** | **0.0011** | **0.0011** | **0.0011** |  | 35.96 |
|  | *Sisymbrium officinale* | **0.0011** | **0.0011** | **0.0011** | **0.0011** | **0.0021** | **0.0011** | **0.0011** | **0.0011** | **0.0011** | **0.0011** | **0.0011** |  |

**Table S16. Continued.**

|  |  | *C. bursa-pastoris* | *C. sativa* | *E. cheiranthoides* | *R. sylvestris* | *D. sophia* | *B. nigra* | *S. arvensis* | *R. rugosum* | *R. raphanistrum* | *B. rapa* | *S. alba* | *S. officinale* |
| --- | --- | --- | --- | --- | --- | --- | --- | --- | --- | --- | --- | --- | --- |
| **Chewing herbivore community** | *Capsella bursa-pastoris* |  | 4.14 | 23.32 | 27.57 | 9.54 | 23.70 | 26.12 | 24.31 | 32.78 | 19.03 | 40.83 | 15.99 |
|  | *Camelina sativa* | **0.0268** |  | 25.36 | 38.22 | 23.47 | 34.88 | 33.78 | 33.03 | 36.97 | 29.38 | 45.17 | 24.34 |
|  | *Erysimum cheiranthoides* | **0.0012** | **0.0012** |  | 54.16 | 50.27 | 37.19 | 40.82 | 39.95 | 55.07 | 46.02 | 90.64 | 37.63 |
|  | *Rorippa sylvestris* | **0.0012** | **0.0012** | **0.0012** |  | 26.50 | 17.23 | 33.93 | 24.98 | 41.94 | 30.22 | 44.29 | 17.77 |
|  | *Descurainia sophia* | **0.0033** | **0.0012** | **0.0012** | **0.0012** |  | 32.07 | 38.05 | 38.58 | 54.79 | 40.37 | 56.08 | 27.00 |
|  | *Brassica nigra* | **0.0012** | **0.0012** | **0.0012** | **0.0012** | **0.0012** |  | 8.87 | 3.16 | 11.80 | 11.76 | 15.22 | 16.39 |
|  | *Sinapis arvensis* | **0.0012** | **0.0012** | **0.0012** | **0.0012** | **0.0012** | **0.0022** |  | 4.95 | 4.06 | 36.02 | 4.58 | 30.86 |
|  | *Rapistrum rugosum* | **0.0022** | **0.0012** | **0.0012** | **0.0012** | **0.0012** | **0.0241** | **0.0012** |  | 6.21 | 14.16 | 14.80 | 13.52 |
|  | *Raphanus raphanistrum* | **0.0012** | **0.0012** | **0.0012** | **0.0012** | **0.0012** | **0.0012** | 0.0930 | **0.0128** |  | 36.37 | 8.86 | 31.73 |
|  | *Brassica rapa* | **0.0012** | **0.0012** | **0.0012** | **0.0012** | **0.0012** | **0.0012** | **0.0012** | **0.0012** | **0.0012** |  | 66.91 | 9.35 |
|  | *Sinapis alba* | **0.0012** | **0.0012** | **0.0012** | **0.0012** | **0.0012** | **0.0012** | **0.0284** | **0.0012** | **0.0054** | **0.0012** |  | 48.55 |
|  | *Sisymbrium officinale* | **0.0012** | **0.0012** | **0.0012** | **0.0012** | **0.0012** | **0.0012** | **0.0012** | **0.0012** | **0.0012** | **0.0012** | **0.0012** |  |

**Table S16. Continued.**

|  |  | *C. bursa-pastoris* | *C. sativa* | *E. cheiranthoides* | *R. sylvestris* | *D. sophia* | *B. nigra* | *S. arvensis* | *R. rugosum* | *R. raphanistrum* | *B. rapa* | *S. alba* | *S. officinale* |
| --- | --- | --- | --- | --- | --- | --- | --- | --- | --- | --- | --- | --- | --- |
| **Sap-feeding herbivore community** | *Capsella bursa-pastoris* |  | 2.18 | 4.79 | 51.83 | 16.50 | 10.06 | 3.07 | 8.27 | 10.12 | 12.86 | 3.77 | 19.78 |
|  | *Camelina sativa* | 0.1754 |  | 8.35 | 131.90 | 34.56 | 17.64 | 0.20 | 13.69 | 18.14 | 23.97 | 3.52 | 55.76 |
|  | *Erysimum cheiranthoides* | 0.1518 | 0.3753 |  | 56.50 | 10.54 | 5.45 | 10.27 | - | 2.67 | 10.62 | 4.82 | 16.83 |
|  | *Rorippa sylvestris* | **0.0062** | **0.0026** | **0.0080** |  | 9.20 | 51.85 | 165.30 | 51.99 | 37.56 | 66.91 | 100.33 | 23.88 |
|  | *Descurainia sophia* | **0.0142** | **0.0026** | 0.1754 | **0.0490** |  | 10.10 | 46.56 | 6.41 | 4.21 | 16.35 | 29.03 | - |
|  | *Brassica nigra* | **0.0026** | **0.0026** | 0.2008 | **0.0026** | **0.0256** |  | 23.65 | 3.16 | 1.52 | 0.79 | 13.53 | 10.57 |
|  | *Sinapis arvensis* | **0.0327** | 0.8640 | 0.1434 | **0.0026** | **0.0026** | **0.0026** |  | 18.95 | 24.67 | 30.99 | 3.46 | 69.80 |
|  | *Rapistrum rugosum* | **0.0152** | **0.0213** | - | **0.0026** | 0.1637 | 0.1851 | **0.0046** |  | 0.57 | 8.75 | 10.27 | 10.30 |
|  | *Raphanus raphanistrum* | **0.0136** | **0.0062** | 0.4721 | **0.0026** | 0.1792 | 0.3776 | **0.0026** | 0.7396 |  | 5.23 | 13.70 | 5.24 |
|  | *Brassica rapa* | **0.0026** | **0.0026** | **0.0445** | **0.0026** | **0.0026** | 0.4986 | **0.0026** | **0.0142** | **0.0274** |  | 18.12 | 17.61 |
|  | *Sinapis alba* | **0.0142** | **0.0320** | 0.1987 | **0.0026** | **0.0046** | **0.0026** | **0.0298** | **0.0152** | **0.0026** | **0.0026** |  | 41.47 |
|  | *Sisymbrium officinale* | **0.0046** | **0.0062** | 0.0547 | **0.0026** | **-** | **0.0026** | **0.0026** | **0.0274** | 0.0797 | **0.0026** | **0.0026** |  |

**Table S17.** Condensed results of the SIMPER analysis, summarising the minimum, average and maximum percentage of the total differences among any of the plant species explained by variation in the incidence of each herbivore (morpho) species.

| **Species name** | **Minimum contribution (%)** | **Average contribution (%)** | **Maximum contribution (%)** |
| --- | --- | --- | --- |
| *Neocrepidodera transversa* | 2.23 | 4.19 | 9.08 |
| *Phaedon cochleariae* | 0.00 | 0.54 | 1.51 |
| *Phyllotreta atra* | 0.35 | 1.40 | 2.48 |
| *Phyllotreta undulata* | 1.22 | 3.50 | 6.99 |
| *Meligethes aeneus* | 0.00 | 3.80 | 9.48 |
| *Murgantia histrionica* | 0.00 | 0.27 | 0.96 |
| *Athalia rosae* | 0.00 | 0.79 | 3.35 |
| *Athalia* spp. | 0.00 | 0.54 | 2.28 |
| Loopers | 0.00 | 0.94 | 5.51 |
| *Evergestis extimalis* | 0.00 | 0.11 | 0.55 |
| *Autographa gamma* | 0.00 | 0.85 | 2.58 |
| *Mamestra brassicae* | 0.00 | 1.62 | 4.44 |
| *Pieris brassicae* | 0.00 | 0.26 | 0.95 |
| *Pieris rapae* | 1.51 | 3.82 | 7.00 |
| *Plutella xylostella* | 0.00 | 0.49 | 3.19 |
| Cicades | 0.66 | 1.72 | 3.60 |
| *Aleyrodes proletella* | 0.00 | 0.00 | 0.00 |
| *Aphis fabae* | 0.00 | 0.17 | 1.25 |
| *Brevicoryne brassicae* | 0.00 | 2.40 | 7.12 |
| *Myzus persicae* | 0.00 | 2.45 | 6.86 |
| *Myzus persicae* subsp. *Nicotiana* | 0.21 | 1.50 | 3.54 |
| *Lygus* spp. | 0.48 | 4.12 | 7.96 |
| Leaf mining flies | 0.30 | 2.65 | 6.96 |
| Weevils | 0.64 | 2.79 | 5.80 |
| *Dasineura sisymbrii* | 0.00 | 1.13 | 7.97 |
| Thrips | 1.39 | 4.18 | 7.84 |

**Table S18.** Pairwise comparisons of SIMPER analysis, summarising the percentage and standard deviation of the total differences among plant species explained by variation in the incidence of each herbivore (morpho) species. Significant contributions to the pairwise similarity (*P* < 0.05) are indicated in bold.

|  | ***Brassica nigra*** | | | ***Brassica nigra*** | | | ***Brassica nigra*** | | | ***Brassica nigra*** | | | ***Brassica nigra*** | | |
| --- | --- | --- | --- | --- | --- | --- | --- | --- | --- | --- | --- | --- | --- | --- | --- |
|  | ***Brassica rapa*** | | | ***Capsella bursa-pastoris*** | | | ***Descurainia sophia*** | | | ***Erysimum cheiranthoides*** | | | ***Rapistrum rugosum*** | | |
| **Species name** | **Contr. (%)** | **SD** | ***P*** | **Contr. (%)** | **SD** | ***P*** | **Contr. (%)** | **SD** | ***P*** | **Contr. (%)** | **SD** | ***P*** | **Contr. (%)** | **SD** | ***P*** |
| *Neocrepidodera transversa* | 3.37 | 0.0351 | 0.9853 | 4.37 | 0.0471 | 0.2087 | 4.08 | 0.0472 | 0.4620 | 4.76 | 0.0456 | **0.0273** | 3.37 | 0.0347 | 0.9875 |
| *Phaedon cochleariae* | 1.26 | 0.0248 | **0.0144** | 1.23 | 0.0280 | **0.0421** | 1.23 | 0.0282 | **0.0219** | 1.37 | 0.0292 | **0.0052** | 1.12 | 0.0236 | **0.0398** |
| *Phyllotreta atra* | 0.90 | 0.0217 | 0.8736 | 0.85 | 0.0247 | 0.8591 | 0.66 | 0.0202 | 0.9546 | 0.39 | 0.0149 | 0.9929 | 0.67 | 0.0181 | 0.9545 |
| *Phyllotreta undulata* | 2.96 | 0.0367 | 0.8919 | 4.91 | 0.0431 | **0.0024** | 5.13 | 0.0418 | **0.0001** | 4.67 | 0.0416 | **0.0014** | 3.30 | 0.0347 | 0.6805 |
| *Meligethes aeneus* | 3.46 | 0.0315 | 0.6799 | 4.40 | 0.0400 | 0.0813 | 4.42 | 0.0402 | 0.0509 | 4.23 | 0.0384 | 0.0918 | 3.25 | 0.0361 | 0.8028 |
| *Murgantia histrionica* | 0.39 | 0.0152 | 0.2626 | 0.24 | 0.0129 | 0.4180 | 0.24 | 0.0129 | 0.3806 | 0.23 | 0.0124 | 0.4011 | 0.19 | 0.0103 | 0.5015 |
| *Athalia rosae* | 0.41 | 0.0154 | 0.7959 | 0.52 | 0.0195 | 0.6506 | 0.52 | 0.0196 | 0.6886 | 0.50 | 0.0187 | 0.7014 | 0.40 | 0.0152 | 0.8046 |
| *Athalia* spp. | 0.00 | 0.0000 | 1.0000 | 0.00 | 0.0000 | 1.0000 | 0.83 | 0.0241 | 0.2353 | 0.00 | 0.0000 | 1.0000 | 0.00 | 0.0000 | 1.0000 |
| Loopers | 0.20 | 0.0110 | 0.9252 | 0.26 | 0.0139 | 0.8562 | 0.26 | 0.0139 | 0.8749 | 0.25 | 0.0134 | 0.8890 | 0.20 | 0.0109 | 0.9260 |
| *Evergestis extimalis* | 0.37 | 0.0142 | 0.0697 | 0.46 | 0.0178 | 0.0357 | 0.46 | 0.0179 | **0.0231** | 0.45 | 0.0171 | **0.0235** | 0.37 | 0.0141 | 0.0737 |
| *Autographa gamma* | 0.71 | 0.0188 | 0.6222 | 0.66 | 0.0200 | 0.6401 | 1.72 | 0.0338 | **0.0279** | 0.64 | 0.0194 | 0.6724 | 0.88 | 0.0211 | 0.4718 |
| *Mamestra brassicae* | 0.61 | 0.0190 | 0.9489 | 0.98 | 0.0282 | 0.7910 | 2.65 | 0.0401 | **0.0384** | 0.53 | 0.0205 | 0.9577 | 0.60 | 0.0188 | 0.9493 |
| *Pieris brassicae* | 0.23 | 0.0126 | 0.4611 | 0.30 | 0.0165 | 0.3906 | 0.30 | 0.0165 | 0.3635 | 0.29 | 0.0157 | 0.3802 | 0.23 | 0.0124 | 0.4584 |
| *Pieris rapae* | 3.22 | 0.0357 | 0.9572 | 5.02 | 0.0413 | **0.0022** | 5.02 | 0.0418 | **0.0005** | 4.68 | 0.0409 | **0.0036** | 3.25 | 0.0348 | 0.9542 |
| *Plutella xylostella* | 0.00 | 0.0000 | 1.0000 | 0.94 | 0.0274 | 0.0811 | 0.00 | 0.0000 | 1.0000 | 0.00 | 0.0000 | 1.0000 | 0.00 | 0.0000 | 1.0000 |
| Cicades | 1.42 | 0.0262 | 0.7531 | 1.11 | 0.0279 | 0.8746 | 1.43 | 0.0303 | 0.7343 | 0.68 | 0.0205 | 0.9913 | 1.25 | 0.0250 | 0.8469 |
| *Aleyrodes proletella* | 0.00 | 0.0000 | 1.0000 | 0.00 | 0.0000 | 1.0000 | 0.00 | 0.0000 | 1.0000 | 0.00 | 0.0000 | 1.0000 | 0.00 | 0.0000 | 1.0000 |
| *Aphis fabae* | 0.00 | 0.0000 | 1.0000 | 0.89 | 0.0260 | **0.0492** | 0.00 | 0.0000 | 1.0000 | 0.00 | 0.0000 | 1.0000 | 0.00 | 0.0000 | 1.0000 |
| *Brevicoryne brassicae* | 3.54 | 0.0368 | **0.0128** | 3.89 | 0.0403 | **0.0099** | 4.03 | 0.0420 | **0.0011** | 3.74 | 0.0387 | **0.0050** | 3.14 | 0.0324 | 0.0825 |
| *Myzus persicae* | 0.00 | 0.0000 | 1.0000 | 1.09 | 0.0327 | 0.9286 | 3.35 | 0.0469 | 0.0936 | 4.99 | 0.0476 | **0.0001** | 0.00 | 0.0000 | 1.0000 |
| *Myzus persicae* subsp. *Nicotiana* | 2.21 | 0.0335 | 0.0621 | 1.95 | 0.0364 | 0.2084 | 1.91 | 0.0368 | 0.1988 | 1.63 | 0.0334 | 0.3938 | 1.66 | 0.0286 | 0.3658 |
| *Lygus* spp. | 3.47 | 0.0352 | 0.9592 | 3.43 | 0.0399 | 0.9391 | 4.52 | 0.0468 | **0.0454** | 3.51 | 0.0397 | 0.9489 | 3.43 | 0.0348 | 0.9686 |
| Leaf mining flies | 1.70 | 0.0341 | 0.8639 | 3.32 | 0.0492 | 0.1707 | 4.94 | 0.0466 | **0.0007** | 2.35 | 0.0456 | 0.5872 | 1.35 | 0.0320 | 0.9445 |
| Weevils | 2.21 | 0.0308 | 0.7832 | 3.82 | 0.0437 | **0.0270** | 1.45 | 0.0313 | 0.9801 | 2.56 | 0.0382 | 0.5571 | 1.40 | 0.0261 | 0.9847 |
| *Dasineura sisymbrii* | 0.00 | 0.0000 | 1.0000 | 0.00 | 0.0000 | 1.0000 | 0.00 | 0.0000 | 1.0000 | 0.00 | 0.0000 | 1.0000 | 0.00 | 0.0000 | 1.0000 |
| Thrips | 4.25 | 0.0331 | 0.2385 | 6.29 | 0.0373 | **0.0001** | 5.93 | 0.0407 | **0.0001** | 4.33 | 0.0451 | 0.1648 | 1.74 | 0.0339 | 1.0000 |

**Table S18. Continued**

|  | ***Brassica nigra*** | | | ***Brassica nigra*** | | | ***Brassica nigra*** | | | ***Camelina sativa*** | | | ***Camelina sativa*** | | |
| --- | --- | --- | --- | --- | --- | --- | --- | --- | --- | --- | --- | --- | --- | --- | --- |
|  | ***Raphanus raphanistrum*** | | | ***Rorippa sylvestris*** | | | ***Sinapis alba*** | | | ***Capsella bursa-pastoris*** | | | ***Brassica rapa*** | | |
| **Species name** | **Contr. (%)** | **SD** | ***P*** | **Contr. (%)** | **SD** | ***P*** | **Contr. (%)** | **SD** | ***P*** | **Contr. (%)** | **SD** | ***P*** | **Contr. (%)** | **SD** | ***P*** |
| *Neocrepidodera transversa* | 3.24 | 0.0335 | 0.9952 | 3.00 | 0.0333 | 0.9989 | 3.27 | 0.0379 | 0.9934 | 5.93 | 0.0620 | **0.0007** | 4.19 | 0.0436 | 0.3386 |
| *Phaedon cochleariae* | 1.09 | 0.0230 | **0.0485** | 1.06 | 0.0224 | 0.0570 | 1.51 | 0.0279 | **0.0015** | 0.00 | 0.0000 | 1.0000 | 0.49 | 0.0184 | 0.5326 |
| *Phyllotreta atra* | 0.82 | 0.0195 | 0.9085 | 1.32 | 0.0239 | 0.5739 | 0.98 | 0.0237 | 0.8317 | 1.79 | 0.0429 | 0.2254 | 1.52 | 0.0321 | 0.3838 |
| *Phyllotreta undulata* | 3.18 | 0.0336 | 0.7796 | 3.51 | 0.0327 | 0.4560 | 4.19 | 0.0357 | **0.0164** | 2.64 | 0.0480 | 0.9377 | 6.18 | 0.0394 | **0.0001** |
| *Meligethes aeneus* | 3.10 | 0.0354 | 0.8587 | 3.31 | 0.0306 | 0.7668 | 3.58 | 0.0424 | 0.5885 | 0.41 | 0.0213 | 0.9999 | 0.51 | 0.0192 | 1.0000 |
| *Murgantia histrionica* | 0.50 | 0.0154 | 0.2054 | 0.18 | 0.0100 | 0.5438 | 0.21 | 0.0111 | 0.4612 | 0.00 | 0.0000 | 1.0000 | 0.26 | 0.0143 | 0.3455 |
| *Athalia rosae* | 0.39 | 0.0148 | 0.8225 | 2.68 | 0.0313 | **0.0002** | 1.22 | 0.0265 | 0.1882 | 0.00 | 0.0000 | 1.0000 | 0.00 | 0.0000 | 1.0000 |
| *Athalia* spp. | 0.19 | 0.0103 | 0.8361 | 1.42 | 0.0267 | **0.0179** | 0.00 | 0.0000 | 1.0000 | 0.00 | 0.0000 | 1.0000 | 0.00 | 0.0000 | 1.0000 |
| Loopers | 0.36 | 0.0140 | 0.8842 | 4.20 | 0.0305 | **0.0001** | 0.22 | 0.0119 | 0.9091 | 0.00 | 0.0000 | 1.0000 | 0.00 | 0.0000 | 1.0000 |
| *Evergestis extimalis* | 0.36 | 0.0136 | 0.0762 | 0.36 | 0.0137 | 0.0748 | 0.40 | 0.0153 | **0.0447** | 0.00 | 0.0000 | 1.0000 | 0.00 | 0.0000 | 1.0000 |
| *Autographa gamma* | 1.13 | 0.0224 | 0.2574 | 0.69 | 0.0184 | 0.6369 | 0.58 | 0.0175 | 0.7528 | 0.00 | 0.0000 | 1.0000 | 0.24 | 0.0132 | 0.9139 |
| *Mamestra brassicae* | 0.77 | 0.0212 | 0.9136 | 3.44 | 0.0326 | **0.0009** | 0.47 | 0.0179 | 0.9711 | 0.65 | 0.0270 | 0.9035 | 0.24 | 0.0132 | 0.9879 |
| *Pieris brassicae* | 0.40 | 0.0154 | 0.3304 | 0.22 | 0.0121 | 0.4558 | 0.65 | 0.0205 | 0.0913 | 0.00 | 0.0000 | 1.0000 | 0.00 | 0.0000 | 1.0000 |
| *Pieris rapae* | 3.25 | 0.0333 | 0.9562 | 3.23 | 0.0338 | 0.9623 | 3.90 | 0.0370 | 0.3012 | 1.75 | 0.0424 | 0.9996 | 4.76 | 0.0396 | **0.0030** |
| *Plutella xylostella* | 0.00 | 0.0000 | 1.0000 | 0.00 | 0.0000 | 1.0000 | 0.00 | 0.0000 | 1.0000 | 3.19 | 0.0560 | **0.0001** | 1.71 | 0.0364 | **0.0004** |
| Cicades | 1.20 | 0.0239 | 0.8799 | 1.39 | 0.0258 | 0.7685 | 1.50 | 0.0278 | 0.6743 | 1.32 | 0.0360 | 0.7653 | 1.64 | 0.0317 | 0.5574 |
| *Aleyrodes proletella* | 0.00 | 0.0000 | 1.0000 | 0.00 | 0.0000 | 1.0000 | 0.00 | 0.0000 | 1.0000 | 0.00 | 0.0000 | 1.0000 | 0.00 | 0.0000 | 1.0000 |
| *Aphis fabae* | 0.00 | 0.0000 | 1.0000 | 0.00 | 0.0000 | 1.0000 | 0.00 | 0.0000 | 1.0000 | 1.16 | 0.0331 | **0.0001** | 0.00 | 0.0000 | 1.0000 |
| *Brevicoryne brassicae* | 3.08 | 0.0320 | 0.1064 | 2.94 | 0.0305 | 0.1653 | 3.31 | 0.0341 | **0.0437** | 0.00 | 0.0000 | 1.0000 | 6.75 | 0.0325 | **0.0001** |
| *Myzus persicae* | 0.25 | 0.0136 | 0.9987 | 3.21 | 0.0361 | 0.1209 | 0.00 | 0.0000 | 1.0000 | 3.73 | 0.0608 | 0.0601 | 2.07 | 0.0395 | 0.6430 |
| *Myzus persicae* subsp. *Nicotiana* | 1.86 | 0.0291 | 0.2239 | 1.39 | 0.0268 | 0.5915 | 2.99 | 0.0365 | **0.0003** | 1.21 | 0.0328 | 0.6870 | 2.18 | 0.0393 | 0.0802 |
| *Lygus* spp. | 3.50 | 0.0340 | 0.9480 | 3.90 | 0.0365 | 0.5467 | 3.18 | 0.0353 | 0.9931 | 1.18 | 0.0322 | 1.0000 | 4.74 | 0.0411 | **0.0186** |
| Leaf mining flies | 1.54 | 0.0316 | 0.9034 | 3.15 | 0.0346 | 0.1890 | 1.35 | 0.0356 | 0.9407 | 3.82 | 0.0611 | 0.0638 | 1.26 | 0.0330 | 0.9521 |
| Weevils | 2.36 | 0.0295 | 0.6917 | 1.31 | 0.0245 | 0.9905 | 1.16 | 0.0250 | 0.9964 | 5.80 | 0.0595 | **0.0001** | 4.15 | 0.0419 | **0.0023** |
| *Dasineura sisymbrii* | 0.00 | 0.0000 | 1.0000 | 6.06 | 0.0221 | **0.0001** | 0.00 | 0.0000 | 1.0000 | 0.00 | 0.0000 | 1.0000 | 0.00 | 0.0000 | 1.0000 |
| Thrips | 2.16 | 0.0340 | 1.0000 | 2.75 | 0.0350 | 0.9997 | 3.47 | 0.0393 | 0.9730 | 5.74 | 0.0574 | **0.0013** | 4.17 | 0.0419 | 0.3319 |

**Table S18. Continued**

|  | ***Camelina sativa*** | | | ***Brassica nigra*** | | | ***Capsella bursa-pastoris*** | | | ***Camelina sativa*** | | | ***Camelina sativa*** | | |
| --- | --- | --- | --- | --- | --- | --- | --- | --- | --- | --- | --- | --- | --- | --- | --- |
|  | ***Brassica nigra*** | | | ***Sinapis arvensis*** | | | ***Brassica rapa*** | | | ***Sinapis arvensis*** | | | ***Sinapis alba*** | | |
| **Species name** | **Contr. (%)** | **SD** | ***P*** | **Contr. (%)** | **SD** | ***P*** | **Contr. (%)** | **SD** | ***P*** | **Contr. (%)** | **SD** | ***P*** | **Contr. (%)** | **SD** | ***P*** |
| *Neocrepidodera transversa* | 4.24 | 0.0447 | 0.2930 | 3.79 | 0.0406 | 0.8023 | 4.42 | 0.0456 | 0.1735 | 5.16 | 0.0525 | **0.0039** | 5.12 | 0.0490 | **0.0049** |
| *Phaedon cochleariae* | 1.17 | 0.0267 | **0.0310** | 1.11 | 0.0252 | **0.0412** | 0.51 | 0.0193 | 0.5266 | 0.00 | 0.0000 | 1.0000 | 0.80 | 0.0241 | 0.2304 |
| *Phyllotreta atra* | 1.22 | 0.0290 | 0.6489 | 1.64 | 0.0301 | 0.2864 | 1.22 | 0.0300 | 0.6289 | 2.47 | 0.0420 | **0.0068** | 1.69 | 0.0358 | 0.2532 |
| *Phyllotreta undulata* | 4.74 | 0.0402 | **0.0007** | 4.48 | 0.0374 | **0.0022** | 6.29 | 0.0436 | **0.0001** | 1.79 | 0.0377 | 0.9992 | 1.85 | 0.0360 | 0.9989 |
| *Meligethes aeneus* | 4.18 | 0.0383 | 0.1207 | 3.84 | 0.0446 | 0.3570 | 0.24 | 0.0129 | 0.9999 | 8.31 | 0.0397 | **0.0001** | 8.56 | 0.0278 | **0.0001** |
| *Murgantia histrionica* | 0.23 | 0.0123 | 0.4110 | 0.22 | 0.0117 | 0.4142 | 0.28 | 0.0150 | 0.3987 | 0.00 | 0.0000 | 1.0000 | 0.00 | 0.0000 | 1.0000 |
| *Athalia rosae* | 0.49 | 0.0185 | 0.7010 | 0.46 | 0.0175 | 0.7332 | 0.00 | 0.0000 | 1.0000 | 0.00 | 0.0000 | 1.0000 | 1.10 | 0.0287 | 0.2610 |
| *Athalia* spp. | 0.00 | 0.0000 | 1.0000 | 0.00 | 0.0000 | 1.0000 | 0.00 | 0.0000 | 1.0000 | 0.00 | 0.0000 | 1.0000 | 0.00 | 0.0000 | 1.0000 |
| Loopers | 0.24 | 0.0132 | 0.8836 | 0.23 | 0.0125 | 0.8928 | 0.00 | 0.0000 | 1.0000 | 0.00 | 0.0000 | 1.0000 | 0.00 | 0.0000 | 1.0000 |
| *Evergestis extimalis* | 0.44 | 0.0170 | **0.0312** | 0.42 | 0.0161 | **0.0340** | 0.00 | 0.0000 | 1.0000 | 0.00 | 0.0000 | 1.0000 | 0.00 | 0.0000 | 1.0000 |
| *Autographa gamma* | 0.63 | 0.0192 | 0.6834 | 0.60 | 0.0183 | 0.7183 | 0.26 | 0.0139 | 0.8931 | 0.00 | 0.0000 | 1.0000 | 0.00 | 0.0000 | 1.0000 |
| *Mamestra brassicae* | 0.53 | 0.0202 | 0.9566 | 0.50 | 0.0190 | 0.9639 | 0.72 | 0.0239 | 0.8800 | 0.00 | 0.0000 | 1.0000 | 0.00 | 0.0000 | 1.0000 |
| *Pieris brassicae* | 0.29 | 0.0155 | 0.3688 | 0.27 | 0.0146 | 0.3790 | 0.00 | 0.0000 | 1.0000 | 0.00 | 0.0000 | 1.0000 | 0.53 | 0.0201 | 0.1642 |
| *Pieris rapae* | 4.70 | 0.0398 | **0.0034** | 4.13 | 0.0394 | 0.0842 | 5.09 | 0.0412 | **0.0018** | 3.53 | 0.0473 | 0.7975 | 3.20 | 0.0428 | 0.9583 |
| *Plutella xylostella* | 1.70 | 0.0372 | **0.0009** | 0.00 | 0.0000 | 1.0000 | 0.94 | 0.0270 | 0.0845 | 2.07 | 0.0441 | **0.0001** | 1.91 | 0.0408 | **0.0001** |
| Cicades | 1.13 | 0.0265 | 0.8976 | 1.11 | 0.0270 | 0.9136 | 1.66 | 0.0334 | 0.5353 | 1.30 | 0.0341 | 0.8224 | 1.77 | 0.0342 | 0.4497 |
| *Aleyrodes proletella* | 0.00 | 0.0000 | 1.0000 | 0.00 | 0.0000 | 1.0000 | 0.00 | 0.0000 | 1.0000 | 0.00 | 0.0000 | 1.0000 | 0.00 | 0.0000 | 1.0000 |
| *Aphis fabae* | 0.00 | 0.0000 | 1.0000 | 0.00 | 0.0000 | 1.0000 | 0.90 | 0.0258 | **0.0487** | 0.00 | 0.0000 | 1.0000 | 0.00 | 0.0000 | 1.0000 |
| *Brevicoryne brassicae* | 3.70 | 0.0382 | **0.0074** | 3.49 | 0.0360 | **0.0162** | 7.12 | 0.0346 | **0.0001** | 0.00 | 0.0000 | 1.0000 | 0.00 | 0.0000 | 1.0000 |
| *Myzus persicae* | 2.06 | 0.0406 | 0.6608 | 0.25 | 0.0136 | 0.9991 | 1.09 | 0.0316 | 0.9299 | 2.68 | 0.0486 | 0.3316 | 2.33 | 0.0445 | 0.5183 |
| *Myzus persicae* subsp. *Nicotiana* | 1.95 | 0.0352 | 0.1739 | 1.68 | 0.0323 | 0.3532 | 2.22 | 0.0414 | 0.0970 | 0.96 | 0.0288 | 0.8704 | 3.40 | 0.0436 | **0.0001** |
| *Lygus* spp. | 3.28 | 0.0377 | 0.9839 | 3.09 | 0.0355 | 0.9964 | 5.02 | 0.0434 | **0.0108** | 1.06 | 0.0276 | 1.0000 | 2.29 | 0.0379 | 1.0000 |
| Leaf mining flies | 1.81 | 0.0433 | 0.8239 | 2.30 | 0.0413 | 0.6102 | 3.10 | 0.0459 | 0.2437 | 2.01 | 0.0420 | 0.7419 | 0.37 | 0.0189 | 0.9989 |
| Weevils | 4.10 | 0.0422 | **0.0025** | 1.94 | 0.0320 | 0.8880 | 4.09 | 0.0438 | **0.0092** | 4.91 | 0.0492 | **0.0001** | 4.56 | 0.0455 | **0.0002** |
| *Dasineura sisymbrii* | 0.00 | 0.0000 | 1.0000 | 0.00 | 0.0000 | 1.0000 | 0.00 | 0.0000 | 1.0000 | 0.00 | 0.0000 | 1.0000 | 0.00 | 0.0000 | 1.0000 |
| Thrips | 4.15 | 0.0446 | 0.3567 | 4.19 | 0.0406 | 0.2908 | 2.33 | 0.0368 | 0.9998 | 4.99 | 0.0510 | **0.0059** | 4.64 | 0.0480 | **0.0366** |

**Table S18. Continued**

|  | ***Camelina sativa*** | | | ***Camelina sativa*** | | | ***Camelina sativa*** | | | ***Camelina sativa*** | | | ***Camelina sativa*** | | |
| --- | --- | --- | --- | --- | --- | --- | --- | --- | --- | --- | --- | --- | --- | --- | --- |
|  | ***Rorippa sylvestris*** | | | ***Rapistrum rugosum*** | | | ***Raphanus raphanistrum*** | | | ***Erysimum cheiranthoides*** | | | ***Descurainia sophia*** | | |
| **Species name** | **Contr. (%)** | **sd** | **p** | **Contr. (%)** | **sd** | **p** | **Contr. (%)** | **sd** | **p** | **Contr. (%)** | **sd** | **p** | **Contr. (%)** | **sd** | **p** |
| *Neocrepidodera transversa* | 4.23 | 0.0424 | 0.3008 | 4.06 | 0.0424 | 0.4798 | 3.90 | 0.0410 | 0.6714 | 4.87 | 0.0563 | **0.0172** | 6.36 | 0.0626 | **0.0001** |
| *Phaedon cochleariae* | 0.20 | 0.0108 | 0.8964 | 0.26 | 0.0143 | 0.7730 | 0.26 | 0.0143 | 0.7698 | 0.35 | 0.0189 | 0.7369 | 0.00 | 0.0000 | 1.0000 |
| *Phyllotreta atra* | 1.90 | 0.0325 | 0.1261 | 1.26 | 0.0291 | 0.6162 | 1.38 | 0.0293 | 0.5096 | 1.21 | 0.0352 | 0.6507 | 1.56 | 0.0400 | 0.3615 |
| *Phyllotreta undulata* | 2.47 | 0.0353 | 0.9805 | 4.09 | 0.0398 | **0.0304** | 3.93 | 0.0386 | 0.0766 | 2.80 | 0.0475 | 0.9349 | 1.55 | 0.0395 | 0.9995 |
| *Meligethes aeneus* | 0.72 | 0.0221 | 0.9998 | 5.96 | 0.0363 | **0.0003** | 6.39 | 0.0302 | **0.0001** | 0.39 | 0.0202 | 1.0000 | 0.42 | 0.0215 | 0.9999 |
| *Murgantia histrionica* | 0.00 | 0.0000 | 1.0000 | 0.00 | 0.0000 | 1.0000 | 0.39 | 0.0146 | 0.2833 | 0.00 | 0.0000 | 1.0000 | 0.00 | 0.0000 | 1.0000 |
| *Athalia rosae* | 3.17 | 0.0374 | **0.0001** | 0.00 | 0.0000 | 1.0000 | 0.00 | 0.0000 | 1.0000 | 0.00 | 0.0000 | 1.0000 | 0.00 | 0.0000 | 1.0000 |
| *Athalia* spp. | 1.74 | 0.0325 | **0.0031** | 0.00 | 0.0000 | 1.0000 | 0.23 | 0.0123 | 0.7481 | 0.00 | 0.0000 | 1.0000 | 1.05 | 0.0302 | 0.1168 |
| Loopers | 5.22 | 0.0362 | **0.0001** | 0.00 | 0.0000 | 1.0000 | 0.21 | 0.0115 | 0.9134 | 0.00 | 0.0000 | 1.0000 | 0.00 | 0.0000 | 1.0000 |
| *Evergestis extimalis* | 0.00 | 0.0000 | 1.0000 | 0.00 | 0.0000 | 1.0000 | 0.00 | 0.0000 | 1.0000 | 0.00 | 0.0000 | 1.0000 | 0.00 | 0.0000 | 1.0000 |
| *Autographa gamma* | 0.24 | 0.0132 | 0.9155 | 0.51 | 0.0191 | 0.7810 | 0.87 | 0.0225 | 0.4637 | 0.00 | 0.0000 | 1.0000 | 1.64 | 0.0397 | **0.0484** |
| *Mamestra brassicae* | 4.25 | 0.0394 | **0.0001** | 0.24 | 0.0132 | 0.9874 | 0.51 | 0.0199 | 0.9590 | 0.00 | 0.0000 | 1.0000 | 3.11 | 0.0491 | **0.0071** |
| *Pieris brassicae* | 0.00 | 0.0000 | 1.0000 | 0.00 | 0.0000 | 1.0000 | 0.23 | 0.0123 | 0.4712 | 0.00 | 0.0000 | 1.0000 | 0.00 | 0.0000 | 1.0000 |
| *Pieris rapae* | 3.84 | 0.0399 | 0.3861 | 4.29 | 0.0394 | 0.0381 | 3.66 | 0.0399 | 0.6529 | 2.54 | 0.0465 | 0.9987 | 1.91 | 0.0441 | 1.0000 |
| *Plutella xylostella* | 1.61 | 0.0347 | **0.0008** | 1.67 | 0.0357 | **0.0004** | 1.60 | 0.0342 | **0.0013** | 2.32 | 0.0496 | **0.0001** | 2.49 | 0.0534 | **0.0001** |
| Cicades | 1.61 | 0.0312 | 0.6011 | 1.44 | 0.0302 | 0.7271 | 1.36 | 0.0287 | 0.7750 | 0.71 | 0.0256 | 0.9844 | 1.73 | 0.0389 | 0.4955 |
| *Aleyrodes proletella* | 0.00 | 0.0000 | 1.0000 | 0.00 | 0.0000 | 1.0000 | 0.00 | 0.0000 | 1.0000 | 0.00 | 0.0000 | 1.0000 | 0.00 | 0.0000 | 1.0000 |
| *Aphis fabae* | 0.00 | 0.0000 | 1.0000 | 0.00 | 0.0000 | 1.0000 | 0.00 | 0.0000 | 1.0000 | 0.00 | 0.0000 | 1.0000 | 0.00 | 0.0000 | 1.0000 |
| *Brevicoryne brassicae* | 0.00 | 0.0000 | 1.0000 | 1.59 | 0.0321 | 0.8949 | 2.26 | 0.0352 | 0.6036 | 0.00 | 0.0000 | 1.0000 | 1.52 | 0.0371 | 0.9144 |
| *Myzus persicae* | 3.98 | 0.0428 | **0.0121** | 2.03 | 0.0387 | 0.6688 | 2.10 | 0.0381 | 0.6333 | 6.05 | 0.0580 | **0.0001** | 5.22 | 0.0627 | **0.0001** |
| *Myzus persicae* subsp. *Nicotiana* | 0.76 | 0.0229 | 0.9362 | 1.17 | 0.0271 | 0.7526 | 1.58 | 0.0304 | 0.4319 | 0.71 | 0.0256 | 0.9509 | 1.13 | 0.0335 | 0.7586 |
| *Lygus* spp. | 7.46 | 0.0266 | **0.0001** | 4.70 | 0.0407 | **0.0199** | 5.53 | 0.0354 | **0.0006** | 2.12 | 0.0411 | 1.0000 | 6.40 | 0.0565 | **0.0001** |
| Leaf mining flies | 3.94 | 0.0439 | **0.0241** | 0.64 | 0.0241 | 0.9962 | 1.02 | 0.0269 | 0.9786 | 1.86 | 0.0436 | 0.7996 | 6.96 | 0.0616 | **0.0001** |
| Weevils | 3.92 | 0.0397 | **0.0072** | 4.07 | 0.0406 | **0.0026** | 3.91 | 0.0398 | **0.0083** | 5.44 | 0.0551 | **0.0001** | 5.72 | 0.0575 | **0.0001** |
| *Dasineura sisymbrii* | 7.51 | 0.0259 | **0.0001** | 0.00 | 0.0000 | 1.0000 | 0.00 | 0.0000 | 1.0000 | 0.00 | 0.0000 | 1.0000 | 0.00 | 0.0000 | 1.0000 |
| Thrips | 3.97 | 0.0416 | 0.6001 | 4.13 | 0.0440 | 0.3734 | 3.95 | 0.0417 | 0.6217 | 5.50 | 0.0563 | **0.0004** | 5.80 | 0.0586 | **0.0001** |

**Table S18. Continued**

|  | ***Descurainia sophia*** | | | ***Descurainia sophia*** | | | ***Descurainia sophia*** | | | ***Descurainia sophia*** | | | ***Descurainia sophia*** | | |
| --- | --- | --- | --- | --- | --- | --- | --- | --- | --- | --- | --- | --- | --- | --- | --- |
|  | ***Sinapis alba*** | | | ***Rorippa sylvestris*** | | | ***Rapistrum rugosum*** | | | ***Raphanus raphanistrum*** | | | ***Erysimum cheiranthoides*** | | |
| **Species name** | **Contr. (%)** | **SD** | ***P*** | **Contr. (%)** | **SD** | ***P*** | **Contr. (%)** | **SD** | ***P*** | **Contr. (%)** | **SD** | ***P*** | **Contr. (%)** | **SD** | ***P*** |
| *Neocrepidodera transversa* | 2.85 | 0.0453 | 0.9999 | 3.04 | 0.0396 | 0.9983 | 4.65 | 0.0450 | 0.0533 | 4.41 | 0.0431 | 0.1456 | 9.08 | 0.0557 | **0.0001** |
| *Phaedon cochleariae* | 0.84 | 0.0255 | 0.1999 | 0.21 | 0.0113 | 0.8860 | 0.28 | 0.0151 | 0.7593 | 0.28 | 0.0151 | 0.7627 | 0.37 | 0.0204 | 0.7344 |
| *Phyllotreta atra* | 1.17 | 0.0306 | 0.6943 | 1.56 | 0.0297 | 0.3621 | 0.72 | 0.0219 | 0.9381 | 0.91 | 0.0239 | 0.8622 | 0.35 | 0.0182 | 0.9955 |
| *Phyllotreta undulata* | 1.36 | 0.0315 | 1.0000 | 2.29 | 0.0347 | 0.9925 | 4.31 | 0.0415 | **0.0094** | 4.14 | 0.0403 | **0.0232** | 2.42 | 0.0463 | 0.9851 |
| *Meligethes aeneus* | 9.48 | 0.0254 | **0.0001** | 0.50 | 0.0187 | 1.0000 | 6.46 | 0.0377 | **0.0001** | 6.95 | 0.0303 | **0.0001** | 0.00 | 0.0000 | 1.0000 |
| *Murgantia histrionica* | 0.00 | 0.0000 | 1.0000 | 0.00 | 0.0000 | 1.0000 | 0.00 | 0.0000 | 1.0000 | 0.40 | 0.0152 | 0.2587 | 0.00 | 0.0000 | 1.0000 |
| *Athalia rosae* | 1.17 | 0.0306 | 0.2202 | 3.35 | 0.0396 | **0.0001** | 0.00 | 0.0000 | 1.0000 | 0.00 | 0.0000 | 1.0000 | 0.00 | 0.0000 | 1.0000 |
| *Athalia* spp. | 0.92 | 0.0263 | 0.1806 | 2.28 | 0.0367 | **0.0001** | 0.82 | 0.0236 | 0.2395 | 0.98 | 0.0250 | 0.1470 | 1.07 | 0.0306 | 0.1029 |
| Loopers | 0.00 | 0.0000 | 1.0000 | 5.51 | 0.0385 | **0.0001** | 0.00 | 0.0000 | 1.0000 | 0.22 | 0.0120 | 0.8996 | 0.00 | 0.0000 | 1.0000 |
| *Evergestis extimalis* | 0.00 | 0.0000 | 1.0000 | 0.00 | 0.0000 | 1.0000 | 0.00 | 0.0000 | 1.0000 | 0.00 | 0.0000 | 1.0000 | 0.00 | 0.0000 | 1.0000 |
| *Autographa gamma* | 1.40 | 0.0340 | 0.1146 | 1.38 | 0.0309 | 0.1194 | 1.62 | 0.0333 | **0.0474** | 1.84 | 0.0332 | **0.0148** | 1.66 | 0.0405 | **0.0375** |
| *Mamestra brassicae* | 2.66 | 0.0421 | **0.0355** | 4.33 | 0.0433 | **0.0001** | 2.48 | 0.0379 | 0.0706 | 2.52 | 0.0376 | 0.0663 | 3.16 | 0.0501 | **0.0031** |
| *Pieris brassicae* | 0.56 | 0.0213 | 0.1472 | 0.00 | 0.0000 | 1.0000 | 0.00 | 0.0000 | 1.0000 | 0.24 | 0.0129 | 0.4423 | 0.00 | 0.0000 | 1.0000 |
| *Pieris rapae* | 3.27 | 0.0449 | 0.9426 | 4.05 | 0.0423 | 0.1428 | 4.54 | 0.0415 | 0.0076 | 3.85 | 0.0423 | 0.3822 | 2.46 | 0.0483 | 0.9986 |
| *Plutella xylostella* | 0.00 | 0.0000 | 1.0000 | 0.00 | 0.0000 | 1.0000 | 0.00 | 0.0000 | 1.0000 | 0.00 | 0.0000 | 1.0000 | 0.00 | 0.0000 | 1.0000 |
| Cicades | 2.10 | 0.0379 | 0.1999 | 1.89 | 0.0342 | 0.3558 | 1.73 | 0.0335 | 0.4878 | 1.64 | 0.0318 | 0.5661 | 1.14 | 0.0326 | 0.8943 |
| *Aleyrodes proletella* | 0.00 | 0.0000 | 1.0000 | 0.00 | 0.0000 | 1.0000 | 0.00 | 0.0000 | 1.0000 | 0.00 | 0.0000 | 1.0000 | 0.00 | 0.0000 | 1.0000 |
| *Aphis fabae* | 0.00 | 0.0000 | 1.0000 | 0.00 | 0.0000 | 1.0000 | 0.00 | 0.0000 | 1.0000 | 0.00 | 0.0000 | 1.0000 | 0.00 | 0.0000 | 1.0000 |
| *Brevicoryne brassicae* | 1.31 | 0.0320 | 0.9569 | 1.13 | 0.0277 | 0.9762 | 2.40 | 0.0382 | 0.5089 | 2.86 | 0.0392 | 0.2166 | 1.55 | 0.0377 | 0.9115 |
| *Myzus persicae* | 3.77 | 0.0511 | **0.0249** | 4.23 | 0.0451 | **0.0052** | 3.30 | 0.0444 | 0.1034 | 3.23 | 0.0430 | 0.1238 | 6.31 | 0.0638 | **0.0001** |
| *Myzus persicae* subsp. *Nicotiana* | 3.54 | 0.0465 | **0.0001** | 0.57 | 0.0214 | 0.9721 | 1.02 | 0.0267 | 0.8294 | 1.49 | 0.0311 | 0.5046 | 0.44 | 0.0227 | 0.9900 |
| *Lygus* spp. | 5.28 | 0.0495 | **0.0021** | 3.71 | 0.0467 | 0.7993 | 4.27 | 0.0457 | 0.1436 | 3.93 | 0.0446 | 0.5200 | 6.40 | 0.0588 | **0.0001** |
| Leaf mining flies | 5.94 | 0.0518 | **0.0001** | 4.22 | 0.0437 | **0.0096** | 5.14 | 0.0449 | **0.0005** | 4.82 | 0.0437 | **0.0006** | 6.86 | 0.0632 | **0.0001** |
| Weevils | 1.18 | 0.0307 | 0.9945 | 1.40 | 0.0300 | 0.9829 | 1.54 | 0.0325 | 0.9684 | 2.90 | 0.0372 | 0.3196 | 3.35 | 0.0524 | 0.0933 |
| *Dasineura sisymbrii* | 0.00 | 0.0000 | 1.0000 | 7.97 | 0.0284 | **0.0001** | 0.00 | 0.0000 | 1.0000 | 0.00 | 0.0000 | 1.0000 | 0.00 | 0.0000 | 1.0000 |
| Thrips | 5.26 | 0.0497 | **0.0014** | 4.93 | 0.0410 | **0.0078** | 7.17 | 0.0355 | **0.0001** | 5.95 | 0.0382 | **0.0001** | 5.55 | 0.0573 | **0.0006** |

**Table S18. Continued**

|  | ***Descurainia sophia*** | | | ***Descurainia sophia*** | | | ***Capsella bursa-pastoris*** | | | ***Capsella bursa-pastoris*** | | | ***Capsella bursa-pastoris*** | | |
| --- | --- | --- | --- | --- | --- | --- | --- | --- | --- | --- | --- | --- | --- | --- | --- |
|  | ***Capsella bursa-pastoris*** | | | ***Brassica rapa*** | | | ***Sinapis arvensis*** | | | ***Sinapis alba*** | | | ***Rapistrum rugosum*** | | |
| **Species name** | **Contr. (%)** | **SD** | ***P*** | **Contr. (%)** | **SD** | ***P*** | **Contr. (%)** | **SD** | ***P*** | **Contr. (%)** | **SD** | ***P*** | **Contr. (%)** | **SD** | ***P*** |
| *Neocrepidodera transversa* | 6.13 | 0.0648 | **0.0004** | 4.48 | 0.0448 | 0.1177 | 5.26 | 0.0541 | **0.0080** | 4.71 | 0.0489 | 0.0622 | 4.37 | 0.0452 | 0.2107 |
| *Phaedon cochleariae* | 0.00 | 0.0000 | 1.0000 | 0.51 | 0.0194 | 0.5180 | 0.00 | 0.0000 | 1.0000 | 0.84 | 0.0254 | 0.2214 | 0.28 | 0.0150 | 0.7456 |
| *Phyllotreta atra* | 1.04 | 0.0339 | 0.7561 | 1.05 | 0.0274 | 0.7830 | 2.26 | 0.0417 | **0.0494** | 1.36 | 0.0336 | 0.5241 | 0.91 | 0.0255 | 0.8272 |
| *Phyllotreta undulata* | 2.15 | 0.0450 | 0.9871 | 6.99 | 0.0403 | **0.0001** | 2.27 | 0.0418 | 0.9848 | 2.28 | 0.0396 | 0.9835 | 4.30 | 0.0421 | **0.0213** |
| *Meligethes aeneus* | 0.00 | 0.0000 | 1.0000 | 0.24 | 0.0129 | 1.0000 | 9.14 | 0.0405 | **0.0001** | 9.42 | 0.0249 | **0.0001** | 6.43 | 0.0374 | **0.0002** |
| *Murgantia histrionica* | 0.00 | 0.0000 | 1.0000 | 0.28 | 0.0151 | 0.3282 | 0.00 | 0.0000 | 1.0000 | 0.00 | 0.0000 | 1.0000 | 0.00 | 0.0000 | 1.0000 |
| *Athalia rosae* | 0.00 | 0.0000 | 1.0000 | 0.00 | 0.0000 | 1.0000 | 0.00 | 0.0000 | 1.0000 | 1.17 | 0.0304 | 0.2557 | 0.00 | 0.0000 | 1.0000 |
| *Athalia* spp. | 1.12 | 0.0322 | 0.1259 | 0.84 | 0.0240 | 0.2262 | 0.00 | 0.0000 | 1.0000 | 0.00 | 0.0000 | 1.0000 | 0.00 | 0.0000 | 1.0000 |
| Loopers | 0.00 | 0.0000 | 1.0000 | 0.00 | 0.0000 | 1.0000 | 0.00 | 0.0000 | 1.0000 | 0.00 | 0.0000 | 1.0000 | 0.00 | 0.0000 | 1.0000 |
| *Evergestis extimalis* | 0.00 | 0.0000 | 1.0000 | 0.00 | 0.0000 | 1.0000 | 0.00 | 0.0000 | 1.0000 | 0.00 | 0.0000 | 1.0000 | 0.00 | 0.0000 | 1.0000 |
| *Autographa gamma* | 1.76 | 0.0429 | **0.0493** | 1.44 | 0.0321 | 0.0977 | 0.00 | 0.0000 | 1.0000 | 0.00 | 0.0000 | 1.0000 | 0.53 | 0.0201 | 0.7297 |
| *Mamestra brassicae* | 3.67 | 0.0554 | **0.0018** | 2.53 | 0.0385 | 0.0634 | 0.59 | 0.0247 | 0.9178 | 0.55 | 0.0229 | 0.9301 | 0.71 | 0.0236 | 0.8815 |
| *Pieris brassicae* | 0.00 | 0.0000 | 1.0000 | 0.00 | 0.0000 | 1.0000 | 0.00 | 0.0000 | 1.0000 | 0.56 | 0.0212 | 0.1820 | 0.00 | 0.0000 | 1.0000 |
| *Pieris rapae* | 1.51 | 0.0418 | 1.0000 | 5.09 | 0.0417 | **0.0009** | 3.56 | 0.0496 | 0.7362 | 3.19 | 0.0443 | 0.9390 | 4.53 | 0.0411 | **0.0182** |
| *Plutella xylostella* | 1.33 | 0.0382 | **0.0222** | 0.00 | 0.0000 | 1.0000 | 1.13 | 0.0323 | **0.0470** | 1.05 | 0.0300 | 0.0661 | 0.93 | 0.0265 | 0.0906 |
| Cicades | 1.77 | 0.0422 | 0.4581 | 1.94 | 0.0349 | 0.3144 | 1.30 | 0.0366 | 0.7825 | 1.80 | 0.0362 | 0.4330 | 1.44 | 0.0318 | 0.6968 |
| *Aleyrodes proletella* | 0.00 | 0.0000 | 1.0000 | 0.00 | 0.0000 | 1.0000 | 0.00 | 0.0000 | 1.0000 | 0.00 | 0.0000 | 1.0000 | 0.00 | 0.0000 | 1.0000 |
| *Aphis fabae* | 1.25 | 0.0357 | **0.0001** | 0.00 | 0.0000 | 1.0000 | 1.07 | 0.0305 | **0.0039** | 1.00 | 0.0285 | **0.0260** | 0.89 | 0.0254 | **0.0498** |
| *Brevicoryne brassicae* | 1.63 | 0.0399 | 0.8490 | 6.43 | 0.0408 | **0.0001** | 0.00 | 0.0000 | 1.0000 | 0.00 | 0.0000 | 1.0000 | 1.67 | 0.0338 | 0.8419 |
| *Myzus persicae* | 5.28 | 0.0680 | **0.0008** | 3.36 | 0.0453 | 0.0913 | 1.60 | 0.0412 | 0.8007 | 1.24 | 0.0359 | 0.8986 | 1.07 | 0.0310 | 0.9333 |
| *Myzus persicae* subsp. *Nicotiana* | 0.99 | 0.0322 | 0.8058 | 2.18 | 0.0418 | 0.0800 | 0.83 | 0.0275 | 0.8806 | 3.54 | 0.0460 | **0.0002** | 1.09 | 0.0267 | 0.7636 |
| *Lygus* spp. | 6.91 | 0.0612 | **0.0001** | 4.36 | 0.0466 | 0.0953 | 1.04 | 0.0292 | 1.0000 | 2.37 | 0.0403 | 0.9995 | 4.98 | 0.0429 | **0.0133** |
| Leaf mining flies | 6.87 | 0.0668 | **0.0001** | 5.10 | 0.0458 | **0.0004** | 4.02 | 0.0560 | **0.0377** | 3.02 | 0.0503 | 0.2675 | 2.77 | 0.0441 | 0.3777 |
| Weevils | 5.35 | 0.0606 | **0.0001** | 2.71 | 0.0395 | 0.4486 | 4.72 | 0.0515 | **0.0006** | 4.19 | 0.0473 | **0.0065** | 3.81 | 0.0423 | **0.0303** |
| *Dasineura sisymbrii* | 0.00 | 0.0000 | 1.0000 | 0.00 | 0.0000 | 1.0000 | 0.00 | 0.0000 | 1.0000 | 0.00 | 0.0000 | 1.0000 | 0.00 | 0.0000 | 1.0000 |
| Thrips | 2.34 | 0.0500 | 0.9998 | 2.77 | 0.0397 | 0.9998 | 4.55 | 0.0528 | 0.0820 | 5.30 | 0.0488 | **0.0039** | 7.84 | 0.0277 | **0.0001** |

**Table S18. Continued**

|  | ***Capsella bursa-pastoris*** | | | ***Raphanus raphanistrum*** | | | ***Raphanus raphanistrum*** | | | ***Raphanus raphanistrum*** | | | ***Raphanus raphanistrum*** | | |
| --- | --- | --- | --- | --- | --- | --- | --- | --- | --- | --- | --- | --- | --- | --- | --- |
|  | ***Erysimum cheiranthoides*** | | | ***Sinapis arvensis*** | | | ***Sinapis alba*** | | | ***Rorippa sylvestris*** | | | ***Rapistrum rugosum*** | | |
| **Species name** | **Contr. (%)** | **SD** | ***P*** | **Contr. (%)** | **SD** | ***P*** | **Contr. (%)** | **SD** | ***P*** | **Contr. (%)** | **SD** | ***P*** | **Contr. (%)** | **SD** | ***P*** |
| *Neocrepidodera transversa* | 6.37 | 0.0658 | **0.0003** | 3.78 | 0.0383 | 0.8070 | 3.77 | 0.0356 | 0.8078 | 3.25 | 0.0320 | 0.9938 | 3.15 | 0.0330 | 0.9972 |
| *Phaedon cochleariae* | 0.37 | 0.0202 | 0.6879 | 0.25 | 0.0135 | 0.7961 | 0.82 | 0.0217 | 0.2114 | 0.36 | 0.0140 | 0.7597 | 0.41 | 0.0156 | 0.7043 |
| *Phyllotreta atra* | 0.66 | 0.0275 | 0.9318 | 1.75 | 0.0299 | 0.2056 | 1.15 | 0.0249 | 0.7108 | 1.42 | 0.0243 | 0.4737 | 0.85 | 0.0203 | 0.8956 |
| *Phyllotreta undulata* | 3.31 | 0.0516 | 0.6532 | 3.71 | 0.0361 | 0.2193 | 3.51 | 0.0343 | 0.4515 | 3.10 | 0.0311 | 0.8259 | 3.18 | 0.0327 | 0.7754 |
| *Meligethes aeneus* | 0.00 | 0.0000 | 1.0000 | 1.93 | 0.0359 | 0.9948 | 1.34 | 0.0314 | 0.9995 | 4.95 | 0.0254 | **0.0085** | 2.11 | 0.0325 | 0.9915 |
| *Murgantia histrionica* | 0.00 | 0.0000 | 1.0000 | 0.37 | 0.0140 | 0.2903 | 0.36 | 0.0134 | 0.3263 | 0.32 | 0.0122 | 0.3450 | 0.33 | 0.0125 | 0.3514 |
| *Athalia rosae* | 0.00 | 0.0000 | 1.0000 | 0.00 | 0.0000 | 1.0000 | 0.85 | 0.0222 | 0.4446 | 2.52 | 0.0296 | **0.0002** | 0.00 | 0.0000 | 1.0000 |
| *Athalia* spp. | 0.00 | 0.0000 | 1.0000 | 0.22 | 0.0117 | 0.7559 | 0.21 | 0.0111 | 0.7968 | 1.47 | 0.0261 | **0.0123** | 0.19 | 0.0103 | 0.8411 |
| Loopers | 0.00 | 0.0000 | 1.0000 | 0.20 | 0.0110 | 0.9240 | 0.19 | 0.0105 | 0.9372 | 4.06 | 0.0288 | **0.0001** | 0.18 | 0.0097 | 0.9549 |
| *Evergestis extimalis* | 0.00 | 0.0000 | 1.0000 | 0.00 | 0.0000 | 1.0000 | 0.00 | 0.0000 | 1.0000 | 0.00 | 0.0000 | 1.0000 | 0.00 | 0.0000 | 1.0000 |
| *Autographa gamma* | 0.00 | 0.0000 | 1.0000 | 0.83 | 0.0214 | 0.5036 | 0.79 | 0.0204 | 0.5549 | 0.86 | 0.0202 | 0.4748 | 1.04 | 0.0223 | 0.3302 |
| *Mamestra brassicae* | 0.66 | 0.0275 | 0.8949 | 0.48 | 0.0186 | 0.9693 | 0.46 | 0.0176 | 0.9729 | 3.32 | 0.0309 | **0.0022** | 0.59 | 0.0184 | 0.9523 |
| *Pieris brassicae* | 0.00 | 0.0000 | 1.0000 | 0.22 | 0.0117 | 0.4628 | 0.60 | 0.0185 | 0.1343 | 0.18 | 0.0100 | 0.6217 | 0.19 | 0.0103 | 0.6236 |
| *Pieris rapae* | 2.31 | 0.0469 | 0.9985 | 3.59 | 0.0378 | 0.7495 | 3.38 | 0.0356 | 0.9088 | 3.09 | 0.0320 | 0.9786 | 3.21 | 0.0326 | 0.9605 |
| *Plutella xylostella* | 1.25 | 0.0358 | **0.0254** | 0.00 | 0.0000 | 1.0000 | 0.00 | 0.0000 | 1.0000 | 0.00 | 0.0000 | 1.0000 | 0.00 | 0.0000 | 1.0000 |
| Cicades | 0.66 | 0.0275 | 0.9809 | 1.31 | 0.0283 | 0.8156 | 1.65 | 0.0285 | 0.5608 | 1.51 | 0.0262 | 0.6808 | 1.40 | 0.0258 | 0.7593 |
| *Aleyrodes proletella* | 0.00 | 0.0000 | 1.0000 | 0.00 | 0.0000 | 1.0000 | 0.00 | 0.0000 | 1.0000 | 0.00 | 0.0000 | 1.0000 | 0.00 | 0.0000 | 1.0000 |
| *Aphis fabae* | 1.18 | 0.0336 | **0.0002** | 0.00 | 0.0000 | 1.0000 | 0.00 | 0.0000 | 1.0000 | 0.00 | 0.0000 | 1.0000 | 0.00 | 0.0000 | 1.0000 |
| *Brevicoryne brassicae* | 0.00 | 0.0000 | 1.0000 | 2.13 | 0.0331 | 0.6819 | 2.01 | 0.0314 | 0.7441 | 1.79 | 0.0280 | 0.8436 | 2.37 | 0.0308 | 0.5259 |
| *Myzus persicae* | 6.86 | 0.0648 | **0.0001** | 0.51 | 0.0198 | 0.9962 | 0.27 | 0.0149 | 0.9986 | 3.08 | 0.0339 | 0.1620 | 0.25 | 0.0134 | 0.9988 |
| *Myzus persicae* subsp. *Nicotiana* | 0.53 | 0.0221 | 0.9585 | 1.32 | 0.0278 | 0.6486 | 2.78 | 0.0340 | **0.0024** | 1.11 | 0.0234 | 0.7927 | 1.39 | 0.0255 | 0.5873 |
| *Lygus* spp. | 2.20 | 0.0443 | 0.9999 | 5.23 | 0.0331 | **0.0014** | 4.42 | 0.0342 | 0.0709 | 1.62 | 0.0303 | 1.0000 | 2.92 | 0.0334 | 0.9984 |
| Leaf mining flies | 4.35 | 0.0633 | **0.0138** | 1.76 | 0.0326 | 0.8398 | 0.68 | 0.0209 | 0.9951 | 3.04 | 0.0334 | 0.2259 | 0.83 | 0.0223 | 0.9912 |
| Weevils | 5.39 | 0.0585 | **0.0001** | 2.88 | 0.0343 | 0.3290 | 2.44 | 0.0314 | 0.6391 | 2.30 | 0.0286 | 0.7354 | 2.39 | 0.0295 | 0.6809 |
| *Dasineura sisymbrii* | 0.00 | 0.0000 | 1.0000 | 0.00 | 0.0000 | 1.0000 | 0.00 | 0.0000 | 1.0000 | 5.83 | 0.0196 | **0.0001** | 0.00 | 0.0000 | 1.0000 |
| Thrips | 5.40 | 0.0555 | **0.0013** | 4.06 | 0.0384 | 0.4607 | 3.29 | 0.0370 | 0.9917 | 2.58 | 0.0331 | 0.9999 | 1.39 | 0.0293 | 1.0000 |

**Table S18. Continued**

|  | ***Raphanus raphanistrum*** | | | ***Raphanus raphanistrum*** | | | ***Raphanus raphanistrum*** | | | ***Erysimum cheiranthoides*** | | | ***Erysimum cheiranthoides*** | | |
| --- | --- | --- | --- | --- | --- | --- | --- | --- | --- | --- | --- | --- | --- | --- | --- |
|  | ***Erysimum cheiranthoides*** | | | ***Capsella bursa-pastoris*** | | | ***Brassica rapa*** | | | ***Sinapis alba*** | | | ***Brassica rapa*** | | |
| **Species name** | **Contr. (%)** | **SD** | ***P*** | **Contr. (%)** | **SD** | ***P*** | **Contr. (%)** | **SD** | ***P*** | **Contr. (%)** | **SD** | ***P*** | **Contr. (%)** | **SD** | ***P*** |
| *Neocrepidodera transversa* | 3.63 | 0.0423 | 0.9125 | 4.18 | 0.0435 | 0.3703 | 3.22 | 0.0335 | 0.9949 | 8.00 | 0.0380 | **0.0001** | 4.20 | 0.0458 | 0.3260 |
| *Phaedon cochleariae* | 0.51 | 0.0194 | 0.5313 | 0.28 | 0.0150 | 0.7475 | 0.58 | 0.0180 | 0.4951 | 1.05 | 0.0279 | 0.0606 | 0.73 | 0.0227 | 0.2830 |
| *Phyllotreta atra* | 0.67 | 0.0202 | 0.9582 | 1.08 | 0.0267 | 0.7294 | 1.06 | 0.0230 | 0.7744 | 0.88 | 0.0266 | 0.8856 | 0.78 | 0.0238 | 0.9240 |
| *Phyllotreta undulata* | 3.98 | 0.0394 | 0.0507 | 4.13 | 0.0408 | **0.0445** | 3.22 | 0.0345 | 0.7500 | 2.43 | 0.0397 | 0.9879 | 5.85 | 0.0422 | **0.0001** |
| *Meligethes aeneus* | 6.66 | 0.0287 | **0.0001** | 6.92 | 0.0300 | **0.0001** | 5.31 | 0.0246 | **0.0025** | 8.99 | 0.0233 | **0.0001** | 0.23 | 0.0124 | 1.0000 |
| *Murgantia histrionica* | 0.39 | 0.0147 | 0.2635 | 0.40 | 0.0152 | 0.2510 | 0.52 | 0.0162 | 0.2041 | 0.00 | 0.0000 | 1.0000 | 0.27 | 0.0145 | 0.3519 |
| *Athalia rosae* | 0.00 | 0.0000 | 1.0000 | 0.00 | 0.0000 | 1.0000 | 0.00 | 0.0000 | 1.0000 | 1.12 | 0.0291 | 0.2510 | 0.00 | 0.0000 | 1.0000 |
| *Athalia* spp. | 0.23 | 0.0124 | 0.7391 | 0.24 | 0.0129 | 0.7011 | 0.19 | 0.0104 | 0.8319 | 0.00 | 0.0000 | 1.0000 | 0.00 | 0.0000 | 1.0000 |
| Loopers | 0.21 | 0.0116 | 0.9056 | 0.22 | 0.0120 | 0.8665 | 0.18 | 0.0098 | 0.9502 | 0.00 | 0.0000 | 1.0000 | 0.00 | 0.0000 | 1.0000 |
| *Evergestis extimalis* | 0.00 | 0.0000 | 1.0000 | 0.00 | 0.0000 | 1.0000 | 0.00 | 0.0000 | 1.0000 | 0.00 | 0.0000 | 1.0000 | 0.00 | 0.0000 | 1.0000 |
| *Autographa gamma* | 0.88 | 0.0227 | 0.4572 | 0.91 | 0.0235 | 0.4365 | 0.89 | 0.0208 | 0.4476 | 0.00 | 0.0000 | 1.0000 | 0.25 | 0.0134 | 0.9210 |
| *Mamestra brassicae* | 0.52 | 0.0201 | 0.9620 | 0.94 | 0.0270 | 0.8099 | 0.59 | 0.0186 | 0.9537 | 0.00 | 0.0000 | 1.0000 | 0.25 | 0.0134 | 0.9878 |
| *Pieris brassicae* | 0.23 | 0.0124 | 0.4580 | 0.24 | 0.0129 | 0.4323 | 0.19 | 0.0104 | 0.6215 | 0.54 | 0.0203 | 0.1606 | 0.00 | 0.0000 | 1.0000 |
| *Pieris rapae* | 3.74 | 0.0405 | 0.5336 | 3.81 | 0.0420 | 0.4304 | 3.29 | 0.0332 | 0.9431 | 3.43 | 0.0440 | 0.8873 | 4.74 | 0.0407 | **0.0018** |
| *Plutella xylostella* | 0.00 | 0.0000 | 1.0000 | 0.89 | 0.0255 | 0.1073 | 0.00 | 0.0000 | 1.0000 | 0.00 | 0.0000 | 1.0000 | 0.00 | 0.0000 | 1.0000 |
| Cicades | 0.97 | 0.0250 | 0.9502 | 1.36 | 0.0300 | 0.7445 | 1.55 | 0.0267 | 0.6467 | 1.35 | 0.0306 | 0.7894 | 1.27 | 0.0287 | 0.8431 |
| *Aleyrodes proletella* | 0.00 | 0.0000 | 1.0000 | 0.00 | 0.0000 | 1.0000 | 0.00 | 0.0000 | 1.0000 | 0.00 | 0.0000 | 1.0000 | 0.00 | 0.0000 | 1.0000 |
| *Aphis fabae* | 0.00 | 0.0000 | 1.0000 | 0.85 | 0.0244 | 0.0504 | 0.00 | 0.0000 | 1.0000 | 0.00 | 0.0000 | 1.0000 | 0.00 | 0.0000 | 1.0000 |
| *Brevicoryne brassicae* | 2.28 | 0.0356 | 0.5829 | 2.37 | 0.0370 | 0.5189 | 4.08 | 0.0323 | **0.0005** | 0.00 | 0.0000 | 1.0000 | 6.83 | 0.0330 | **0.0001** |
| *Myzus persicae* | 4.65 | 0.0429 | **0.0005** | 1.27 | 0.0327 | 0.8963 | 0.25 | 0.0136 | 0.9990 | 5.60 | 0.0512 | **0.0001** | 5.02 | 0.0456 | **0.0001** |
| *Myzus persicae* subsp. *Nicotiana* | 1.23 | 0.0277 | 0.7123 | 1.54 | 0.0309 | 0.4644 | 1.98 | 0.0310 | 0.1529 | 3.25 | 0.0436 | **0.0001** | 1.89 | 0.0386 | 0.2011 |
| *Lygus* spp. | 5.25 | 0.0378 | **0.0019** | 5.85 | 0.0369 | **0.0006** | 2.98 | 0.0340 | 0.9981 | 2.83 | 0.0417 | 0.9998 | 4.68 | 0.0422 | **0.0197** |
| Leaf mining flies | 1.66 | 0.0334 | 0.8707 | 2.83 | 0.0425 | 0.3518 | 1.24 | 0.0269 | 0.9591 | 1.31 | 0.0341 | 0.9533 | 1.91 | 0.0382 | 0.7916 |
| Weevils | 3.28 | 0.0381 | 0.1168 | 3.90 | 0.0413 | **0.0189** | 2.75 | 0.0311 | 0.4261 | 2.60 | 0.0408 | 0.5248 | 3.28 | 0.0404 | 0.1108 |
| *Dasineura sisymbrii* | 0.00 | 0.0000 | 1.0000 | 0.00 | 0.0000 | 1.0000 | 0.00 | 0.0000 | 1.0000 | 0.00 | 0.0000 | 1.0000 | 0.00 | 0.0000 | 1.0000 |
| Thrips | 4.15 | 0.0427 | 0.3468 | 6.37 | 0.0349 | **0.0001** | 4.26 | 0.0319 | 0.2285 | 4.73 | 0.0486 | **0.0195** | 4.06 | 0.0413 | 0.4800 |

**Table S18. Continued**

|  | ***Descurainia sophia*** | | | ***Rorippa sylvestris*** | | | ***Rorippa sylvestris*** | | | ***Rapistrum rugosum*** | | | ***Rapistrum rugosum*** | | |
| --- | --- | --- | --- | --- | --- | --- | --- | --- | --- | --- | --- | --- | --- | --- | --- |
|  | ***Sinapis arvensis*** | | | ***Capsella bursa-pastoris*** | | | ***Brassica rapa*** | | | ***Sinapis alba*** | | | ***Erysimum cheiranthoides*** | | |
| **Species name** | **Contr. (%)** | **SD** | ***P*** | **Contr. (%)** | **SD** | ***P*** | **Contr. (%)** | **SD** | ***P*** | **Contr. (%)** | **SD** | ***P*** | **Contr. (%)** | **SD** | ***P*** |
| *Neocrepidodera transversa* | 4.75 | 0.0528 | **0.0283** | 4.06 | 0.0422 | 0.4783 | 3.28 | 0.0329 | 0.9918 | 3.96 | 0.0371 | 0.6081 | 3.74 | 0.0433 | 0.8463 |
| *Phaedon cochleariae* | 0.00 | 0.0000 | 1.0000 | 0.21 | 0.0112 | 0.8430 | 0.54 | 0.0168 | 0.5080 | 0.84 | 0.0222 | 0.1860 | 0.52 | 0.0198 | 0.5168 |
| *Phyllotreta atra* | 2.09 | 0.0398 | 0.0594 | 1.70 | 0.0315 | 0.2796 | 1.52 | 0.0261 | 0.3835 | 1.02 | 0.0243 | 0.8041 | 0.46 | 0.0173 | 0.9867 |
| *Phyllotreta undulata* | 1.22 | 0.0319 | 1.0000 | 2.77 | 0.0376 | 0.9182 | 4.17 | 0.0341 | **0.0165** | 3.63 | 0.0353 | 0.2995 | 4.14 | 0.0407 | **0.0189** |
| *Meligethes aeneus* | 9.20 | 0.0410 | **0.0001** | 0.49 | 0.0186 | 0.9999 | 0.56 | 0.0173 | 1.0000 | 2.01 | 0.0355 | 0.9947 | 6.18 | 0.0358 | **0.0002** |
| *Murgantia histrionica* | 0.00 | 0.0000 | 1.0000 | 0.00 | 0.0000 | 1.0000 | 0.21 | 0.0113 | 0.4463 | 0.00 | 0.0000 | 1.0000 | 0.00 | 0.0000 | 1.0000 |
| *Athalia rosae* | 0.00 | 0.0000 | 1.0000 | 3.33 | 0.0394 | **0.0002** | 2.64 | 0.0309 | **0.0001** | 0.88 | 0.0229 | 0.4173 | 0.00 | 0.0000 | 1.0000 |
| *Athalia* spp. | 0.98 | 0.0280 | 0.1451 | 1.83 | 0.0343 | **0.0071** | 1.44 | 0.0267 | **0.0169** | 0.00 | 0.0000 | 1.0000 | 0.00 | 0.0000 | 1.0000 |
| Loopers | 0.00 | 0.0000 | 1.0000 | 5.48 | 0.0383 | **0.0001** | 4.32 | 0.0297 | **0.0001** | 0.00 | 0.0000 | 1.0000 | 0.00 | 0.0000 | 1.0000 |
| *Evergestis extimalis* | 0.00 | 0.0000 | 1.0000 | 0.00 | 0.0000 | 1.0000 | 0.00 | 0.0000 | 1.0000 | 0.00 | 0.0000 | 1.0000 | 0.00 | 0.0000 | 1.0000 |
| *Autographa gamma* | 1.50 | 0.0365 | 0.0742 | 0.26 | 0.0139 | 0.8919 | 0.39 | 0.0149 | 0.8749 | 0.45 | 0.0171 | 0.8257 | 0.51 | 0.0194 | 0.7924 |
| *Mamestra brassicae* | 2.87 | 0.0452 | **0.0142** | 4.44 | 0.0420 | **0.0001** | 3.49 | 0.0322 | **0.0006** | 0.22 | 0.0119 | 0.9931 | 0.25 | 0.0134 | 0.9888 |
| *Pieris brassicae* | 0.00 | 0.0000 | 1.0000 | 0.00 | 0.0000 | 1.0000 | 0.00 | 0.0000 | 1.0000 | 0.43 | 0.0162 | 0.2746 | 0.00 | 0.0000 | 1.0000 |
| *Pieris rapae* | 3.65 | 0.0503 | 0.6682 | 4.03 | 0.0420 | 0.1952 | 3.26 | 0.0336 | 0.9498 | 3.71 | 0.0362 | 0.5718 | 4.31 | 0.0403 | **0.0256** |
| *Plutella xylostella* | 0.00 | 0.0000 | 1.0000 | 0.89 | 0.0258 | 0.1072 | 0.00 | 0.0000 | 1.0000 | 0.00 | 0.0000 | 1.0000 | 0.00 | 0.0000 | 1.0000 |
| Cicades | 1.69 | 0.0391 | 0.5248 | 1.63 | 0.0329 | 0.5637 | 1.71 | 0.0281 | 0.5040 | 1.72 | 0.0297 | 0.4866 | 1.03 | 0.0266 | 0.9372 |
| *Aleyrodes proletella* | 0.00 | 0.0000 | 1.0000 | 0.00 | 0.0000 | 1.0000 | 0.00 | 0.0000 | 1.0000 | 0.00 | 0.0000 | 1.0000 | 0.00 | 0.0000 | 1.0000 |
| *Aphis fabae* | 0.00 | 0.0000 | 1.0000 | 0.86 | 0.0247 | 0.0503 | 0.00 | 0.0000 | 1.0000 | 0.00 | 0.0000 | 1.0000 | 0.00 | 0.0000 | 1.0000 |
| *Brevicoryne brassicae* | 1.41 | 0.0343 | 0.9394 | 0.00 | 0.0000 | 1.0000 | 5.27 | 0.0258 | **0.0001** | 1.41 | 0.0286 | 0.9471 | 1.61 | 0.0325 | 0.9016 |
| *Myzus persicae* | 4.17 | 0.0553 | **0.0050** | 4.24 | 0.0464 | **0.0138** | 3.24 | 0.0357 | 0.1095 | 0.00 | 0.0000 | 1.0000 | 4.92 | 0.0447 | **0.0002** |
| *Myzus persicae* subsp. *Nicotiana* | 0.74 | 0.0278 | 0.9451 | 0.65 | 0.0216 | 0.9402 | 1.54 | 0.0296 | 0.4786 | 2.74 | 0.0346 | **0.0033** | 0.72 | 0.0218 | 0.9484 |
| *Lygus* spp. | 5.89 | 0.0517 | **0.0002** | 7.96 | 0.0268 | **0.0001** | 2.73 | 0.0345 | 0.9998 | 3.98 | 0.0367 | 0.4349 | 4.63 | 0.0417 | **0.0252** |
| Leaf mining flies | 6.07 | 0.0559 | **0.0001** | 4.17 | 0.0449 | **0.0258** | 3.19 | 0.0349 | 0.1713 | 0.30 | 0.0163 | 0.9994 | 1.40 | 0.0331 | 0.9365 |
| Weevils | 2.34 | 0.0419 | 0.6922 | 3.66 | 0.0411 | 0.0530 | 2.15 | 0.0297 | 0.8133 | 1.23 | 0.0263 | 0.9939 | 2.60 | 0.0378 | 0.5307 |
| *Dasineura sisymbrii* | 0.00 | 0.0000 | 1.0000 | 7.93 | 0.0280 | **0.0001** | 6.13 | 0.0203 | **0.0001** | 0.00 | 0.0000 | 1.0000 | 0.00 | 0.0000 | 1.0000 |
| Thrips | 4.74 | 0.0538 | **0.0203** | 5.09 | 0.0397 | **0.0072** | 3.68 | 0.0325 | 0.8958 | 3.33 | 0.0386 | 0.9891 | 4.44 | 0.0455 | 0.0905 |

**Table S18. Continued**

|  | ***Rapistrum rugosum*** | | | ***Sisymbrium officinale*** | | | ***Sinapis arvensis*** | | | ***Sinapis arvensis*** | | | ***Sinapis arvensis*** | | |
| --- | --- | --- | --- | --- | --- | --- | --- | --- | --- | --- | --- | --- | --- | --- | --- |
|  | ***Brassica rapa*** | | | ***Brassica nigra*** | | | ***Sinapis alba*** | | | ***Rapistrum rugosum*** | | | ***Erysimum cheiranthoides*** | | |
| **Species name** | **Contr. (%)** | **SD** | ***P*** | **Contr. (%)** | **SD** | ***P*** | **Contr. (%)** | **SD** | ***P*** | **Contr. (%)** | **SD** | ***P*** | **Contr. (%)** | **SD** | ***P*** |
| *Neocrepidodera transversa* | 3.34 | 0.0346 | 0.9875 | 3.20 | 0.0333 | 0.9964 | 3.67 | 0.0414 | 0.8894 | 3.95 | 0.0398 | 0.6180 | 6.05 | 0.0550 | **0.0001** |
| *Phaedon cochleariae* | 0.59 | 0.0184 | 0.4705 | 1.05 | 0.0221 | 0.0646 | 0.75 | 0.0226 | 0.2647 | 0.25 | 0.0135 | 0.7962 | 0.32 | 0.0174 | 0.7683 |
| *Phyllotreta atra* | 0.94 | 0.0223 | 0.8491 | 1.72 | 0.0273 | 0.2288 | 2.07 | 0.0354 | 0.0568 | 1.67 | 0.0301 | 0.2591 | 1.79 | 0.0364 | 0.1802 |
| *Phyllotreta undulata* | 3.34 | 0.0357 | 0.6420 | 3.21 | 0.0334 | 0.7590 | 1.60 | 0.0317 | 1.0000 | 3.85 | 0.0372 | 0.1140 | 2.45 | 0.0421 | 0.9850 |
| *Meligethes aeneus* | 4.91 | 0.0296 | **0.0082** | 3.31 | 0.0299 | 0.7715 | 1.54 | 0.0361 | 0.9988 | 2.52 | 0.0389 | 0.9716 | 8.69 | 0.0381 | **0.0001** |
| *Murgantia histrionica* | 0.21 | 0.0116 | 0.4255 | 0.88 | 0.0206 | 0.0349 | 0.00 | 0.0000 | 1.0000 | 0.00 | 0.0000 | 1.0000 | 0.00 | 0.0000 | 1.0000 |
| *Athalia rosae* | 0.00 | 0.0000 | 1.0000 | 0.85 | 0.0204 | 0.4411 | 1.03 | 0.0269 | 0.2991 | 0.00 | 0.0000 | 1.0000 | 0.00 | 0.0000 | 1.0000 |
| *Athalia* spp. | 0.00 | 0.0000 | 1.0000 | 0.55 | 0.0167 | 0.4578 | 0.00 | 0.0000 | 1.0000 | 0.00 | 0.0000 | 1.0000 | 0.00 | 0.0000 | 1.0000 |
| Loopers | 0.00 | 0.0000 | 1.0000 | 0.70 | 0.0186 | 0.6608 | 0.00 | 0.0000 | 1.0000 | 0.00 | 0.0000 | 1.0000 | 0.00 | 0.0000 | 1.0000 |
| *Evergestis extimalis* | 0.00 | 0.0000 | 1.0000 | 0.55 | 0.0172 | **0.0043** | 0.00 | 0.0000 | 1.0000 | 0.00 | 0.0000 | 1.0000 | 0.00 | 0.0000 | 1.0000 |
| *Autographa gamma* | 0.60 | 0.0185 | 0.7282 | 1.81 | 0.0274 | **0.0153** | 0.00 | 0.0000 | 1.0000 | 0.48 | 0.0180 | 0.8081 | 0.00 | 0.0000 | 1.0000 |
| *Mamestra brassicae* | 0.39 | 0.0150 | 0.9808 | 2.16 | 0.0300 | 0.1635 | 0.00 | 0.0000 | 1.0000 | 0.23 | 0.0125 | 0.9894 | 0.00 | 0.0000 | 1.0000 |
| *Pieris brassicae* | 0.00 | 0.0000 | 1.0000 | 0.74 | 0.0198 | 0.0557 | 0.50 | 0.0189 | 0.1679 | 0.00 | 0.0000 | 1.0000 | 0.00 | 0.0000 | 1.0000 |
| *Pieris rapae* | 3.29 | 0.0346 | 0.9418 | 2.71 | 0.0352 | 0.9971 | 3.69 | 0.0423 | 0.6038 | 3.94 | 0.0384 | 0.2417 | 3.78 | 0.0486 | 0.4681 |
| *Plutella xylostella* | 0.00 | 0.0000 | 1.0000 | 0.00 | 0.0000 | 1.0000 | 0.00 | 0.0000 | 1.0000 | 0.00 | 0.0000 | 1.0000 | 0.00 | 0.0000 | 1.0000 |
| Cicades | 1.61 | 0.0278 | 0.5853 | 2.76 | 0.0322 | **0.0067** | 1.70 | 0.0336 | 0.5093 | 1.38 | 0.0297 | 0.7671 | 0.75 | 0.0284 | 0.9873 |
| *Aleyrodes proletella* | 0.00 | 0.0000 | 1.0000 | 0.00 | 0.0000 | 1.0000 | 0.00 | 0.0000 | 1.0000 | 0.00 | 0.0000 | 1.0000 | 0.00 | 0.0000 | 1.0000 |
| *Aphis fabae* | 0.00 | 0.0000 | 1.0000 | 0.00 | 0.0000 | 1.0000 | 0.00 | 0.0000 | 1.0000 | 0.00 | 0.0000 | 1.0000 | 0.00 | 0.0000 | 1.0000 |
| *Brevicoryne brassicae* | 4.62 | 0.0315 | **0.0001** | 3.11 | 0.0322 | 0.0922 | 0.00 | 0.0000 | 1.0000 | 1.50 | 0.0302 | 0.9306 | 0.00 | 0.0000 | 1.0000 |
| *Myzus persicae* | 0.00 | 0.0000 | 1.0000 | 0.27 | 0.0148 | 0.9985 | 0.27 | 0.0149 | 0.9991 | 0.25 | 0.0134 | 0.9997 | 6.01 | 0.0551 | **0.0001** |
| *Myzus persicae* subsp. *Nicotiana* | 1.80 | 0.0311 | 0.2659 | 1.36 | 0.0262 | 0.6135 | 3.10 | 0.0406 | **0.0002** | 0.90 | 0.0238 | 0.8986 | 0.35 | 0.0192 | 0.9933 |
| *Lygus* spp. | 3.26 | 0.0347 | 0.9900 | 3.76 | 0.0353 | 0.7503 | 2.12 | 0.0353 | 0.9999 | 4.43 | 0.0381 | 0.0640 | 1.93 | 0.0377 | 1.0000 |
| Leaf mining flies | 0.98 | 0.0261 | 0.9833 | 1.40 | 0.0309 | 0.9379 | 1.50 | 0.0342 | 0.9135 | 1.55 | 0.0326 | 0.9072 | 2.70 | 0.0471 | 0.3942 |
| Weevils | 2.24 | 0.0308 | 0.7650 | 0.91 | 0.0217 | 0.9990 | 1.85 | 0.0333 | 0.9166 | 2.00 | 0.0324 | 0.8721 | 3.39 | 0.0461 | 0.0699 |
| *Dasineura sisymbrii* | 0.00 | 0.0000 | 1.0000 | 0.00 | 0.0000 | 1.0000 | 0.00 | 0.0000 | 1.0000 | 0.00 | 0.0000 | 1.0000 | 0.00 | 0.0000 | 1.0000 |
| Thrips | 4.93 | 0.0321 | **0.0069** | 4.00 | 0.0314 | 0.5516 | 4.38 | 0.0443 | 0.1210 | 4.42 | 0.0401 | 0.1023 | 5.00 | 0.0513 | **0.0041** |

**Table S18. Continued**

|  | ***Sinapis arvensis*** | | | ***Sinapis alba*** | | | ***Rorippa sylvestris*** | | | ***Rorippa sylvestris*** | | | ***Rorippa sylvestris*** | | |
| --- | --- | --- | --- | --- | --- | --- | --- | --- | --- | --- | --- | --- | --- | --- | --- |
|  | ***Brassica rapa*** | | | ***Brassica rapa*** | | | ***Sinapis arvensis*** | | | ***Sinapis alba*** | | | ***Rapistrum rugosum*** | | |
| **Species name** | **Contr. (%)** | **SD** | ***P*** | **Contr. (%)** | **SD** | ***P*** | **Contr. (%)** | **SD** | ***P*** | **Contr. (%)** | **SD** | ***P*** | **Contr. (%)** | **SD** | ***P*** |
| *Neocrepidodera transversa* | 3.93 | 0.0399 | 0.6458 | 3.74 | 0.0367 | 0.8395 | 3.37 | 0.0365 | 0.9852 | 2.23 | 0.0308 | 1.0000 | 3.38 | 0.0332 | 0.9828 |
| *Phaedon cochleariae* | 0.46 | 0.0174 | 0.5847 | 1.01 | 0.0240 | 0.0821 | 0.19 | 0.0103 | 0.9127 | 0.77 | 0.0206 | 0.2436 | 0.37 | 0.0142 | 0.7568 |
| *Phyllotreta atra* | 1.89 | 0.0323 | 0.1290 | 1.26 | 0.0272 | 0.6199 | 2.15 | 0.0320 | **0.0394** | 1.64 | 0.0281 | 0.2835 | 1.35 | 0.0242 | 0.5452 |
| *Phyllotreta undulata* | 5.87 | 0.0367 | **0.0001** | 5.41 | 0.0355 | **0.0001** | 2.27 | 0.0324 | 0.9936 | 2.24 | 0.0310 | 0.9939 | 3.21 | 0.0320 | 0.7568 |
| *Meligethes aeneus* | 6.57 | 0.0309 | **0.0001** | 6.89 | 0.0215 | **0.0001** | 6.08 | 0.0317 | **0.0002** | 6.35 | 0.0244 | **0.0001** | 4.60 | 0.0294 | **0.0240** |
| *Murgantia histrionica* | 0.25 | 0.0135 | 0.3521 | 0.23 | 0.0127 | 0.3953 | 0.00 | 0.0000 | 1.0000 | 0.00 | 0.0000 | 1.0000 | 0.00 | 0.0000 | 1.0000 |
| *Athalia rosae* | 0.00 | 0.0000 | 1.0000 | 0.90 | 0.0233 | 0.4001 | 3.00 | 0.0352 | **0.0001** | 3.00 | 0.0341 | **0.0001** | 2.60 | 0.0305 | **0.0001** |
| *Athalia* spp. | 0.00 | 0.0000 | 1.0000 | 0.00 | 0.0000 | 1.0000 | 1.64 | 0.0305 | **0.0040** | 1.55 | 0.0289 | **0.0079** | 1.42 | 0.0263 | **0.0193** |
| Loopers | 0.00 | 0.0000 | 1.0000 | 0.00 | 0.0000 | 1.0000 | 4.92 | 0.0340 | **0.0001** | 4.66 | 0.0322 | **0.0001** | 4.26 | 0.0292 | **0.0001** |
| *Evergestis extimalis* | 0.00 | 0.0000 | 1.0000 | 0.00 | 0.0000 | 1.0000 | 0.00 | 0.0000 | 1.0000 | 0.00 | 0.0000 | 1.0000 | 0.00 | 0.0000 | 1.0000 |
| *Autographa gamma* | 0.23 | 0.0125 | 0.9257 | 0.22 | 0.0119 | 0.9396 | 0.23 | 0.0125 | 0.9251 | 0.22 | 0.0119 | 0.9371 | 0.58 | 0.0180 | 0.7438 |
| *Mamestra brassicae* | 0.23 | 0.0125 | 0.9874 | 0.22 | 0.0119 | 0.9939 | 4.01 | 0.0369 | **0.0001** | 3.79 | 0.0349 | **0.0002** | 3.45 | 0.0317 | **0.0006** |
| *Pieris brassicae* | 0.00 | 0.0000 | 1.0000 | 0.44 | 0.0165 | 0.2736 | 0.00 | 0.0000 | 1.0000 | 0.42 | 0.0158 | 0.2828 | 0.00 | 0.0000 | 1.0000 |
| *Pieris rapae* | 4.18 | 0.0390 | 0.0574 | 3.95 | 0.0369 | 0.2361 | 3.68 | 0.0381 | 0.6381 | 3.47 | 0.0359 | 0.8566 | 3.21 | 0.0330 | 0.9634 |
| *Plutella xylostella* | 0.00 | 0.0000 | 1.0000 | 0.00 | 0.0000 | 1.0000 | 0.00 | 0.0000 | 1.0000 | 0.00 | 0.0000 | 1.0000 | 0.00 | 0.0000 | 1.0000 |
| Cicades | 1.58 | 0.0311 | 0.6169 | 1.88 | 0.0307 | 0.3528 | 1.54 | 0.0305 | 0.6510 | 1.83 | 0.0300 | 0.4024 | 1.57 | 0.0272 | 0.6253 |
| *Aleyrodes proletella* | 0.00 | 0.0000 | 1.0000 | 0.00 | 0.0000 | 1.0000 | 0.00 | 0.0000 | 1.0000 | 0.00 | 0.0000 | 1.0000 | 0.00 | 0.0000 | 1.0000 |
| *Aphis fabae* | 0.00 | 0.0000 | 1.0000 | 0.00 | 0.0000 | 1.0000 | 0.00 | 0.0000 | 1.0000 | 0.00 | 0.0000 | 1.0000 | 0.00 | 0.0000 | 1.0000 |
| *Brevicoryne brassicae* | 6.34 | 0.0303 | **0.0001** | 5.98 | 0.0288 | **0.0001** | 0.00 | 0.0000 | 1.0000 | 0.00 | 0.0000 | 1.0000 | 1.25 | 0.0254 | 0.9669 |
| *Myzus persicae* | 0.25 | 0.0136 | 0.9989 | 0.00 | 0.0000 | 1.0000 | 3.76 | 0.0414 | **0.0228** | 3.53 | 0.0391 | **0.0489** | 3.20 | 0.0352 | 0.1229 |
| *Myzus persicae* subsp. *Nicotiana* | 1.89 | 0.0362 | 0.2118 | 3.08 | 0.0373 | **0.0002** | 0.49 | 0.0188 | 0.9864 | 2.54 | 0.0336 | **0.0113** | 0.75 | 0.0199 | 0.9469 |
| *Lygus* spp. | 4.46 | 0.0385 | 0.0502 | 4.02 | 0.0371 | 0.3914 | 7.05 | 0.0237 | **0.0001** | 5.52 | 0.0331 | **0.0005** | 2.66 | 0.0336 | 0.9999 |
| Leaf mining flies | 1.97 | 0.0363 | 0.7682 | 0.87 | 0.0265 | 0.9896 | 3.70 | 0.0403 | **0.0425** | 3.46 | 0.0387 | 0.0851 | 3.14 | 0.0346 | 0.1923 |
| Weevils | 2.79 | 0.0362 | 0.3917 | 2.24 | 0.0327 | 0.7641 | 1.88 | 0.0308 | 0.9125 | 1.12 | 0.0243 | 0.9968 | 1.36 | 0.0253 | 0.9887 |
| *Dasineura sisymbrii* | 0.00 | 0.0000 | 1.0000 | 0.00 | 0.0000 | 1.0000 | 7.05 | 0.0238 | **0.0001** | 6.65 | 0.0225 | **0.0001** | 6.04 | 0.0199 | **0.0001** |
| Thrips | 3.59 | 0.0391 | 0.9389 | 3.87 | 0.0373 | 0.7270 | 3.88 | 0.0384 | 0.7147 | 3.43 | 0.0370 | 0.9796 | 2.43 | 0.0342 | 1.0000 |

**Table S18. Continued**

|  | ***Rorippa sylvestris*** | | | ***Sisymbrium officinale*** | | | ***Sisymbrium officinale*** | | | ***Sisymbrium officinale*** | | | ***Sisymbrium officinale*** | | |
| --- | --- | --- | --- | --- | --- | --- | --- | --- | --- | --- | --- | --- | --- | --- | --- |
|  | ***Erysimum cheiranthoides*** | | | ***Raphanus raphanistrum*** | | | ***Descurainia sophia*** | | | ***Capsella bursa-pastoris*** | | | ***Camelina sativa*** | | |
| **Species name** | **Contr. (%)** | **SD** | ***P*** | **Contr. (%)** | **SD** | ***P*** | **Contr. (%)** | **SD** | ***P*** | **Contr. (%)** | **SD** | ***P*** | **Contr. (%)** | **SD** | ***P*** |
| *Neocrepidodera transversa* | 5.84 | 0.0417 | **0.0002** | 3.07 | 0.0319 | 0.9983 | 4.20 | 0.0423 | 0.3207 | 4.14 | 0.0429 | 0.4004 | 3.93 | 0.0410 | 0.6447 |
| *Phaedon cochleariae* | 0.45 | 0.0173 | 0.6113 | 0.35 | 0.0137 | 0.7679 | 0.20 | 0.0106 | 0.9089 | 0.20 | 0.0106 | 0.8717 | 0.19 | 0.0102 | 0.9181 |
| *Phyllotreta atra* | 1.34 | 0.0272 | 0.5555 | 1.78 | 0.0269 | 0.1902 | 2.13 | 0.0347 | 0.0526 | 2.22 | 0.0356 | 0.0553 | 2.34 | 0.0352 | **0.0160** |
| *Phyllotreta undulata* | 2.80 | 0.0369 | 0.9397 | 3.07 | 0.0316 | 0.8380 | 3.97 | 0.0411 | 0.0602 | 4.00 | 0.0413 | 0.0819 | 3.79 | 0.0391 | 0.1579 |
| *Meligethes aeneus* | 0.48 | 0.0179 | 1.0000 | 5.18 | 0.0223 | **0.0032** | 0.00 | 0.0000 | 1.0000 | 0.00 | 0.0000 | 1.0000 | 0.28 | 0.0146 | 1.0000 |
| *Murgantia histrionica* | 0.00 | 0.0000 | 1.0000 | 0.96 | 0.0206 | **0.0185** | 0.93 | 0.0239 | **0.0351** | 0.92 | 0.0238 | 0.0721 | 0.89 | 0.0227 | **0.0475** |
| *Athalia rosae* | 3.21 | 0.0379 | **0.0001** | 0.52 | 0.0156 | 0.6990 | 0.66 | 0.0199 | 0.5980 | 0.66 | 0.0198 | 0.5875 | 0.63 | 0.0190 | 0.6239 |
| *Athalia* spp. | 1.76 | 0.0329 | **0.0015** | 0.68 | 0.0181 | 0.3380 | 1.34 | 0.0283 | **0.0326** | 0.68 | 0.0208 | 0.3444 | 0.65 | 0.0199 | 0.3695 |
| Loopers | 5.27 | 0.0367 | **0.0001** | 0.67 | 0.0176 | 0.6777 | 0.67 | 0.0202 | 0.6652 | 0.67 | 0.0202 | 0.6419 | 0.64 | 0.0193 | 0.6917 |
| *Evergestis extimalis* | 0.00 | 0.0000 | 1.0000 | 0.21 | 0.0112 | 0.3288 | 0.28 | 0.0151 | 0.0739 | 0.28 | 0.0150 | 0.1427 | 0.26 | 0.0143 | 0.1172 |
| *Autographa gamma* | 0.25 | 0.0134 | 0.9173 | 1.86 | 0.0270 | **0.0102** | 2.58 | 0.0372 | **0.0002** | 1.95 | 0.0330 | **0.0215** | 1.87 | 0.0315 | **0.0146** |
| *Mamestra brassicae* | 4.30 | 0.0399 | **0.0001** | 2.09 | 0.0288 | 0.2032 | 3.44 | 0.0405 | **0.0005** | 2.75 | 0.0382 | **0.0498** | 2.45 | 0.0354 | 0.0778 |
| *Pieris brassicae* | 0.00 | 0.0000 | 1.0000 | 0.69 | 0.0183 | 0.0707 | 0.70 | 0.0211 | 0.0808 | 0.70 | 0.0210 | 0.1210 | 0.67 | 0.0201 | 0.0986 |
| *Pieris rapae* | 3.90 | 0.0406 | 0.2841 | 3.28 | 0.0322 | 0.9427 | 6.93 | 0.0339 | **0.0001** | 7.00 | 0.0327 | **0.0001** | 6.34 | 0.0333 | **0.0001** |
| *Plutella xylostella* | 0.00 | 0.0000 | 1.0000 | 0.00 | 0.0000 | 1.0000 | 0.00 | 0.0000 | 1.0000 | 0.89 | 0.0255 | 0.1114 | 1.59 | 0.0341 | **0.0014** |
| Cicades | 1.26 | 0.0286 | 0.8517 | 2.70 | 0.0308 | **0.0108** | 3.60 | 0.0417 | **0.0001** | 3.52 | 0.0415 | **0.0004** | 3.36 | 0.0392 | **0.0001** |
| *Aleyrodes proletella* | 0.00 | 0.0000 | 1.0000 | 0.00 | 0.0000 | 1.0000 | 0.00 | 0.0000 | 1.0000 | 0.00 | 0.0000 | 1.0000 | 0.00 | 0.0000 | 1.0000 |
| *Aphis fabae* | 0.00 | 0.0000 | 1.0000 | 0.00 | 0.0000 | 1.0000 | 0.00 | 0.0000 | 1.0000 | 0.85 | 0.0244 | 0.0503 | 0.00 | 0.0000 | 1.0000 |
| *Brevicoryne brassicae* | 0.00 | 0.0000 | 1.0000 | 2.75 | 0.0306 | 0.2660 | 3.35 | 0.0396 | **0.0390** | 3.05 | 0.0382 | 0.1582 | 2.91 | 0.0364 | 0.1867 |
| *Myzus persicae* | 4.00 | 0.0412 | **0.0092** | 0.47 | 0.0182 | 0.9965 | 3.23 | 0.0429 | 0.1206 | 1.30 | 0.0333 | 0.8882 | 2.11 | 0.0382 | 0.6343 |
| *Myzus persicae* subsp. *Nicotiana* | 0.27 | 0.0145 | 0.9957 | 1.08 | 0.0227 | 0.8080 | 0.51 | 0.0194 | 0.9791 | 0.59 | 0.0197 | 0.9445 | 0.71 | 0.0214 | 0.9488 |
| *Lygus* spp. | 6.80 | 0.0345 | **0.0001** | 1.83 | 0.0309 | 1.0000 | 3.74 | 0.0453 | 0.7718 | 7.27 | 0.0303 | **0.0001** | 6.83 | 0.0297 | **0.0001** |
| Leaf mining flies | 4.00 | 0.0440 | **0.0167** | 0.93 | 0.0222 | 0.9872 | 4.86 | 0.0434 | **0.0010** | 2.71 | 0.0420 | 0.4005 | 0.78 | 0.0240 | 0.9915 |
| Weevils | 2.46 | 0.0362 | 0.6292 | 2.13 | 0.0279 | 0.8245 | 0.85 | 0.0256 | 0.9993 | 3.53 | 0.0403 | 0.0785 | 3.89 | 0.0388 | **0.0095** |
| *Dasineura sisymbrii* | 7.60 | 0.0265 | **0.0001** | 0.00 | 0.0000 | 1.0000 | 0.00 | 0.0000 | 1.0000 | 0.00 | 0.0000 | 1.0000 | 0.00 | 0.0000 | 1.0000 |
| Thrips | 4.07 | 0.0420 | 0.4575 | 4.00 | 0.0303 | 0.5471 | 2.69 | 0.0387 | 0.9997 | 2.31 | 0.0365 | 0.9999 | 3.92 | 0.0397 | 0.6552 |

**Table S18. Continued**

|  | ***Sisymbrium officinale*** | | | ***Sisymbrium officinale*** | | | ***Sisymbrium officinale*** | | | ***Sisymbrium officinale*** | | | ***Sisymbrium officinale*** | | |
| --- | --- | --- | --- | --- | --- | --- | --- | --- | --- | --- | --- | --- | --- | --- | --- |
|  | ***Brassica rapa*** | | | ***Rorippa sylvestris*** | | | ***Sinapis alba*** | | | ***Sinapis arvensis*** | | | ***Rapistrum rugosum*** | | |
| **Species name** | **Contr. (%)** | **SD** | ***P*** | **Contr. (%)** | **SD** | ***P*** | **Contr. (%)** | **SD** | ***P*** | **Contr. (%)** | **SD** | ***P*** | **Contr. (%)** | **SD** | ***P*** |
| *Neocrepidodera transversa* | 3.22 | 0.0333 | 0.9954 | 3.12 | 0.0315 | 0.9983 | 3.55 | 0.0350 | 0.9503 | 3.71 | 0.0378 | 0.8650 | 3.17 | 0.0329 | 0.9953 |
| *Phaedon cochleariae* | 0.53 | 0.0165 | 0.5203 | 0.31 | 0.0120 | 0.7686 | 0.76 | 0.0203 | 0.2613 | 0.18 | 0.0098 | 0.9325 | 0.36 | 0.0138 | 0.7633 |
| *Phyllotreta atra* | 1.89 | 0.0285 | 0.1233 | 2.06 | 0.0281 | 0.0667 | 2.04 | 0.0309 | 0.0661 | 2.48 | 0.0338 | **0.0045** | 1.74 | 0.0273 | 0.2161 |
| *Phyllotreta undulata* | 3.31 | 0.0341 | 0.6732 | 3.03 | 0.0313 | 0.8610 | 3.38 | 0.0347 | 0.5971 | 3.56 | 0.0366 | 0.3836 | 3.18 | 0.0326 | 0.7780 |
| *Meligethes aeneus* | 0.18 | 0.0100 | 1.0000 | 0.38 | 0.0143 | 1.0000 | 6.69 | 0.0172 | **0.0001** | 6.35 | 0.0277 | **0.0001** | 4.76 | 0.0276 | **0.0126** |
| *Murgantia histrionica* | 0.91 | 0.0212 | **0.0315** | 0.72 | 0.0186 | 0.1039 | 0.80 | 0.0206 | 0.0645 | 0.84 | 0.0216 | **0.0434** | 0.74 | 0.0190 | 0.1061 |
| *Athalia rosae* | 0.54 | 0.0162 | 0.6853 | 2.61 | 0.0298 | **0.0002** | 1.28 | 0.0256 | 0.1543 | 0.60 | 0.0181 | 0.6577 | 0.53 | 0.0160 | 0.6917 |
| *Athalia* spp. | 0.55 | 0.0168 | 0.4435 | 1.66 | 0.0270 | **0.0031** | 0.59 | 0.0180 | 0.4357 | 0.62 | 0.0189 | 0.4004 | 0.55 | 0.0166 | 0.4645 |
| Loopers | 0.55 | 0.0165 | 0.7719 | 3.94 | 0.0295 | **0.0001** | 0.58 | 0.0176 | 0.7532 | 0.61 | 0.0184 | 0.7241 | 0.54 | 0.0163 | 0.7799 |
| *Evergestis extimalis* | 0.22 | 0.0118 | 0.3165 | 0.21 | 0.0113 | 0.3226 | 0.23 | 0.0127 | 0.2071 | 0.25 | 0.0135 | 0.0989 | 0.21 | 0.0116 | 0.3224 |
| *Autographa gamma* | 1.66 | 0.0269 | **0.0344** | 1.60 | 0.0260 | **0.0442** | 1.68 | 0.0283 | **0.0318** | 1.77 | 0.0298 | **0.0174** | 1.75 | 0.0273 | **0.0222** |
| *Mamestra brassicae* | 2.11 | 0.0296 | 0.1897 | 3.18 | 0.0316 | **0.0035** | 2.19 | 0.0316 | 0.1498 | 2.31 | 0.0333 | 0.1092 | 2.08 | 0.0292 | 0.2001 |
| *Pieris brassicae* | 0.56 | 0.0170 | 0.1569 | 0.54 | 0.0165 | 0.1581 | 0.95 | 0.0224 | **0.0121** | 0.63 | 0.0191 | 0.1100 | 0.56 | 0.0168 | 0.1558 |
| *Pieris rapae* | 2.74 | 0.0347 | 0.9961 | 3.17 | 0.0329 | 0.9696 | 4.53 | 0.0360 | **0.0078** | 4.75 | 0.0377 | **0.0025** | 3.00 | 0.0343 | 0.9853 |
| *Plutella xylostella* | 0.00 | 0.0000 | 1.0000 | 0.00 | 0.0000 | 1.0000 | 0.00 | 0.0000 | 1.0000 | 0.00 | 0.0000 | 1.0000 | 0.00 | 0.0000 | 1.0000 |
| Cicades | 2.86 | 0.0322 | **0.0037** | 2.75 | 0.0312 | **0.0095** | 3.09 | 0.0349 | **0.0004** | 3.17 | 0.0370 | **0.0002** | 2.79 | 0.0318 | **0.0051** |
| *Aleyrodes proletella* | 0.00 | 0.0000 | 1.0000 | 0.00 | 0.0000 | 1.0000 | 0.00 | 0.0000 | 1.0000 | 0.00 | 0.0000 | 1.0000 | 0.00 | 0.0000 | 1.0000 |
| *Aphis fabae* | 0.00 | 0.0000 | 1.0000 | 0.00 | 0.0000 | 1.0000 | 0.00 | 0.0000 | 1.0000 | 0.00 | 0.0000 | 1.0000 | 0.00 | 0.0000 | 1.0000 |
| *Brevicoryne brassicae* | 3.73 | 0.0336 | **0.0050** | 2.32 | 0.0292 | 0.5475 | 2.61 | 0.0326 | 0.3606 | 2.75 | 0.0343 | 0.2642 | 2.70 | 0.0311 | 0.2968 |
| *Myzus persicae* | 0.27 | 0.0147 | 0.9992 | 3.07 | 0.0337 | 0.1670 | 0.30 | 0.0163 | 0.9987 | 0.54 | 0.0209 | 0.9948 | 0.27 | 0.0145 | 0.9991 |
| *Myzus persicae* subsp. *Nicotiana* | 1.51 | 0.0290 | 0.4991 | 0.37 | 0.0143 | 0.9932 | 2.51 | 0.0330 | **0.0124** | 0.44 | 0.0172 | 0.9910 | 0.72 | 0.0190 | 0.9489 |
| *Lygus* spp. | 2.79 | 0.0340 | 0.9992 | 0.48 | 0.0181 | 1.0000 | 5.16 | 0.0332 | **0.0018** | 6.47 | 0.0272 | **0.0001** | 2.72 | 0.0333 | 0.9997 |
| Leaf mining flies | 1.07 | 0.0257 | 0.9766 | 3.03 | 0.0334 | 0.2389 | 0.46 | 0.0174 | 0.9987 | 1.60 | 0.0315 | 0.8944 | 0.64 | 0.0200 | 0.9963 |
| Weevils | 1.91 | 0.0288 | 0.9075 | 0.89 | 0.0211 | 0.9987 | 0.64 | 0.0199 | 0.9998 | 1.50 | 0.0288 | 0.9759 | 0.97 | 0.0228 | 0.9983 |
| *Dasineura sisymbrii* | 0.00 | 0.0000 | 1.0000 | 5.82 | 0.0195 | **0.0001** | 0.00 | 0.0000 | 1.0000 | 0.00 | 0.0000 | 1.0000 | 0.00 | 0.0000 | 1.0000 |
| Thrips | 2.41 | 0.0305 | 1.0000 | 3.49 | 0.0310 | 0.9704 | 3.66 | 0.0354 | 0.9137 | 3.41 | 0.0373 | 0.9816 | 4.61 | 0.0304 | **0.0370** |

**Table S18. Continued**

|  | ***Sisymbrium officinale*** | | |
| --- | --- | --- | --- |
|  | ***Erysimum cheiranthoides*** | | |
| **Species name** | **Contr. (%)** | **SD** | ***P*** |
| *Neocrepidodera transversa* | 3.92 | 0.0428 | 0.6671 |
| *Phaedon cochleariae* | 0.43 | 0.0168 | 0.6466 |
| *Phyllotreta atra* | 1.91 | 0.0326 | 0.1173 |
| *Phyllotreta undulata* | 3.86 | 0.0398 | 0.1004 |
| *Meligethes aeneus* | 0.00 | 0.0000 | 1.0000 |
| *Murgantia histrionica* | 0.89 | 0.0230 | **0.0326** |
| *Athalia rosae* | 0.64 | 0.0192 | 0.6244 |
| *Athalia* spp. | 0.66 | 0.0201 | 0.3490 |
| Loopers | 0.65 | 0.0195 | 0.6882 |
| *Evergestis extimalis* | 0.27 | 0.0145 | 0.0796 |
| *Autographa gamma* | 1.89 | 0.0318 | **0.0084** |
| *Mamestra brassicae* | 2.48 | 0.0358 | 0.0696 |
| *Pieris brassicae* | 0.67 | 0.0203 | 0.0808 |
| *Pieris rapae* | 6.09 | 0.0362 | **0.0001** |
| *Plutella xylostella* | 0.00 | 0.0000 | 1.0000 |
| Cicades | 3.31 | 0.0395 | **0.0001** |
| *Aleyrodes proletella* | 0.00 | 0.0000 | 1.0000 |
| *Aphis fabae* | 0.00 | 0.0000 | 1.0000 |
| *Brevicoryne brassicae* | 2.94 | 0.0368 | 0.1657 |
| *Myzus persicae* | 4.63 | 0.0425 | **0.0004** |
| *Myzus persicae* subsp. *Nicotiana* | 0.21 | 0.0116 | 0.9986 |
| *Lygus* spp. | 6.29 | 0.0353 | **0.0002** |
| Leaf mining flies | 1.47 | 0.0319 | 0.9193 |
| Weevils | 2.14 | 0.0350 | 0.8185 |
| *Dasineura sisymbrii* | 0.00 | 0.0000 | 1.0000 |
| Thrips | 3.83 | 0.0393 | 0.7751 |

**Table S19.** Results of linear mixed models (LMM) testing whether plant species differed in the measured phenotypic traits. The plot in which the plant individuals were planted was included in our models as random intercept, and we allowed variance to be different for the different plant species to account for heterogeneity of variance. Significant *P* values (*P* < 0.05) are presented in bold.

| **Parameter** | **df** | **ꭓ^2^** | ***P*** | **Plant species** | **Estimated mean** |  | **SE** |
| --- | --- | --- | --- | --- | --- | --- | --- |
| Plant height (cm) | 11 | 710.04 | **< 0.0001** | *Capsella bursa-pastoris* | 18.36 | ± | 3.13 |
|  |  |  |  | *Camelina sativa* | 48.21 | ± | 2.28 |
|  |  |  |  | *Erysimum cheiranthoides* | 65.88 | ± | 2.54 |
|  |  |  |  | *Rorippa sylvestris* | 52.76 | ± | 1.74 |
|  |  |  |  | *Descurainia sophia* | 25.63 | ± | 3.93 |
|  |  |  |  | *Brassica nigra* | 97.52 | ± | 4.82 |
|  |  |  |  | *Sinapis arvensis* | 38.62 | ± | 1.47 |
|  |  |  |  | *Rapistrum rugosum* | 86.08 | ± | 2.81 |
|  |  |  |  | *Raphanus raphanistrum* | 47.51 | ± | 2.03 |
|  |  |  |  | *Brassica rapa* | 17.06 | ± | 3.08 |
|  |  |  |  | *Sinapis alba* | 57.94 | ± | 1.73 |
|  |  |  |  | *Sisymbrium officinale* | 86.24 | ± | 4.40 |
| Plant diameter (cm) | 11 | 622.51 | **< 0.0001** | *Capsella bursa-pastoris* | 30.79 | ± | 1.36 |
|  |  |  |  | *Camelina sativa* | 29.08 | ± | 1.93 |
|  |  |  |  | *Erysimum cheiranthoides* | 43.29 | ± | 2.78 |
|  |  |  |  | *Rorippa sylvestris* | 40.39 | ± | 1.35 |
|  |  |  |  | *Descurainia sophia* | 29.31 | ± | 1.38 |
|  |  |  |  | *Brassica nigra* | 73.60 | ± | 4.38 |
|  |  |  |  | *Sinapis arvensis* | 29.13 | ± | 1.54 |
|  |  |  |  | *Rapistrum rugosum* | 81.65 | ± | 5.14 |
|  |  |  |  | *Raphanus raphanistrum* | 87.13 | ± | 4.39 |
|  |  |  |  | *Brassica rapa* | 60.88 | ± | 2.24 |
|  |  |  |  | *Sinapis alba* | 34.02 | ± | 2.07 |
|  |  |  |  | *Sisymbrium officinale* | 60.98 | ± | 2.07 |
| Length of largest leaf (cm) | 11 | 1839.50 | **< 0.0001** | *Capsella bursa-pastoris* | 14.35 | ± | 0.61 |
|  |  |  |  | *Camelina sativa* | 10.13 | ± | 0.19 |
|  |  |  |  | *Erysimum cheiranthoides* | 8.33 | ± | 0.14 |
|  |  |  |  | *Rorippa sylvestris* | 19.70 | ± | 0.56 |
|  |  |  |  | *Descurainia sophia* | 13.32 | ± | 0.46 |
|  |  |  |  | *Brassica nigra* | 21.16 | ± | 0.83 |
|  |  |  |  | *Sinapis arvensis* | 12.77 | ± | 0.38 |
|  |  |  |  | *Rapistrum rugosum* | 21.12 | ± | 0.65 |
|  |  |  |  | *Raphanus raphanistrum* | 20.51 | ± | 1.00 |
|  |  |  |  | *Brassica rapa* | 30.24 | ± | 1.18 |
|  |  |  |  | *Sinapis alba* | 11.90 | ± | 0.31 |
|  |  |  |  | *Sisymbrium officinale* | 28.78 | ± | 0.92 |

**Table S19. Continued.**

| **Parameter** | **df** | **ꭓ^2^** | ***P*** | **Plant species** | **Estimated mean** |  | **SE** |
| --- | --- | --- | --- | --- | --- | --- | --- |
| Number of true leaves | 11 | 1090.20 | **< 0.0001** | *Capsella bursa-pastoris* | 125.48 | ± | 14.68 |
|  |  |  |  | *Camelina sativa* | 257.47 | ± | 19.52 |
|  |  |  |  | *Erysimum cheiranthoides* | 452.71 | ± | 36.66 |
|  |  |  |  | *Rorippa sylvestris* | 163.62 | ± | 10.06 |
|  |  |  |  | *Descurainia sophia* | 164.54 | ± | 14.34 |
|  |  |  |  | *Brassica nigra* | 168.70 | ± | 13.93 |
|  |  |  |  | *Sinapis arvensis* | 20.31 | ± | 1.18 |
|  |  |  |  | *Rapistrum rugosum* | 199.86 | ± | 25.18 |
|  |  |  |  | *Raphanus raphanistrum* | 151.47 | ± | 11.59 |
|  |  |  |  | *Brassica rapa* | 46.02 | ± | 3.78 |
|  |  |  |  | *Sinapis alba* | 65.30 | ± | 6.13 |
|  |  |  |  | *Sisymbrium officinale* | 628.54 | ± | 50.17 |
| Number of flower axes | 11 | 425.23 | **< 0.0001** | *Capsella bursa-pastoris* | 7.49 | ± | 1.95 |
|  |  |  |  | *Camelina sativa* | 10.91 | ± | 1.35 |
|  |  |  |  | *Erysimum cheiranthoides* | 22.22 | ± | 4.46 |
|  |  |  |  | *Rorippa sylvestris* | 9.27 | ± | 0.95 |
|  |  |  |  | *Descurainia sophia* | 0.73 | ± | 0.23 |
|  |  |  |  | *Brassica nigra* | 63.36 | ± | 12.02 |
|  |  |  |  | *Sinapis arvensis* | 5.04 | ± | 0.40 |
|  |  |  |  | *Rapistrum rugosum* | 69.08 | ± | 9.41 |
|  |  |  |  | *Raphanus raphanistrum* | 39.29 | ± | 6.01 |
|  |  |  |  | *Brassica rapa* | 0.64 | ± | 0.32 |
|  |  |  |  | *Sinapis alba* | 10.34 | ± | 2.15 |
|  |  |  |  | *Sisymbrium officinale* | 66.68 | ± | 8.61 |
| Time in the field (weeks) | 11 | 511.17 | **< 0.0001** | *Capsella bursa-pastoris* | 4.86 | ± | 0.31 |
|  |  |  |  | *Camelina sativa* | 4.48 | ± | 0.12 |
|  |  |  |  | *Erysimum cheiranthoides* | 5.26 | ± | 0.15 |
|  |  |  |  | *Rorippa sylvestris* | 7.00 | ± | 0.15 |
|  |  |  |  | *Descurainia sophia* | 6.27 | ± | 0.23 |
|  |  |  |  | *Brassica nigra* | 5.78 | ± | 0.17 |
|  |  |  |  | *Sinapis arvensis* | 4.37 | ± | 0.18 |
|  |  |  |  | *Rapistrum rugosum* | 5.63 | ± | 0.15 |
|  |  |  |  | *Raphanus raphanistrum* | 5.62 | ± | 0.15 |
|  |  |  |  | *Brassica rapa* | 6.34 | ± | 0.15 |
|  |  |  |  | *Sinapis alba* | 5.14 | ± | 0.41 |
|  |  |  |  | *Sisymbrium officinale* | 6.87 | ± | 0.23 |

**Table S20.** Results of linear mixed models (LMM) testing differences in phenotypic traits across the two phylogenetic lineages. We included the plot in which the plant individuals were planted and plant species as random intercepts and allowed variance to be different for the different plant lineages to account for heterogeneity of variance. Significant *P* values (*P* < 0.05) are presented in bold.

| Parameter | df | *ꭓ*^2^ | *P* | Plant Lineage | Estimated mean |  | SE |
| --- | --- | --- | --- | --- | --- | --- | --- |
| Plant height (cm) | 1 | 1.62 | 0.2035 | I | 42.20 | ± | 11.62 |
|  |  |  |  | II | 61.60 | ± | 9.84 |
| Plant diameter (cm) | 1 | 6.54 | **0.0105** | I | 34.60 | ± | 7.87 |
|  |  |  |  | II | 61.10 | ± | 6.73 |
| Length of largest leaf (cm) | 1 | 4.73 | **0.0296** | I | 13.20 | ± | 2.72 |
|  |  |  |  | II | 20.90 | ± | 2.31 |
| Number of true leaves | 1 | 0.22 | 0.6364 | I | 233.00 | ± | 81.20 |
|  |  |  |  | II | 183.00 | ± | 68.60 |
| Number of flower axes | 1 | 3.57 | 0.0587 | I | 10.30 | ± | 10.39 |
|  |  |  |  | II | 36.40 | ± | 9.08 |
| Time in the field (weeks) | 1 | 0.01 | 0.9282 | I | 5.63 | ± | 0.39 |
|  |  |  |  | II | 5.68 | ± | 0.33 |

**Table S21.** Pairwise permutational comparisons of the multivariate phenotype of plant species. Analyses were performed by PERMANOVA using 999 permutations and accounting for dependency of observations. Values above the diagonals represent pseudo-*F* values, values below the diagonals represent *P* values adjusted with False Discovery Rate (FDR) correction. Significant *P* values (*P* < 0.05) are indicated in bold.

|  | *C. bursa-pastoris* | *C. sativa* | *E. cheiranthoides* | *R. sylvestris* | *D. sophia* | *B. nigra* | *S. arvensis* | *R. rugosum* | *R. raphanistrum* | *B. rapa* | *S. alba* | *S. officinale* |
| --- | --- | --- | --- | --- | --- | --- | --- | --- | --- | --- | --- | --- |
| *Capsella bursa-pastoris* |  | 28.29 | 43.93 | 133.47 | 14.67 | 30.48 | 17.46 | 65.63 | 30.43 | 53.96 | 28.10 | 54.22 |
| *Camelina sativa* | **0.0011** |  | 13.75 | 146.90 | 24.06 | 28.22 | 33.33 | 65.50 | 41.71 | 111.24 | 15.93 | 55.80 |
| *Erysimum cheiranthoides* | **0.0011** | **0.0051** |  | 80.51 | 32.21 | 24.36 | 68.86 | 48.63 | 40.51 | 110.20 | 37.27 | 40.63 |
| *Rorippa sylvestris* | **0.0011** | **0.0011** | **0.0011** |  | 19.37 | 26.10 | 253.78 | 67.29 | 39.08 | 52.90 | 114.32 | 41.41 |
| *Descurainia sophia* | **0.0011** | **0.0011** | **0.0011** | **0.0011** |  | 30.68 | 41.98 | 60.81 | 30.55 | 36.11 | 24.26 | 49.15 |
| *Brassica nigra* | **0.0011** | **0.0031** | **0.0011** | **0.0011** | **0.0011** |  | 46.74 | 1.72 | 9.27 | 35.84 | 28.24 | 17.06 |
| *Sinapis arvensis* | **0.0011** | **0.0011** | **0.0011** | **0.0011** | **0.0011** | 0.**0011** |  | 104.43 | 60.22 | 128.26 | 20.14 | 99.87 |
| *Rapistrum rugosum* | **0.0011** | **0.0011** | **0.0021** | **0.0011** | **0.0011** | 0.4350 | **0.0011** |  | 10.53 | 62.61 | 68.63 | 22.68 |
| *Raphanus raphanistrum* | **0.0011** | **0.0011** | **0.0011** | **0.0011** | **0.0011** | **0.0011** | **0.0011** | **0.0051** |  | 24.08 | 42.40 | 31.79 |
| *Brassica rapa* | **0.0011** | **0.0011** | **0.0011** | **0.0011** | **0.0011** | **0.0011** | **0.0011** | **0.0011** | **0.0011** |  | 101.20 | 53.58 |
| *Sinapis alba* | **0.0011** | **0.0011** | **0.0011** | **0.0011** | **0.0011** | **0.0011** | **0.0011** | **0.0011** | **0.0011** | **0.0011** |  | 74.33 |
| *Sisymbrium officinale* | **0.0011** | **0.0011** | **0.0011** | **0.0011** | **0.0011** | **0.0011** | **0.0011** | **0.0011** | **0.0011** | **0.0011** | **0.0011** |  |

**Table S22.** Total variation in herbivore community composition we could relate to plant phenotypic parameters. We applied unconstrained ordination (PCA) of incidence-based observations, followed by post-hoc regression of plant parameters against the three most important PCA axes. This can be compared to constrained ordination (RDA) of herbivore communities with these same plant parameters. Here, we present the percentage of total variation in herbivore composition that could be explained by the respective plant parameters.

|  | Unconstrained | | |  | Constrained | | |
| --- | --- | --- | --- | --- | --- | --- | --- |
| Community subset | Parameter | r^2^ | *P* |  | Parameter | % | *P* |
| Full herbivore community | Time in field | 0.6023 | 0.0010 |  | Time in field | 9.7179 | 0.0010 |
|  | Length largest leaf | 0.4051 | 0.0010 |  | Plant diameter | 5.6617 | 0.0010 |
|  | Plant diameter | 0.3902 | 0.0010 |  | Length largest leaf | 1.6708 | 0.0010 |
|  | Number flower axes | 0.2134 | 0.0010 |  | Number of leaves | 1.1174 | 0.0010 |
|  | Plant height | 0.2104 | 0.0010 |  | Plant height | 0.4212 | 0.0010 |
|  | Number of leaves | 0.1224 | 0.0010 |  | Number flower axes | 0.1458 | 0.0010 |
| Chewing herbivore community | Time in field | 0.3507 | 0.0010 |  | Time in field | 8.1396 | 0.0010 |
|  | Length largest leaf | 0.3015 | 0.0010 |  | Plant diameter | 7.4968 | 0.0010 |
|  | Plant diameter | 0.2605 | 0.0010 |  | Length largest leaf | 1.6525 | 0.0010 |
|  | Number flower axes | 0.1573 | 0.0010 |  | Number of leaves | 0.4945 | 0.0010 |
|  | Plant height | 0.1149 | 0.0010 |  | Plant height | 0.1483 | 0.0010 |
|  | Number of leaves | 0.1105 | 0.0010 |  | Number flower axes | 0.0678 | 0.0010 |
| Sap-feeding herbivore community | Time in field | 0.4693 | 0.0010 |  | Time in field | 19.6036 | 0.0010 |
|  | Length largest leaf | 0.3067 | 0.0010 |  | Plant diameter | 2.5063 | 0.0010 |
|  | Plant diameter | 0.2405 | 0.0010 |  | Length largest leaf | 0.7426 | 0.0010 |
|  | Number of leaves | 0.1086 | 0.0010 |  | Number of leaves | 0.1756 | 0.0010 |
|  | Plant height | 0.0656 | 0.0010 |  | Plant height | n.s. | n.s. |
|  | Number flower axes | 0.0655 | 0.0010 |  | Number flower axes | n.s. | n.s. |

**Table S23.** Total variation in herbivore community structure we could relate to plant phenotypic parameters. We applied unconstrained ordination (PCA) of log (x+1) transformed abundance observations, followed by post-hoc regression of plant parameters against the three most important PCA axes. This can be compared to constrained ordination (RDA) of herbivore communities with these same plant parameters. Here, we present the percentage of total variation in herbivore composition that could be explained by the respective plant parameters.

|  | Unconstrained | | |  | Constrained | | |
| --- | --- | --- | --- | --- | --- | --- | --- |
| Community subset | Parameter | r^2^ | *P* |  | Parameter | % | *P* |
| Full herbivore community | Time in field | 0.4392 | 0.0010 |  | Time in field | 15.9549 | 0.0010 |
|  | Length largest leaf | 0.3624 | 0.0010 |  | Plant diameter | 5.4400 | 0.0010 |
|  | Plant diameter | 0.2718 | 0.0010 |  | Length largest leaf | 2.9415 | 0.0010 |
|  | Number flower axes | 0.2661 | 0.0010 |  | Number of leaves | 1.2212 | 0.0010 |
|  | Plant height | 0.2350 | 0.0010 |  | Plant height | 0.5369 | 0.0010 |
|  | Number of leaves | 0.0705 | 0.0010 |  | Number flower axes | 0.0784 | 0.0010 |
| Chewing herbivore community | Time in field | 0.3442 | 0.0010 |  | Time in field | 10.3611 | 0.0010 |
|  | Length largest leaf | 0.2329 | 0.0010 |  | Plant diameter | 6.8349 | 0.0010 |
|  | Plant diameter | 0.1632 | 0.0010 |  | Length largest leaf | 2.4092 | 0.0010 |
|  | Number flower axes | 0.1155 | 0.0010 |  | Number of leaves | 1.1330 | 0.0010 |
|  | Plant height | 0.1037 | 0.0010 |  | Plant height | 0.1035 | 0.0010 |
|  | Number of leaves | 0.0896 | 0.0010 |  | Number flower axes | 0.0398 | 0.0010 |
| Sap-feeding herbivore community | Time in field | 0.4511 | 0.0010 |  | Time in field | 19.4873 | 0.0010 |
|  | Length largest leaf | 0.3813 | 0.0010 |  | Plant diameter | 4.2892 | 0.0010 |
|  | Plant diameter | 0.2892 | 0.0010 |  | Length largest leaf | 1.1327 | 0.0010 |
|  | Number of leaves | 0.1987 | 0.0010 |  | Number of leaves | 0.0049 | 0.0010 |
|  | Plant height | 0.1059 | 0.0010 |  | Plant height | n.s. | n.s. |
|  | Number flower axes | 0.0864 | 0.0010 |  | Number flower axes | n.s. | n.s. |

**Table S24.** Percentage of plant intra-specific variation in herbivore community composition we could relate to plant phenotypic parameters. Results are obtained by constrained ordination (RDA) of incidence-based observation data.

| Community  subset | Plant species | Time in field | Plant diameter | Number flower axes | Number of leaves | Length largest leaf | Plant height | Total variation |
| --- | --- | --- | --- | --- | --- | --- | --- | --- |
| Full herbivore community | *Capsella bursa-pastoris* | n.s. | n.s. | n.s. | n.s. | n.s. | n.s. | 0.00% |
|  | *Camelina sativa* | n.s. | n.s. | n.s. | n.s. | n.s. | n.s. | 0.00% |
|  | *Erysimum cheiranthoides* | n.s. | n.s. | 5.87% | n.s. | n.s. | 3.30% | 9.17% |
|  | *Rorippa sylvestris* | n.s. | n.s. | n.s. | n.s. | n.s. | n.s. | 0.00% |
|  | *Descurainia sophia* | n.s. | n.s. | n.s. | n.s. | n.s. | 11.86% | 11.86% |
|  | *Brassica nigra* | n.s. | n.s. | 10.48% | 4.85% | n.s. | 1.58% | 16.91% |
|  | *Sinapis arvensis* | n.s. | n.s. | 4.31% | n.s. | n.s. | n.s. | 4.31% |
|  | *Rapistrum rugosum* | 6.25% | n.s. | n.s. | n.s. | n.s. | n.s. | 6.25% |
|  | *Raphanus raphanistrum* | 3.06% | n.s. | n.s. | 7.58% | n.s. | n.s. | 10.64% |
|  | *Brassica rapa* | 10.29% | n.s. | 3.46% | 2.60% | n.s. | n.s. | 16.32% |
|  | *Sinapis alba* | n.s. | n.s. | n.s. | 8.88% | n.s. | n.s. | 8.88% |
|  | *Sisymbrium officinale* | n.s. | 4.29% | n.s. | n.s. | n.s. | n.s. | 4.29% |
| Chewing herbivore community | *Capsella bursa-pastoris* | n.s. | 8.37% | n.s. | n.s. | n.s. | n.s. | 8.37% |
|  | *Camelina sativa* | n.s. | n.s. | n.s. | n.s. | n.s. | n.s. | 0.00% |
|  | *Erysimum cheiranthoides* | n.s. | n.s. | n.s. | n.s. | n.s. | n.s. | 0.00% |
|  | *Rorippa sylvestris* | n.s. | n.s. | n.s. | 3.83% | n.s. | 6.31% | 10.14% |
|  | *Descurainia sophia* | n.s. | n.s. | n.s. | n.s. | n.s. | 10.63% | 10.63% |
|  | *Brassica nigra* | n.s. | n.s. | 11.59% | n.s. | n.s. | 2.15% | 13.74% |
|  | *Sinapis arvensis* | n.s. | n.s. | 5.88% | n.s. | n.s. | n.s. | 5.88% |
|  | *Rapistrum rugosum* | 4.98% | n.s. | n.s. | n.s. | n.s. | n.s. | 4.98% |
|  | *Raphanus raphanistrum* | 5.96% | n.s. | n.s. | 5.03% | n.s. | n.s. | 10.99% |
|  | *Brassica rapa* | n.s. | n.s. | n.s. | n.s. | n.s. | n.s. | 0.00% |
|  | *Sinapis alba* | 6.76% | n.s. | n.s. | n.s. | n.s. | n.s. | 6.76% |
|  | *Sisymbrium officinale* | n.s. | n.s. | n.s. | n.s. | n.s. | n.s. | 0.00% |
| Sap-feeding herbivore community | *Capsella bursa-pastoris* | n.s. | n.s. | n.s. | n.s. | n.s. | 9.07% | 9.07% |
|  | *Camelina sativa* | n.s. | n.s. | n.s. | n.s. | n.s. | n.s. | 0.00% |
|  | *Erysimum cheiranthoides* | n.s. | n.s. | n.s. | n.s. | n.s. | 14.90% | 14.90% |
|  | *Rorippa sylvestris* | n.s. | n.s. | n.s. | n.s. | n.s. | n.s. | 0.00% |
|  | *Descurainia sophia* | 19.70% | n.s. | n.s. | n.s. | n.s. | 4.40% | 24.10% |
|  | *Brassica nigra* | n.s. | n.s. | n.s. | 11.30% | n.s. | n.s. | 11.30% |
|  | *Sinapis arvensis* | n.s. | n.s. | n.s. | 6.85% | n.s. | n.s. | 6.85% |
|  | *Rapistrum rugosum* | n.s. | 11.76% | n.s. | n.s. | n.s. | n.s. | 11.76% |
|  | *Raphanus raphanistrum* | 1.30% | n.s. | n.s. | 12.00% | n.s. | n.s. | 13.30% |
|  | *Brassica rapa* | 27.15% | n.s. | 0.08% | 5.42% | n.s. | n.s. | 32.65% |
|  | *Sinapis alba* | n.s. | n.s. | n.s. | 9.40% | n.s. | n.s. | 9.40% |
|  | *Sisymbrium officinale* | n.s. | n.s. | n.s. | n.s. | 9.60% | 4.20% | 13.80% |

**Table S25.** Percentage of plant intra-specific variation in herbivore community structure we could relate to plant phenotypic parameters. Results are obtained by constrained ordination (RDA) of log (x + 1) transformed abundance observations.

| Community subset | Plant species | Time in field | Plant diameter | Number flower axes | Number of leaves | Length largest leaf | Plant height | Total variation |
| --- | --- | --- | --- | --- | --- | --- | --- | --- |
| Full herbivore community | *Capsella bursa-pastoris* | n.s. | 9.04% | n.s. | n.s. | n.s. | n.s. | 9.04% |
|  | *Camelina sativa* | n.s. | n.s. | n.s. | n.s. | n.s. | n.s. | 0.00% |
|  | *Erysimum cheiranthoides* | n.s. | n.s. | n.s. | 11.96 | n.s. | n.s. | 11.96% |
|  | *Rorippa sylvestris* | n.s. | n.s. | n.s. | 7.42% | n.s. | n.s. | 7.42% |
|  | *Descurainia sophia* | 4.72% | n.s. | n.s. | n.s. | n.s. | 15.90% | 20.63% |
|  | *Brassica nigra* | n.s. | n.s. | n.s. | n.s. | n.s. | 12.23% | 12.23% |
|  | *Sinapis arvensis* | 1.90% | n.s. | 4.89% | n.s. | n.s. | 8.12% | 14.91% |
|  | *Rapistrum rugosum* | n.s. | 11.93% | n.s. | n.s. | n.s. | n.s. | 11.93% |
|  | *Raphanus raphanistrum* | 11.10% | n.s. | n.s. | 2.67% | n.s. | n.s. | 13.77% |
|  | *Brassica rapa* | 34.17% | n.s. | n.s. | n.s. | n.s. | 2.16 | 36.33% |
|  | *Sinapis alba* | n.s. | n.s. | n.s. | 13.60% | n.s. | n.s. | 13.60% |
|  | *Sisymbrium officinale* | n.s. | n.s. | n.s. | 10.33% | n.s. | 5.02% | 15.35% |
| Chewing herbivore community | *Capsella bursa-pastoris* | n.s. | 13.47% | n.s. | n.s. | n.s. | n.s. | 13.47% |
|  | *Camelina sativa* | n.s. | n.s. | n.s. | n.s. | n.s. | n.s. | 0.00% |
|  | *Erysimum cheiranthoides* | n.s. | n.s. | n.s. | 9.00% | n.s. | n.s. | 9.00% |
|  | *Rorippa sylvestris* | n.s. | n.s. | n.s. | 4.63% | n.s. | 5.77% | 10.41% |
|  | *Descurainia sophia* | 10.20% | n.s. | n.s. | n.s. | n.s. | n.s. | 10.20% |
|  | *Brassica nigra* | n.s. | n.s. | 13.89% | n.s. | 3.07% | n.s. | 16.96% |
|  | *Sinapis arvensis* | n.s. | n.s. | n.s. | n.s. | n.s. | 6.17% | 6.17% |
|  | *Rapistrum rugosum* | n.s. | n.s. | 13.66% | n.s. | n.s. | 1.38% | 15.04% |
|  | *Raphanus raphanistrum* | 7.81% | 1.65% | n.s. | 2.67% | n.s. | n.s. | 27.41% |
|  | *Brassica rapa* | n.s. | n.s. | 4.63% | n.s. | n.s. | n.s. | 4.63% |
|  | *Sinapis alba* | n.s. | n.s. | n.s. | 16.03% | 2.64% | n.s. | 18.67% |
|  | *Sisymbrium officinale* | n.s. | n.s. | 7.91% | n.s. | n.s. | n.s. | 7.91% |
| Sap-feeding herbivore community | *Capsella bursa-pastoris* | n.s. | 0.14% | 8.69% | n.s. | n.s. | 27.70% | 36.09% |
|  | *Camelina sativa* | n.s. | n.s. | n.s. | 16.09% | n.s. | n.s. | 16.09% |
|  | *Erysimum cheiranthoides* | n.s. | n.s. | n.s. | n.s. | n.s. | n.s. | 0.00% |
|  | *Rorippa sylvestris* | n.s. | n.s. | n.s. | 11.57% | n.s. | n.s. | 11.57% |
|  | *Descurainia sophia* | 10.12% | n.s. | n.s. | n.s. | n.s. | 16.70% | 26.82% |
|  | *Brassica nigra* | n.s. | n.s. | 9.31% | n.s. | n.s. | n.s. | 9.31% |
|  | *Sinapis arvensis* | n.s. | n.s. | n.s. | n.s. | n.s. | n.s. | 0.00% |
|  | *Rapistrum rugosum* | n.s. | 14.64% | n.s. | n.s. | n.s. | n.s. | 14.64% |
|  | *Raphanus raphanistrum* | n.s. | n.s. | n.s. | n.s. | n.s. | n.s. | 0.00% |
|  | *Brassica rapa* | 44.75% | n.s. | n.s. | n.s. | n.s. | 1.01% | 45.77% |
|  | *Sinapis alba* | n.s. | n.s. | n.s. | n.s. | n.s. | n.s. | 0.00% |
|  | *Sisymbrium officinale* | n.s. | n.s. | n.s. | 17.20% | n.s. | 7.70% | 24.90% |
